# Genetic dissection of the soybean dwarf mutant *dm* with integrated genomic, transcriptomic and methylomic analyses

**DOI:** 10.3389/fpls.2022.1017672

**Published:** 2022-11-21

**Authors:** Jian Song, Xuewen Wang, Lan Huang, Zhongfeng Li, Honglei Ren, Jun Wang

**Affiliations:** ^1^ College of Life Science, Yangtze University, Jingzhou, China; ^2^ National Key Facility for Gene Resources and Genetic Improvement (NFCRI)/Institute of Crop Sciences, Chinese Academy of Agricultural Sciences, Beijing, China; ^3^ Department of Genetics, University of Georgia, Athens, AB, United States; ^4^ Department of Computer Science, Yangtze University, Jingzhou, China; ^5^ Soybean Research Institute, Heilongjiang Academy of Agricultural Sciences, Harbin, China; ^6^ College of Agriculture, Yangtze University, Jingzhou, China

**Keywords:** soybean, dwarf, methylation, DNA variation, transcriptome, auxin, cellulose

## Abstract

Plant height affects crop production and breeding practices, while genetic control of dwarfism draws a broad interest of researchers. Dwarfism in soybean (*Glycine max*) is mainly unexplored. Here, we characterized a dwarf mutant *dm* screened from ethyl methanesulfonate (EMS) mutated seeds of the soybean cultivar Zhongpin 661(ZP). Phenotypically, *dm* showed shorter and thinner stems, smaller leaves, and more nodes than ZP under greenhouse conditions. Genetically, whole-genome sequencing and comparison revealed that 210K variants of SNPs and InDel in ZP relative to the soybean reference genome Williams82, and EMS mutagenesis affected 636 genes with variants predicted to have a large impact on protein function in *dm*. Whole-genome methylation sequencing found 704 differentially methylated regions in *dm*. Further whole-genome RNA-Seq based transcriptomic comparison between ZP and *dm* leaves revealed 687 differentially expressed genes (DEGs), including 263 up-regulated and 424 down-regulated genes. Integrated omics analyses revealed 11 genes with both differential expressions and DNA variants, one gene with differential expression and differential methylation, and three genes with differential methylation and sequence variation, worthy of future investigation. Genes in cellulose, fatty acids, and energy-associated processes could be the key candidate genes for the dwarf phenotype. This study provides genetic clues for further understanding of the genetic control of dwarfism in soybean. The genetic resources could help to inbreed new cultivars with a desirable dwarf characteristic.

## Introduction

Dwarfism, a reduced plant height, is a desirable characteristic in crop breeding for improved response to fertilizer input, lodging resistance, enhanced light utilization, etc. (Cooper et al., 1995; Cooper et al., 2003; Hedden, 2003; Wei et al., 2021; Su et al., 2022). Semi-dwarf varieties have been introduced in many crop species, e.g., wheat, rice, and soybean, such as Hobbit87, Charleston, and Apex (Peng et al., 1999; Cooper et al., 1995; Khush, 2001; Cooper et al., 2003; Hedden, 2003). The mechanism of dwarfism in plant is complicated. Studies show that the genetic control of plant height can be regulated by a single dwarf gene or multiple associated genetic loci or genes (Kuraparthy et al., 2008; Dardick et al., 2013; Roy et al., 2021). Some of the dwarf genes are closely related to hormone signal pathways, like gibberellin (GA), brassinosteroids (BRs), auxin (IAA) and/or strigolactone (SLs) (Itoh et al., 2004; Lin et al., 2009; Hirano et al., 2010; Tong et al., 2010; Sun, 2011; Yang et al., 2011; Li et al., 2014; Zhou et al., 2015; Li et al., 2018b; Zhao et al., 2019; Roy et al., 2021; Su et al., 2022; Xu et al., 2022).

Soybean (*Glycine max*) is a legume species and has become one of the most important crops for food and studies on symbioses. Soybean genomic sequence is publicly available (Schmutz et al., 2010). Only a few dwarf genes or loci in the genome have been reported, although the dwarf is preferred in soybean breeding processes (Reinhardt and Kuhlemeier, 2002; Liu et al., 2020). Dwarf mutant *dw* harbors a mutation in the *GmDW1* gene, encoding Kaurene Synthase, in the GA biosynthesis pathway (Li et al., 2018b). A dwarf mutant from fast neutron bombardment has an 803 bp deletion in the first exon of *Glyma15g05831* (Hwang et al., 2015). The *sdf-1* locus was mapped to an 80.72 kb region on Chr 19 of soybean (Dong et al., 2020). *GmIAA27* relies on auxin to interact with TIR1, and control a *dmbn* mutant plants (Su et al., 2022). The challenge of genetic dissection of the dwarf in soybean may result from palaeopolyploid, which makes dwarf gene identification in soybean more complicated.

Only several dwarf soybean mutants have been created with methods like gamma rays, fast neutron radiation, and chemical mutagenesis, e.g., ethyl methanesulfonate (EMS) (Zhang et al., 2014a; Hwang et al., 2015; Cheng et al., 2016; Li et al., 2018b; Mestanza et al., 2019; Xiong et al., 2020). EMS mutagenesis primarily causes single nucleotide mutation and is widely used to create novel mutants and identify mutation associated genes (Zhu et al., 2005; Cui et al., 2013; Li et al., 2017).

Mutation in DNA is the initial cause of dwarf. As an epigenetic modification of DNA, DNA methylation affects gene expression and phenotype (Morgan et al., 2022; Wei et al., 2022). Cytosine methylation is the most common modification, in which a methyl group is added to the carbon 5-position of cytosine bases (5mdC) (Cokus et al., 2008; Saze et al., 2012; Hardcastle, 2013). mCG, mCHG, and mCHH are the most common DNA methylations in the genome (Lindroth et al., 2001; Stroud et al., 2014; Al Harrasi et al., 2018). DNA methylation is widespread in plant genomes, dynamically maintains genome stability and regulates gene expression (Zhang et al., 2018). No matter of DNA mutation or modification, both affect gene’s transcription. The advances in high-throughput sequencing technologies enable the detection of genome-scale nucleotide mutation in mutants, measurement of regulation at transcriptional levels *via* RNA-Seq, and detection of methylation level at low cost and in a fast way (Jain et al., 2014; Lin et al., 2015; Zhou et al., 2016; Liu et al., 2017; Yan et al., 2017; Li et al., 2018a; Zhao et al., 2021).

In this study, a dwarf soybean mutant (*dm*) was isolated from EMS mutagenized lines from the cultivar Zhongpin 661, and phenotypes were characterized. To understand the molecular bases of the dwarf phenotype, we compared the differences between *dm* and Zhongpin 661 with whole genome sequencing analysis, RNA-Seq based transcriptomic analysis, whole genomic methylation analysis, and metabolite contents to target genetic dissection of dwarfism in soybean. Integrated omics analyses were then targeted to dissect the genetic cause of dwarfism in soybean. Results showed that eleven candidate genes in processes of cellulose, fatty acids and energy are associated with the dwarf phenotype. Our results provided a comprehensive comparison and clues for candidate genes for dwarfism in *dm*.

## Materials and methods

### Plant material, plant growth, and phenotypes

The soybean dwarf mutant M_2_ line was selected from EMS-induced mutants of cultivar Zhongpin 661 (Li et al., 2017; Li et al., 2018b). Plants were germinated on plate and transplanted into soil in greenhouse with the same growth conditions. Progenies of dwarf mutant and WT control were planted in Changping base of Chinese Academy of Agricultural Science in the summer of 2014. After harvesting seeds, the stems of plants were collected for cellulose content determination. Six plant individuals were phenotyped for plant height, stem, leaf size, branch and node number at different developmental stages. Fresh leaves from the third node were sampled for genome resequencing, RNA-seq analysis, and methyl-seq at V5 stage.

### DNA isolation, library construction, and genomic resequencing analysis

Genomic DNA was isolated from collected leaves of the mutant and Zhongpin 661 with a genomic DNA purification kit (Thermo Fisher Scientific Inc., United States) according to the manufacturer’s protocol. DNA samples were quantified using a Quawell Q5000 spectrophotometer (Quawell Technology, Inc., United States). The DNA from the dwarf mutant and Zhongpin661 were used to construct paired-end sequencing libraries, which were sequenced on an Illumina HiSeq-2500 platform independently. After removing adapters and low-quality sequence reads, the clean reads were further rechecked for quality with FastQC (https://www.bioinformatics.babraham.ac.uk/projects/fastqc/). High-quality reads were aligned and mapped to the Glycine max Wm82.a2.v1 reference genome (Schmutz et al., 2010) downloaded from Phytozome (http://phytozome.jgi.doe.gov/pz/portal.html#!info?alias=Org_Gmax)with BWA (version 0.7.1) with default parameters (Li and Durbin, 2009). GATK (Genome Analysis Toolkit, version 4) was used to call SNPs and small InDels (< 50 bp) across the mutant pool and wild type pool with a minimum read coverage great than two (Mckenna et al., 2010).

### RNA isolation, library construction, and transcriptomic analysis

Around 3 μg RNA extracted by TriZol (ThermoFisher, Cat. 15596026) was used for sequencing library preparation with NEBNext^®^ Ultra™ RNA Library Prep Kit for Illumina^®^ (NEB, USA) according to the manufacturer’s recommendations. The libraries were sequenced on an Illumina HiSeq 2500 platform and 100 bp paired-end raw reads were generated.

Raw reads were firstly processed to filter out adapter, ploy-N, and low-quality reads. The clean reads with high quality were mapped to the soybean genome (Schmutz et al., 2010) with TopHat 2 (Kim et al., 2013). The read counts of each gene were adjusted with EdgeR through one scaling normalized factor (Mortazavi et al., 2008). Differential expression analysis between mutant and WT was performed with the DEGseq R package (1.12.0) (Wang et al., 2010). The P-values were adjusted using the Benjamini & Hochberg method. Corrected P-value less than 0.005 and fold changes greater than two were set as the threshold for significantly differential expression.

### Methylation determination

MethylC-seq which differentiates cytosine from 5-methylcytosine was used to examine the methylation in a DNA sample (Urich et al., 2015). DNA was extracted by CTAB and then quantified using Qubit^®^ DNA Assay Kit in Qubit^®^ 2.0 Fluorometer (Life Technologies, CA, USA). For library construction, fragmented DNA of 200-300 bp in length was obtained by sonication of a total amount of 5.2 μg genomic DNA on Covaris S220, followed by end repair and adenylation. Then these DNA fragments were treated twice with bisulfite with EZ DNA Methylation-Gold Kit™ (Zymo Research). Then the single-strand fragments were enriched by PCR amplification with KAPA HiFi HotStart Uracil + ReadyMix(2X). The libraries were sequenced on an Illumina HiSeq-2500 platform to generate 100-bp paired-end raw reads.

Raw reads were processed to remove adaptor sequence, oligo-N containing sequence (>10%), and low quality (PHRED score<=5, and percentage of the low-quality bases >=50%) to create clean reads. These clean reads were aligned to the indexed soybean reference genome (As above) using Bowtie2 (Langmead and Salzberg, 2012) and Bismark (version 0.12.5; (Krueger and Andrews, 2011). The alignments were both performed in bisulfite-converted format with C-to-T and G-to-A.

To estimate the methylation level, we identified the methylation site using $s_{i,j}^{+}\sim Bin\left(s_{i,j}^{+}+s_{i,j}^{-},{\text{r}}_{i,j}\right)$ , in which the sum( $s_{i,j}^{+}$ ) of methylated counts was modeled as a binomial (Bin) random variable with methylation rate(r_*i*,*j*_ ). A sliding-window approach with window size of 3,000 bp and step size of 600 bp was used to calculate the sum of methylated and unmethylated read counts (Smallwood et al., 2014). Methylation level (ML) for each C site shows the fraction of methylated Cs, and is defined as  *ML*(C)=*reads*(mC)/[*reads*(*m*C)+*reads*(C)] . ML was then corrected by  *ML*_(*corrected*)_=(*ML*−*r*)/(1−*r*) , where r represents the bisulfite non-conversion rate according a previous description (Lister et al., 2013).

### GO enrichment analysis of differentially expressed genes

Gene Ontology (GO) enrichment analysis of differentially expressed genes was conducted with the GOseq R package, in which gene length bias was corrected (Young et al., 2010). Corrected *P*-value less than 0.05 were regarded as significantly enriched.

### Fiber content determination

The cellulose associated compounds in the stem were measured for ZP661 and dwarf mutant according to the national and agriculture standards. Briefly, the crude fiber was measured with methods described in GB/T6434-2006/ISO6865:2000 (GB/T6434, 2006); the acidic detergent fiber in GB/T20805-2006 (GB/T20805, 2006) and neutral detergent fiber were measured with methods described in GB/T20806-2006 (GB/T20806, 2006).

### CpG island (CGI) prediction

To predict CpG island region of DEGs, genomic sequence of a given gene, spanning from promoter sequence (-2000 bp) to 3’ UTR, was extracted from the soybean genome (*Wm82.a2.v1*). Then CGI region in the extracted sequence was predicted with the online tool Newcpgreport (http://www.ebi.ac.uk/Tools/seqstats/emboss_newcpgreport/). Methylation site in each context within CGI region was counted for further analysis.

## Results

### Phenotype of dwarf soybean mutant

**Figure 1:**
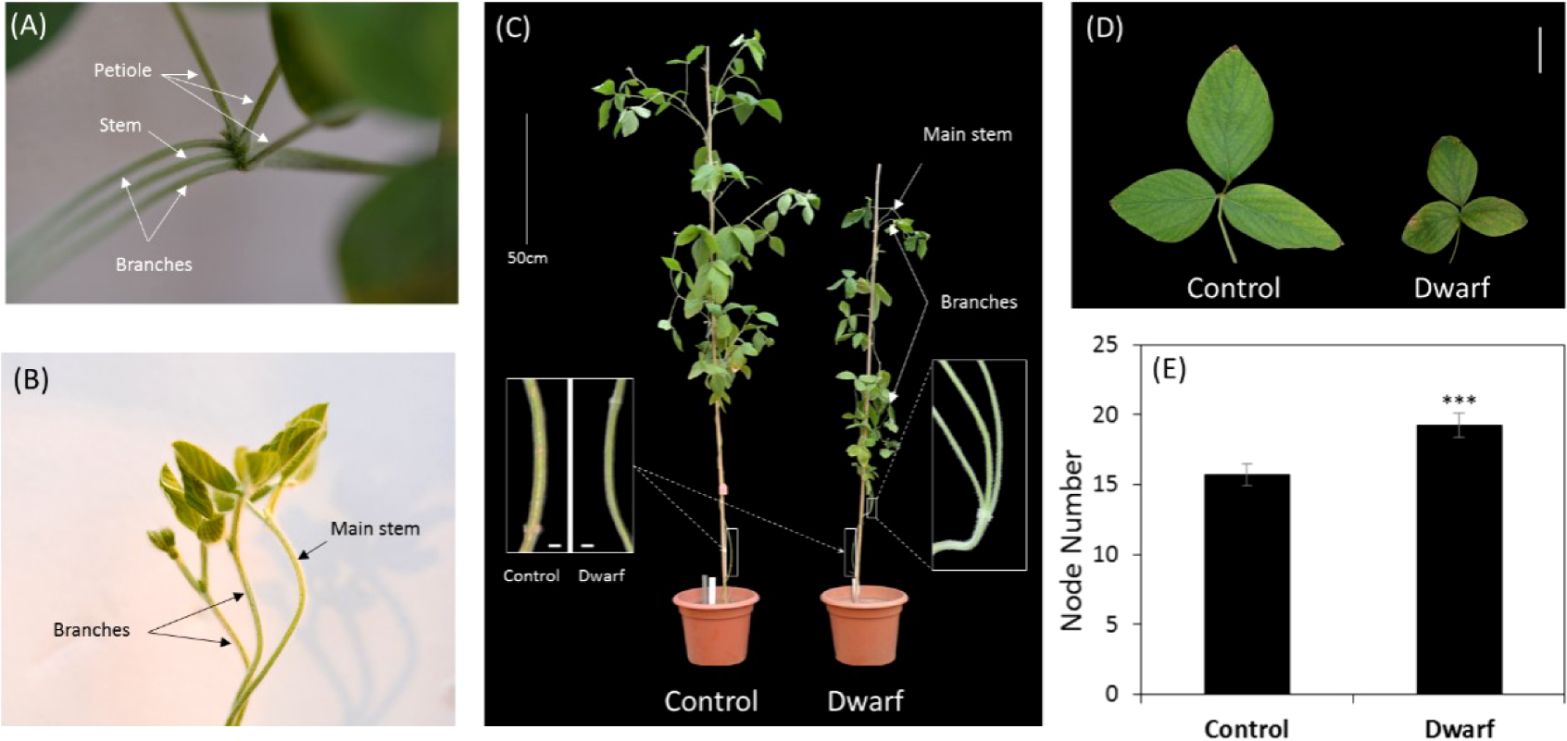
Phenotypic characterization of soybean dwarf mutant. **(A)** Two branches and three petioles on the fourth node of dwarf mutant (V6 stage), as indicated by white arrows. **(B)** Top view of branches from the fourth node (V6 stage), as indicated by black arrows. **(C)** Full view of dwarf mutant and control (R2 stage). Stem thickness was shown for the third node in the left rectangle; Branches of dwarf mutant was enlarged in the right rectangle, and branch apexes were indicated by white arrows. **(D)** leaf size of the ninth node (R5 stage), scale bar =5 cm. **(E)** Comparison of node number of M_3_ plants of the dwarf mutant and control, n=9,*** represents Student’s t-test P< 0.001.

A soybean dwarf mutant (*dm*) was screened from an M2 population derived from EMS mutagenized cultivar Zhongping661 (ZP) seed lines. This *dm* showed a stunted growth with a much shorter plant height (**Figure 1C**), thinner stem (**Figure 1C**), and smaller leaf size (**Figure 1D**) than the control ZP. Interestingly two branches and three leaves were juxtaposed at the fourth node of *dm*, and the lengths of the two branches were almost the same as the main stem (**Figures 1A–C**), as compared with control with only one trifoliolate and without branch on the fourth node. The node number of M3 plants of this dwarf mutant was significantly greater than that of the control (**Figure 1E**). No other difference was found between the mutant *dm* and control ZP.

### Genomic DNA variation between dwarf mutant and control

**Figure 2:**
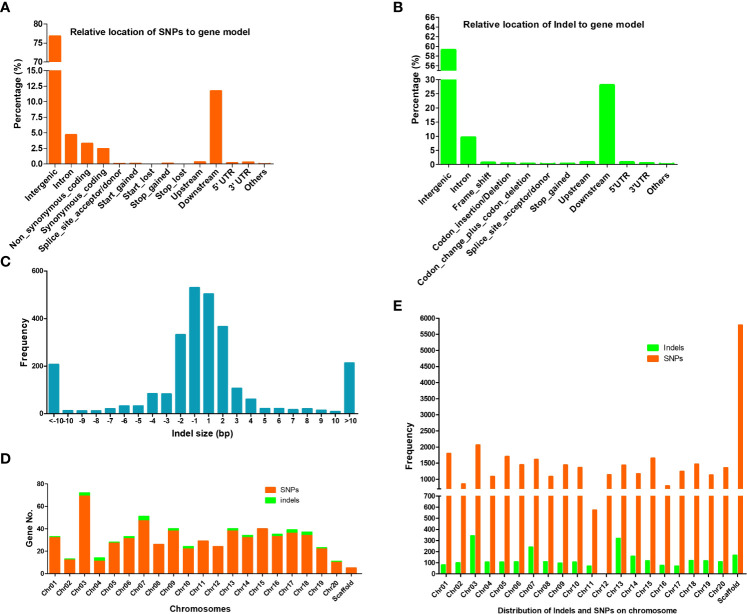
SNPs and indels in gene models and on chromosomes. **(A)** Relative location of SNPs to gene model. **(B)** Relative location of indels to gene model. **(C)** Indels size-frequency distribution. **(D)** Distribution of mutated genes caused by SNPs (Nonsynonymous substitution and alternative splicing) and indels (codon insertion/deletion, frame shift and alternative splicing) on soybean chromosomes. **(E)** Distribution of SNPs and indels on soybean chromosomes.

The whole genomic sequence of soybean ZP is not available so the generation of the DNA sequence of this cultivar is needed. To understand the DNA variation caused by EMS, DNA in the *dm* and control plant ZP were sequenced with an Illumina HiSeq 2500 platform to generate ~10 x average depth of short sequencing reads (**Table 1**). We focused on SNP and InDel variants resulting from EMS mutagenesis. After mapping reads to soybean reference genome Williams82, ~210,000 SNPs in total were identified with GATK (Mckenna et al., 2010) from both the *dm* and control compared with Williams82, suggesting lots of differences from Williams82. However, only 32,231 SNPs (~34 kb/SNP) were presented between the *dm* and the control, which should come from mutagenesis. Approximate 94.6% of these SNPs remained heterozygous. Transition (A↔G or T↔C) overtook transversion (A↔T or G↔C) with Ti/Tv =1.43 in the dwarf mutant (**Supplementary Table 1**). Relative to annotated gene models in *Wm82.a2.v1*, most of these SNPs (76.78%) were distributed among intergenic regions; however, only a small number of SNPs (0.29%) caused gene structure variation *via* synonymous or nonsynonymous substitution, transcriptional start site gained or lost, stop codon gained or lost, and splice site acceptor or donor change (first 2 bp or last 2 bp of an exon) etc. In total, these SNPs resulted in 600 nonsynonymous substitutions and 18 alternative splicing within genes (**Figure 2**). Most of these genes were unequally scattered on 20 chromosomes, with the most mutated genes (96) on Chr03 (**Figure 2D**; **Supplementary Table 2**). It is interesting that around 11.7% of SNPs are located within 5 kb of 3’ prime downstream of genes.

**Table 1:** Statistical summary of sequencing datasets.

Dataset	Items	Control	Dwarf mutant
RNA-seq	Total reads	61,470,696	65,527,760
	Mapped ratio (%)	93.1	93.3
	Multiple mapped ratio (%)	2.5	2.2
	Uniquely mapped ratio (%)	90.6	91.1
Resequencing	Total reads	121,650,152	109,272,844
	Quality value >30% ratio (%)	90.94	89.25
	Mapped ratio (%)	95.4	95.1
	Average sequencing depth	11×	10×
	Coverage ratio (%)	98.3	97.8
MethylC-seq	Total reads	137,500,853	127,681,600
	Mapping rate (%)	66.5	72.2
	Bisulfite conversion rate (%)	99.96	99.97
	1× C coverage (%)	93.4	93.0
	5× C coverage (%)	88.0	87.5

A total of 2716 small InDels (< 50 bp), including 1356 insertions and 1350 deletions, were detected between the *dm* and the control (**Figure 2C**). The indels were distributed on 19 chromosomes except for Chr12 (**Figure 2E**). Interestingly, the utmost indels are located on Chr03, which coincides with SNPs. Most of these indels are located on intergenic regions (~59.2%). For genic indels, around 27.98% preferentially occurred in a gene’s downstream region (< 5 kb) on the 3’ prime, and ~9.5% within introns. Only ~1.4% are located within exon, which led to codon change and alternative splicing of 33 genes (**Supplementary Table 3**).

**Figure 3:**
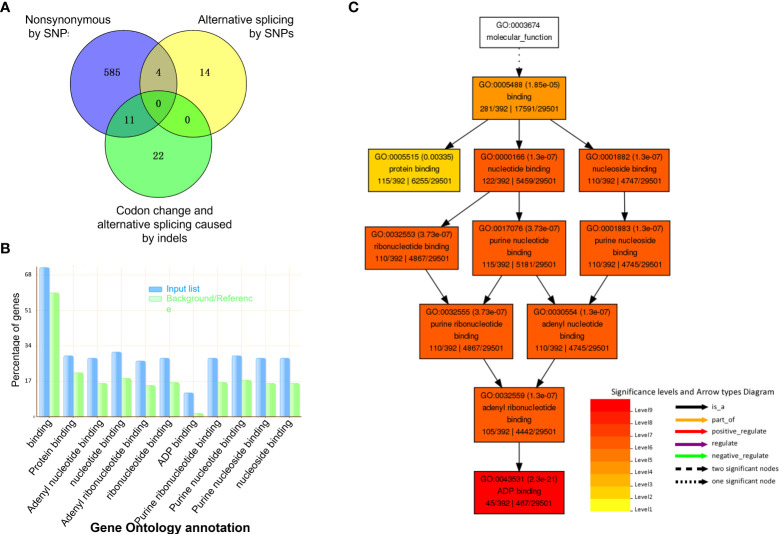
Gene Ontology (GO) analysis of mutated genes with InDels and SNPs. **(A)** Venn graph shows the number of genes in three types of predicted effects from mutations. **(B)** GO terms enriched by Fisher test at significance level< 0.05 and 5 minimum number of mapping entry. **(C)** Hierarchy structure of enriched GO terms.

Of these mutated genes with SNPs and indels, 15 contained two types of critical mutations of nonsynonymous substitution, alternative splicing, and codon change (**Figure 3A**). Taken together, genomic variation resulted in 636 mutated genes. Of those, 44.2% (281 out of 636 genes) genes had known GOs, and their molecular function of GOs were enriched in binding activity toward protein, ADP, nucleotide/nucleoside, purine ribonucleotide/ribonucleoside, and adenyl ribonucleotide/ribonucleoside (**Figures 3B, C**).

### Transcriptomic regulation in leaf between dwarf mutant and control

**Figure 4:**
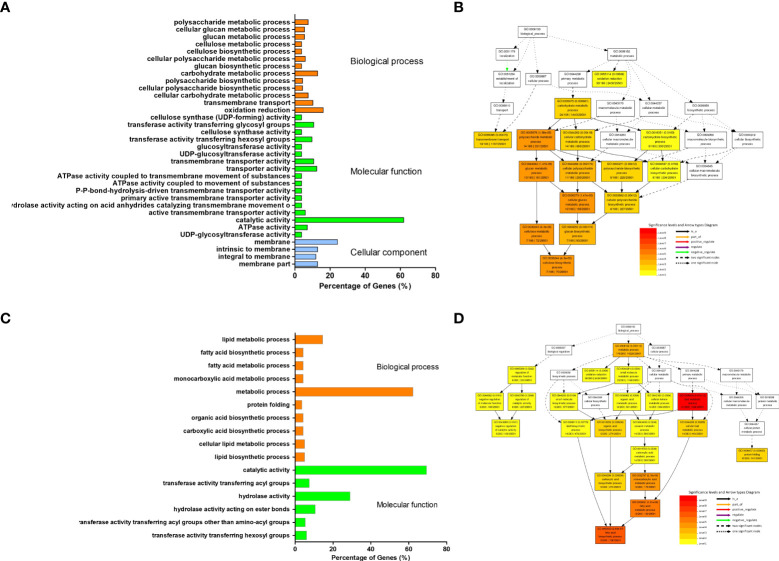
Categorization of differentially expressed genes between dwarf mutant and control. **(A, C)** GO enriched categories of up- and down-regulated DEGs, respectively. 470 out of 686 input genes were annotated with GO terms. **(B, D)** Directed acyclic graph (DAG) of biological process enriched by GO analysis of up- and down-regulated DEGs, respectively. DAG of the biological processes was reconstructed using top 10 enriched GO categories; the significant level of each category was labelled on map, each node for a GO term, and the color represents enrichment degree, the darker the higher enrichment.

To obtain transcriptional regulation on the dwarf phenotype, we performed RNA-Seq analysis of transcriptomes in leaves from the third node of the *dm* and the control at the six unfolded trifoliolate leaves (V6) stage. Over 60 Mb clean Illumina paired-end short reads were generated from the dwarf mutant and control ZP independently. After mapping these reads to the soybean reference genome (*Williams82.a2.v1*), the gene’s expression level was evaluated by calculating Fragment Per Kilo bases per Million reads (FPKM) (**Table 1**). In total, 424 down-regulated and 263 up-regulated differentially expressed genes (DEGs) were identified in the *dm* relative to the control with at least two folds of expressional change and corrected P-value< 0.005 (**Supplementary Figure S1**). GO analysis revealed that these up-regulated DEGs were significantly (Fisher test P*<* 0.01 and FDR< 0.01) enriched in 34 GOs, which were mostly related to cell glucan, cellulose and polysaccharide metabolism, biosynthesis, and energy associated activities(**Figures 4A, B**; **Supplementary Table 4**). In contrast, down-regulated DEGs were mostly enriched in 16 GO categories (Fisher test P< 0.01 and FDR< 0.01), mostly related to fatty acid synthesis **Figures 4C, D**; **Supplementary Table 5**).

### Changes in DNA methylation in dwarf mutant

**Figure 5:**
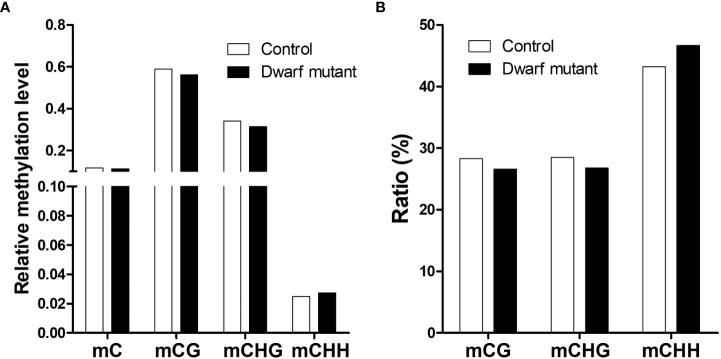
Genome-wide methylation status in dwarf mutant and control. **(A)** Relative methylation level defined as the ratio of methylated cytosines to total cytosines in each context. **(B)** Ratio (%) of methylated cytosines in different contexts to total methylated cytosines; genome-wide methylated cytosine is set as 100%. mC, mCG, mCHG, mCHG represents methylated cytosine in C (genome-wide), CG, CHG, CHH context, respectively.

To evaluate epigenetic regulation, we measured the genome-wide DNA methylation status in leaves, the same material used for RNA-seq and DNA sequencing, of the dwarf mutant *dm* and control. In total, ~137.5 Mb and ~127.7 Mb clean reads were generated for each line, meaning ~16 x coverage of soybean genome on average (**Table 1** *;* **Supplementary Figure S2**). Comparison analysis found that the DNA methylation level in the dwarf mutant was slightly lower than that in control although ~11% of all cytosines in genome had been methylated in both samples (**Figure 5**). Generally, ~57.6% (C:58.96%, M:56.24%), ~32.8% (C:34.12%, M:31.47%) and ~2.6% (C:2.48%, M:2.73%) of cytosines were methylated in CG, CHG and CHH context, respectively (**Figure 5**). The dwarf mutant had slightly fewer methylated cytosines at CG and CHG sites but more at CHH than control, in terms of both methylation levels or relative percentage of methylation in each context (**Figure 5**). A total of 704 differentially methylated regions (DMR) spanning from 45 bp to 21.89 kb were identified between *dm* and control, among which 119 and 343 genes were predicted on chromosomes and unanchored scaffolds, respectively. The unanchored scaffolds are mostly repeat sequence-like transposons, so those genes will most likely not affect the dwarf phenotype. These 119 chromosomal genes’ transcription could be affected by methylation alternation, theoretically inhibited, which we further investigated.

### Integrated effects of variants, transcriptome and methylome on dwarf

**Figure 6:**
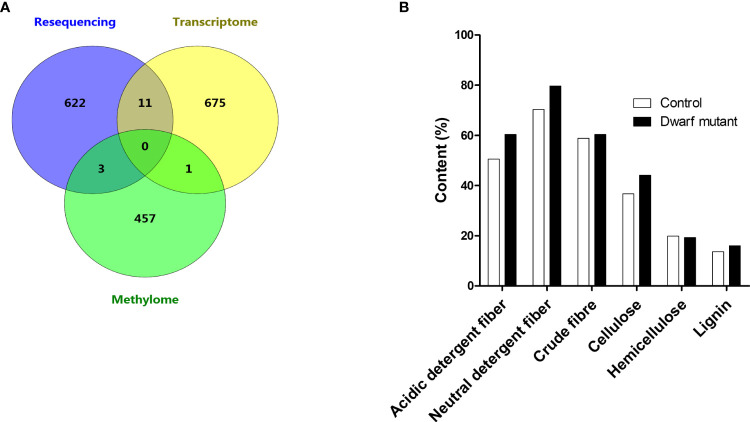
Integrative analysis among variants, transcriptome, and methylome. **(A)** Venn map showing the distribution of genes with nonsynonymous mutation in resequencing dataset, differentially expressed gene in transcriptome dataset and differentially methylated regions in methylome dataset. **(B)** Comparison of fiber contents between dwarf mutant and control.

To better understand the crossing molecular regulation in the dwarf mutant *dm*, we further checked the genes affected by any two combinations of variants, transcriptome, and methylome. Eleven genes, ~1.6% of DEGs, were found to have both nonsynonymous mutations and differential expression, which suggested that these genes’ expression was most likely directly regulated by mutations (**Figure 6A**). Thus, these genes were worthy of further investigation. The remaining 675 DEGs (98%), except one DEG, didn’t have nonsynonymous mutation and didn’t belong to DMR; therefore, they may be indirectly regulated by others, e.g., the cascaded signal of mutated genes. Three DMRs were found to also contain genes with nonsynonymous mutation(s), which were also important candidates for this dwarf phenotype (**Figure 6A**). All these mentioned genes were further checked in terms of expression level and functional prediction. Among these, a cellulose synthase gene (*Glyma.15G157100*) was included, which is known to catalyze beta-1,4-glucan cellulose microfibril for the secondary cell wall formation in the plant cellulose biosynthesis and in glycan metabolism (Mcfarlane et al., 2014). This gene expression in the dwarf mutant was increased by 3.5 folds (**Table 2**). However, a previous study showed that a mutation in this gene reduced cellulose levels and caused a dwarf phenotype (Holland et al., 2000). So a further chemical analysis of cellulose related compounds was conducted and revealed that acidic detergent fiber, neutral detergent fiber, crude fiber, cellulose and lignin in the stem of the dwarf mutant were 19.6%, 13.3%, 2.5%, 20% and 17.3%, and were higher than that of control, respectively, but hemicellulose content was 2.8% lower than that in control (**Figure 6B**). We hypothesized that the mutation may affect the cellulose balance in *dm*, which led to dwarfism.

**Table 2:** Expression level of common genes identified from resequencing, transcriptome and methylome datasets.

Classification	Gene ID	Control	Dwarf mutant	Logic (fold change)	*P*-value	Corrected *P-*value	Functional description
R vs T	Glyma.02G287400	20.37	0.00	-6.90	3.34E-06	2.10E-04	protodermal factor 1
	Glyma.03G148300	11.78	40.02	1.76	8.68E-05	3.73E-03	alpha/beta-Hydrolases superfamily protein
	Glyma.07G126600	71.62	3.25	-4.46	6.08E-18	1.43E-15	myo-inositol oxygenase 2/myo-inositol oxygenase 4
	Glyma.08G263200	5.23	28.50	2.44	3.97E-05	1.89E-03	DNA glycosylase superfamily protein
	Glyma.08G329900	75.75	23.92	-1.66	4.61E-08	4.14E-06	FASCICLIN-like arabinogalactan 1
	Glyma.09G188600	76.89	21.94	-1.81	5.61E-09	5.84E-07	Leucine-rich repeat (LRR) family protein
	Glyma.10G019000	185.60	453.26	1.29	3.39E-25	1.40E-22	multidrug resistance-associated protein 4
	Glyma.13G312900	61.04	153.91	1.33	5.07E-10	6.03E-08	BEL1-like homeodomain 1
	Glyma.15G157100	21.18	74.94	1.82	3.86E-08	3.52E-06	cellulose synthase A4
	Glyma.16G172600	59.59	10.32	-2.53	3.23E-10	3.92E-08	multidrug resistance-associated protein 14
	Glyma.17G138300	62.95	23.41	-1.43	7.67E-06	4.49E-04	Cupredoxin superfamily protein
T vs M	Glyma.04G196400	13.79	46.13	1.74	2.98E-05	1.48E-03	unknown protein
R vs M	Glyma.U005000	0.00	0.00	0.44	9.31E-01	1.00E+00	TMV resistance protein N-like
	Glyma.U032800	10.26	11.86	0.21	7.90E-01	1.00E+00	unknown protein
	Glyma.U043000	NA	NA	NA	NA	NA	unknown protein

Resequencing (R), transcriptome (T) and methylome (M), Control and dwarf mutant represents read count in each sample. Pvalue<0.05 and corrected Pvalue<0.001 is regarded as significant. The P-value is calculated by the binomial distribution test.

NA, Not Applicable.

**Figure 7:**
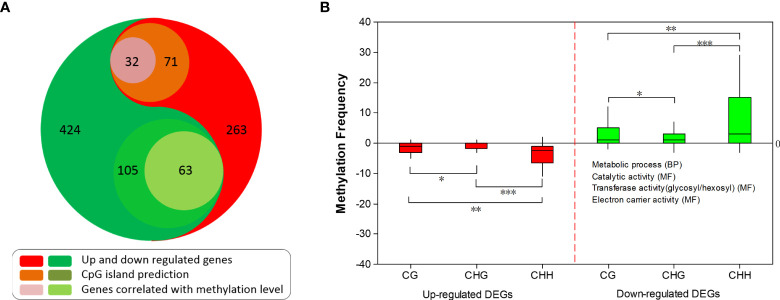
CGI prediction and correlation between methylation and expression of differentially expressed genes (DEGs). **(A)** Number of DEGs and DEGs with CGI prediction. **(B)** Methylation frequency of up-regulated and down-regulated DEGs. *, **, *** represents significant level of Student’s *t-*test of 0.05, 0.01, 0.001, respectively. Enriched GO categories of correlated DEGs are listed.

Methylation of CpG island (CGI) affects the targeting capability of transcriptional factors, which can change gene expression (Candaele et al., 2014; Zhang et al., 2014b). To better elucidate the possible correlation between CGI methylation and gene expression level in our study, CGI was predicted for each DEG (-2000 bp to 3’UTR) with Newcpgreport. CGI was found in 71 up-regulated and 105 down-regulated DEGs, of which ~33.7% (32) and ~66.3% (63) showed opposite methylation patterns (**Figure 7A**), namely up-regulated genes with down methylation level and vice versa. These results suggested that these DE genes might be regulated by methylation status alteration on CGI. CHH was the most abundant methylation within CGI regions followed by CG and CHG (**Figure 7B**).

**Figure 8:**
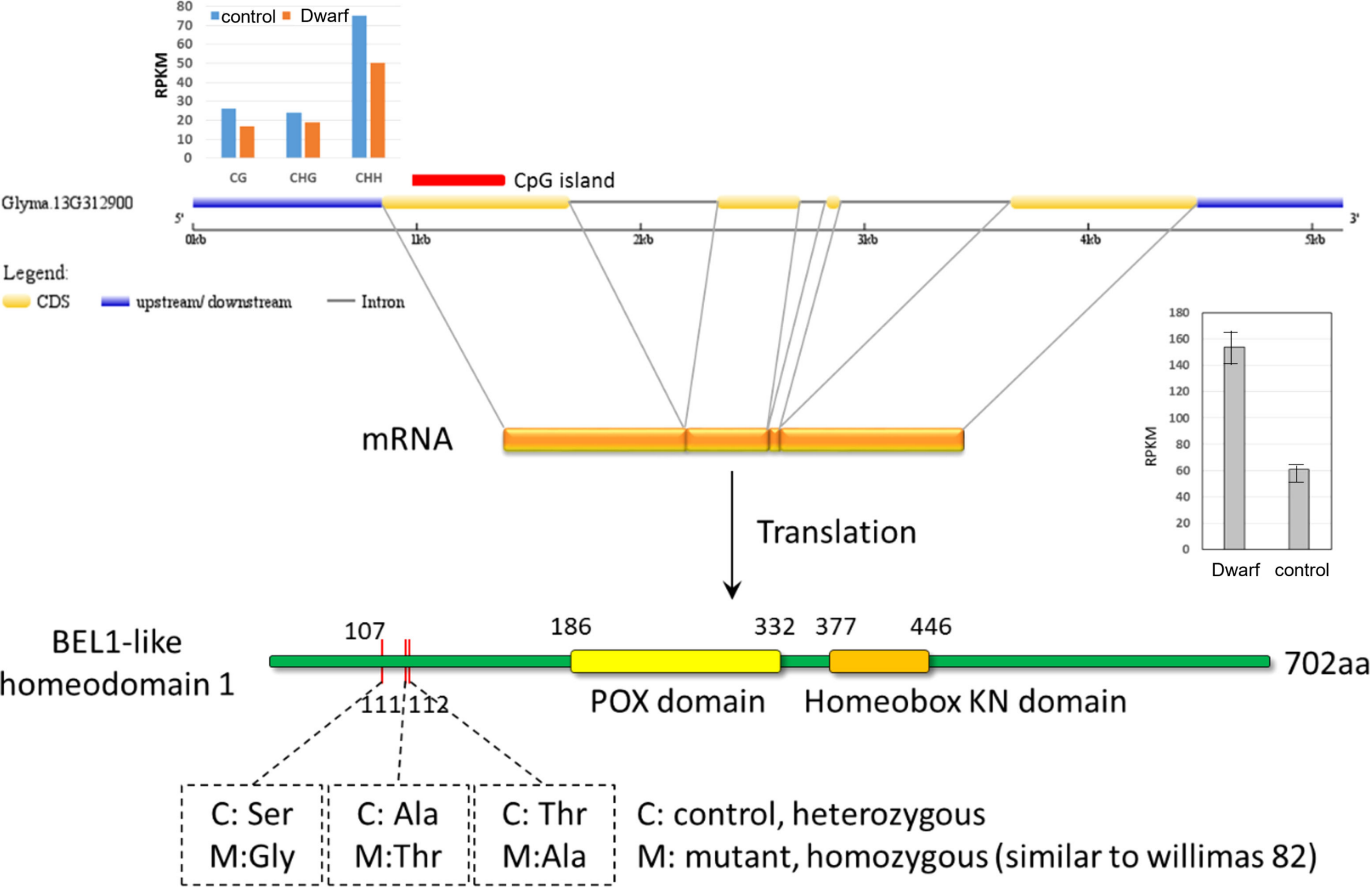
Differences of Glyma.13G312990 between dwarf mutant and control at methylation, transcriptional and genomic level. M for the dm mutant, and c for the control cultivar ZP.

Within these CGI-containing DEGs, *Glyma.13G312900*, encoding a BEL-1 protein, was modified at all three levels of transcript, methylation, and variants. BEL-1 was previously reported to interact with STM, ATH1, PNY, and PNF protein in the shoot apical meristem organogenesis and differentiation (Rutjens et al., 2010). The CGI sites in exon 1 of this gene were down methylated in all three contexts (CG, CHG, and CHH, **Figure 8**) and transcriptionally up-regulated in dwarf mutant. At the variant level, three nonsynonymous mutations (Ser107Gly, Ala111Thr, and Thr112Ala) were identified in this gene in the dwarf mutant relative to control, but they were located outside of the conserved domains of POX domain and Homebox KN domain (**Figure 8**). However, the function of BEL-1 still could be impaired. These three nonsynonymous mutations were heterozygous in control and homozygous in the dwarf mutant, which may be recessive mutations. Recessive mutations had a higher chance of loss of function than homozygous mutations. Together, these results suggested that *Glyma.13G312900* may cause the dwarf mutant phenotype and is of high priority in our future investigation.

## Discussion

Dwarfism of a plant is extensively characterized in model plants like Arabidopsis but not in soybean. Dwarfism is a preferred practical phenotype for improved yield, mechanical harvesting, etc. Artificial DNA mutation will speed up the introduction and selection of dwarf phenotypes with novel genetic variants (Holme et al., 2019). Here, the dwarf mutant *dm* was created from the EMS mutagenesis, and this mutant has an interesting dwarf phenotype. Our multiple omics characterization revealed some candidate genes or loci associated with this phenotype, which is regulated by DNA composition, gene expression and epigenetic modification. We demonstrated the multi-omics method is a helpful solution to narrow down candidates or regions for the causal mutation and regulation in soybean. Thus, the gene candidates here provide genetic clues for further understanding genetic control on dwarfism in soybean.

Genetic variants are of theoretical and applied importance in plants. Here, we revealed nucleotide differences between soybean ZP and reference genome Williams82 (Schmutz et al., 2010). A large number of SNPs and InDels suggested rich genetic differences from the reference genome Williams82. The genetic variants could serve as additional resources to the existing genetic variant dataset for breeding and scientific research in the soybean community (Valliyodan et al., 2021). The variants are mostly heterozygous, which may be responsible for the hybrid vigor in ZP. However, it causes difficulty in genetic dissection due to too many hybrid sites in one individual. The genetic purification of *dm* may also include a backcross and self-pollination.

Plant has developed a series of adaptive strategies in response to abiotic stresses. EMS mutagenesis, as a chemical or lethal stress, usually leads to single nucleotide variants and small InDels, which should be the initial cause of the dwarf phenotype. Here, the results of detected SNPs and small InDels fall into the expected types of mutations from EMS, which suggests multiple random mutations during mutagenesis are still present in an M2 plant. In response to abiotic stresses, methylome alteration exhibits variable patterns among plant species. However, these patterns are inconsistent, especially in perennial species. Here in soybean, among the 704 detected DMRs, i.e., fewer methylation sites at CG and CHG while more at CHH indicated that the EMS mutagenesis alternated methylation. Since mutation is usually deleterious and breaks the stability of the genome and gene network, the observed methylation could function for protection or repair in our mutant *dm*. DNA methylation regulates critical cellular processes in eukaryotes. Changes in DNA methylation are usually associated with transposon activation and expressional alterations during plant growth and development (Saze et al., 2012; Bennetzen and Wang, 2018). Methylation changes inhibited gene expression in most cases (424 down-regulation) as well as enhanced gene expression (263 up-regulation) in a few instances in mutant *dm*, as expected for most methylation sites in other plants (Hardcastle, 2013; Li et al., 2014; Stroud et al., 2014). Thus, methylation in mutant *dm* most likely also contributes to the dwarf phenotype. Together, this suggested that EMS mutation is not only nucleotide composition but also methylation level, in which the nucleotide composition should be the initial change while the methylation could be the derivative regulation. However, this complicated the determination of the critically causal mutation for dwarfism. Precision mapping the gene or region for this dwarf mutant still needs a series of future works, such as backcrossing the mutant to ZP several runs to remove non-causing mutations not responsible for the dwarf phenotype.

The genes identified from two omics will be the most likely candidates responsible for the mutant *dm*, especially 11 genes with both differential expressions and sequence variations, one gene with differential expression and differential methylation, three genes with differential methylation and sequence variations. The integrated effects of a mutation should finally be reflected at the gene expressional level as tested here. Our results showed that DEGs in *dm*, relative to ZP, may not have nonsynonymous mutations. Therefore, the expressional change may not directly result from mutated genes, which also complicates the screening of precision mutation for this dwarf phenotype. At the current stage, all these DEGs could be candidate genes. Previously study reported that mutation in gene *Glyma.15G157100* reduced cellulose levels and caused a dwarf phenotype (Holland et al., 2000). The cell wall related biological processes were enriched in mutant *dm*. The cellulose synthase gene *Glyma.15G157100* could be one of the key candidates, worthy of further investigation. However, in this mutant *dm*, only hemicellulose content, instead of cellulose, was 2.8% lower than that in control. Whether the phenotype is only affected by *Glyma.15G157100* could be validated in a backcross population in future works. In addition, we found that omics data analysis provides more information but still faces great challenges in interpreting the contribution to a single phenotype.

Genes in plant hormone signal pathways regulate the soybean height or dwarfism. These hormones including BR, SLs, GA and IAA, may also be involved in regulating plant height (Itoh et al., 2004; Lin et al., 2009; Hirano et al., 2010; Tong et al., 2010; Sun, 2011; Yang et al., 2011; Li et al., 2014; Zhou et al., 2015; Li et al., 2018b; Roy et al., 2021; Su et al., 2022; Zegeye et al., 2022). Our results also identified DEGs in hormone and signal transduction pathways, including *Glyma.06G063500* (KEGG ID: ARR-B, panther ID: TWO-COMPONENT RESPONSE REGULATOR ARR18), *Glyma.03G148300* (KEGG ID: GID1, panther ID: GIBBERELLIN RECEPTOR GID1B), *Glyma.14G041500* (KEGG ID: EIN3, panther ID: ETHYLENE INSENSITIVE 3-LIKE 1 PROTEIN-RELATED). These hormone related DEGs are worthy of further characterization in offspring of *dm*. Another enrichment of DEGs is the energy associated activities, e.g., ATPase. The ATPase mutation affecting energy efficiency could lead to dwarf of a plant, such as mutations in vacuolar-type H^+^-ATPases and *ala3* mutants with membrane trafficking problems (Schumacher et al., 1999; Padmanaban et al., 2004; Kumari et al., 2019; López-Marqués et al., 2020). For the downregulated expressed genes, fatty acid associated GOs were enriched, which could affect plant growth. The loss function of fatty acid desaturase 2 (FAD2) mutant displayed a dwarf phenotype (Dar et al., 2017; Lee et al., 2021). However, in another way, the fatty acid associated genes may be just at the downstreams of key causal genes such as energy associated genes. All these genes are good candidates, but their functions still need to be proved by experiments in the future.

## Conclusion

A dwarf mutant *dm* has been screened from ethyl methanesulfonate mutated seeds of the soybean cultivar Zhongpin 661. Omics analysis of whole-genome sequencing, methylation sequencing, and transcriptomic RNA-Seq revealed eleven candidate causal genes involved in cellulose, fatty acids, and energy-associated processes. The candidate genes and mutant *dm* provide theoretical and practical resources for plant-height breeding improvement.

## Data availability statement

The original contributions presented in the study are publicly available. This data can be found here: Figshare, DOI: 10.6084/m9.figshare.21571374.

## Author contributions

JS, JW, conceived and designed of this study. JS, XW, and LH conducted the data analysis, genomic comparative analysis and wrote the manuscript. ZL conceived the mutant. HR supervised the project, and reviewed the manuscript. JW revised the manuscript. All authors contributed to the article and approved the submitted version.

## Acknowledgments

This work was supported by the National Natural Scientific Foundation of China (Grant No.: 32072016). The mutant material was obtained by Prof. Lijuan Qiu, Institute of Crop Sciences, Chinese Academy of Agricultural Sciences, and its genome was sequenced.

## Conflict of interest

The authors declare that the research was conducted in the absence of any commercial or financial relationships that could be construed as a potential conflict of interest.

## Publisher’s note

All claims expressed in this article are solely those of the authors and do not necessarily represent those of their affiliated organizations, or those of the publisher, the editors and the reviewers. Any product that may be evaluated in this article, or claim that may be made by its manufacturer, is not guaranteed or endorsed by the publisher.

## Abbreviations

EMS, ethyl methanesulfonate; DEG, differentially expressed gene; CGI, CpG island.
