# Supplementary material for: Genetic dissection of the soybean dwarf mutant dm with integrated genomic, transcriptomic and methylomic analyses
Source: Front Plant Sci. 2022 Nov 21;13:1017672. doi: 10.3389/fpls.2022.1017672 (PMC9721362; doi:10.3389/fpls.2022.1017672)
Supplement: Supplementary file 1 [file DataSheet_1.docx]

STable 1. Statistics of SNPs caused genomic variation

| Compared iterms | Dwarf mutant v.s. Williams 82 | Control v.s. Williams 82 | Dwarf mutant v.s. Control |
| --- | --- | --- | --- |
| SNP number | 215132 | 224042 | 32231 |
| Transition (Ti) | 124113 | 128616 | 18956 |
| Transversion (Tv) | 91019 | 95426 | 13275 |
| Ti/Tv | ~1.36 | ~1.34 | 1.43 |
| Heterozygosity | 58924 | 62432 | 30416 |
| Homozygosity | 156208 | 161610 | 1815 |
| Heterozygosity ratio (%) | ~27.38 | ~27.86 | ~94.37 |

STable 2. SNPs in gene with non-synonymous substitution and alternative splicing mutated

| **#CHROM** | **POS** | **REF** | **ALT** | **QUAL** | **Reletive_SNP_pos_to_gene_model** | **Gene_model_with_mutation** |
| --- | --- | --- | --- | --- | --- | --- |
| Chr01 | 47747083 | A | T | 75.87 | NON_SYNONYMOUS_CODING | Glyma.01G143700 |
| Chr01 | 45836912 | G | A | 53.21 | NON_SYNONYMOUS_CODING | Glyma.01G135900 |
| Chr01 | 45837298 | A | G | 71.19 | NON_SYNONYMOUS_CODING | Glyma.01G135900 |
| Chr01 | 51545696 | T | C | 120.23 | NON_SYNONYMOUS_CODING | Glyma.01G179600 |
| Chr01 | 52992685 | G | A | 424.52 | SPLICE_SITE_ACCEPTOR | Glyma.01G195900 |
| Chr01 | 2531123 | T | C | 171.19 | NON_SYNONYMOUS_CODING | Glyma.01G024700 |
| Chr01 | 52816539 | G | A | 216.62 | NON_SYNONYMOUS_CODING | Glyma.01G193600 |
| Chr01 | 47747092 | A | G | 73.44 | NON_SYNONYMOUS_CODING | Glyma.01G143700 |
| Chr01 | 54512584 | A | T | 252.57 | STOP_GAINED | Glyma.01G214000 |
| Chr01 | 6836276 | G | A | 33.87 | STOP_GAINED | Glyma.01G053900 |
| Chr01 | 37318500 | A | T | 42.87 | STOP_LOST | Glyma.01G109400 |
| Chr01 | 56343760 | G | A | 357.2 | NON_SYNONYMOUS_CODING | Glyma.01G239200 |
| Chr01 | 54281336 | G | A | 187.54 | NON_SYNONYMOUS_CODING | Glyma.01G211300 |
| Chr01 | 52241633 | G | A | 174.72 | NON_SYNONYMOUS_CODING | Glyma.01G187200 |
| Chr01 | 38951953 | T | G | 153.87 | NON_SYNONYMOUS_CODING | Glyma.01G113600 |
| Chr01 | 53509934 | A | G | 378.52 | NON_SYNONYMOUS_CODING | Glyma.01G202300 |
| Chr01 | 45837313 | G | A | 71.19 | NON_SYNONYMOUS_CODING | Glyma.01G135900 |
| Chr01 | 51526739 | G | A | 39.87 | NON_SYNONYMOUS_CODING | Glyma.01G179300 |
| Chr01 | 56196389 | G | A | 231.28 | NON_SYNONYMOUS_CODING | Glyma.01G236600 |
| Chr01 | 39297555 | T | A | 454.2 | START_GAINED | Glyma.01G114000 |
| Chr01 | 51545062 | G | T | 33.28 | NON_SYNONYMOUS_CODING | Glyma.01G179600 |
| Chr01 | 51545525 | T | C | 58.28 | NON_SYNONYMOUS_CODING | Glyma.01G179600 |
| Chr01 | 2531093 | T | C | 171.3 | NON_SYNONYMOUS_CODING | Glyma.01G024700 |
| Chr01 | 56747091 | G | A | 103.26 | START_GAINED | Glyma.01G245100 |
| Chr01 | 45500770 | A | G | 91.37 | NON_SYNONYMOUS_CODING | Glyma.01G134100 |
| Chr01 | 53802657 | G | A | 277.49 | NON_SYNONYMOUS_CODING | Glyma.01G205500 |
| Chr01 | 51545675 | G | A | 117.87 | NON_SYNONYMOUS_CODING | Glyma.01G179600 |
| Chr01 | 51545097 | G | C | 55.71 | NON_SYNONYMOUS_CODING | Glyma.01G179600 |
| Chr01 | 38951999 | C | G | 47.44 | NON_SYNONYMOUS_CODING | Glyma.01G113600 |
| Chr01 | 54892494 | G | A | 219.57 | NON_SYNONYMOUS_CODING | Glyma.01G219200 |
| Chr01 | 11504089 | C | T | 626.19 | NON_SYNONYMOUS_CODING | Glyma.01G068600 |
| Chr01 | 38951961 | C | G | 151.02 | NON_SYNONYMOUS_CODING | Glyma.01G113600 |
| Chr01 | 2531099 | C | T | 171.3 | NON_SYNONYMOUS_CODING | Glyma.01G024700 |
| Chr01 | 3018526 | C | A | 185.21 | NON_SYNONYMOUS_CODING | Glyma.01G028700 |
| Chr01 | 53173104 | G | A | 61.45 | NON_SYNONYMOUS_CODING | Glyma.01G197800 |
| Chr01 | 53662711 | A | G | 269.49 | NON_SYNONYMOUS_CODING | Glyma.01G203400 |
| Chr01 | 2531033 | A | T | 190.71 | NON_SYNONYMOUS_CODING | Glyma.01G024700 |
| Chr01 | 45500830 | C | T | 96.23 | NON_SYNONYMOUS_CODING | Glyma.01G134100 |
| Chr01 | 49095918 | A | G | 1255.19 | NON_SYNONYMOUS_CODING | Glyma.01G153400 |
| Chr01 | 51545523 | T | C | 58.28 | NON_SYNONYMOUS_CODING | Glyma.01G179600 |
| Chr01 | 36554595 | C | T | 545.19 | NON_SYNONYMOUS_CODING | Glyma.01G107000 |
| Chr01 | 51545695 | G | C | 120.23 | STOP_GAINED | Glyma.01G179600 |
| Chr01 | 45501043 | A | C | 72.23 | NON_SYNONYMOUS_CODING | Glyma.01G134100 |
| Chr01 | 8623076 | G | A | 138.28 | NON_SYNONYMOUS_CODING | Glyma.01G062000 |
| Chr01 | 7636765 | G | A | 149.23 | NON_SYNONYMOUS_CODING | Glyma.01G057900 |
| Chr01 | 51545547 | A | G | 57.23 | NON_SYNONYMOUS_CODING | Glyma.01G179600 |
| Chr01 | 53872910 | G | T | 418.62 | NON_SYNONYMOUS_CODING | Glyma.01G206400 |
| Chr01 | 55891958 | T | C | 40.23 | NON_SYNONYMOUS_CODING | Glyma.01G232400 |
| Chr01 | 8320706 | A | T | 60.24 | NON_SYNONYMOUS_CODING | Glyma.01G060500 |
| Chr01 | 51545510 | A | T | 128.87 | NON_SYNONYMOUS_CODING | Glyma.01G179600 |
| Chr01 | 51526713 | G | A | 39.87 | NON_SYNONYMOUS_CODING | Glyma.01G179300 |
| Chr01 | 51545555 | A | G | 57.23 | NON_SYNONYMOUS_CODING | Glyma.01G179600 |
| Chr01 | 18300086 | G | A | 57.23 | NON_SYNONYMOUS_CODING | Glyma.01G076900 |
| Chr01 | 45501020 | A | G | 98.84 | NON_SYNONYMOUS_CODING | Glyma.01G134100 |
| Chr01 | 10106516 | A | G | 40.23 | NON_SYNONYMOUS_CODING | Glyma.01G065700 |
| Chr01 | 2531044 | C | T | 186.19 | NON_SYNONYMOUS_CODING | Glyma.01G024700 |
| Chr02 | 9494466 | T | C | 87.87 | NON_SYNONYMOUS_CODING | Glyma.02G100800 |
| Chr02 | 47561444 | T | A | 112.3 | NON_SYNONYMOUS_CODING | Glyma.02G298300 |
| Chr02 | 9821357 | C | G | 112.02 | NON_SYNONYMOUS_CODING | Glyma.02G103400 |
| Chr02 | 46777363 | A | T | 354.87 | NON_SYNONYMOUS_CODING | Glyma.02G287400 |
| Chr02 | 5906967 | G | C | 205.86 | NON_SYNONYMOUS_CODING | Glyma.02G066600 |
| Chr02 | 4146044 | C | A | 59.87 | NON_SYNONYMOUS_CODING | Glyma.02G044800 |
| Chr02 | 5390689 | T | A | 60.87 | NON_SYNONYMOUS_CODING | Glyma.02G059900 |
| Chr02 | 48503168 | T | A | 95.23 | NON_SYNONYMOUS_CODING | Glyma.02G311700 |
| Chr02 | 9494550 | C | A | 84.54 | NON_SYNONYMOUS_CODING | Glyma.02G100800 |
| Chr02 | 27580226 | C | T | 35.87 | SPLICE_SITE_ACCEPTOR | Glyma.02G173300 |
| Chr02 | 13785058 | C | T | 407.4 | NON_SYNONYMOUS_CODING | Glyma.02G133400 |
| Chr02 | 47561499 | C | A | 124.87 | NON_SYNONYMOUS_CODING | Glyma.02G298300 |
| Chr02 | 38740065 | A | G | 48.87 | NON_SYNONYMOUS_CODING | Glyma.02G202300 |
| Chr02 | 9494515 | C | T | 93.54 | NON_SYNONYMOUS_CODING | Glyma.02G100800 |
| Chr02 | 38740350 | G | A | 45.87 | NON_SYNONYMOUS_CODING | Glyma.02G202300 |
| Chr02 | 5390681 | C | T | 60.87 | NON_SYNONYMOUS_CODING | Glyma.02G059900 |
| Chr02 | 9494641 | T | G | 75.87 | NON_SYNONYMOUS_CODING | Glyma.02G100800 |
| Chr02 | 47561483 | A | C | 124.87 | NON_SYNONYMOUS_CODING | Glyma.02G298300 |
| Chr02 | 47322099 | C | A | 32.87 | NON_SYNONYMOUS_CODING | Glyma.02G295100 |
| Chr03 | 42581376 | G | A | 179 | NON_SYNONYMOUS_CODING | Glyma.03G223000 |
| Chr03 | 5980220 | G | A | 133.86 | NON_SYNONYMOUS_CODING | Glyma.03G047000 |
| Chr03 | 40608298 | C | G | 112.87 | NON_SYNONYMOUS_CODING | Glyma.03G196400 |
| Chr03 | 4854399 | G | A | 295.34 | NON_SYNONYMOUS_CODING | Glyma.03G039200 |
| Chr03 | 40660589 | G | A | 287.47 | NON_SYNONYMOUS_CODING | Glyma.03G197100 |
| Chr03 | 1240094 | C | T | 78.87 | NON_SYNONYMOUS_CODING | Glyma.03G012300 |
| Chr03 | 40001556 | C | T | 107.45 | NON_SYNONYMOUS_CODING | Glyma.03G189000 |
| Chr03 | 4892915 | A | G,T | 269.8 | NON_SYNONYMOUS_CODING | Glyma.03G039300 |
| Chr03 | 40626597 | T | G | 172.87 | NON_SYNONYMOUS_CODING | Glyma.03G196600 |
| Chr03 | 28881908 | G | A | 611.19 | NON_SYNONYMOUS_CODING | Glyma.03G099800 |
| Chr03 | 40608701 | G | T | 32.84 | NON_SYNONYMOUS_CODING | Glyma.03G196400 |
| Chr03 | 5980224 | G | A | 133.86 | NON_SYNONYMOUS_CODING | Glyma.03G047000 |
| Chr03 | 1240143 | T | G | 78.87 | NON_SYNONYMOUS_CODING | Glyma.03G012300 |
| Chr03 | 8001720 | A | C | 61.71 | NON_SYNONYMOUS_CODING | Glyma.03G057100 |
| Chr03 | 40607800 | C | T | 67.87 | NON_SYNONYMOUS_CODING | Glyma.03G196400 |
| Chr03 | 4608942 | G | A | 33.87 | STOP_GAINED | Glyma.03G037500 |
| Chr03 | 36398138 | G | A | 168.2 | NON_SYNONYMOUS_CODING | Glyma.03G148300 |
| Chr03 | 4794186 | C | G | 156.19 | NON_SYNONYMOUS_CODING | Glyma.03G038800 |
| Chr03 | 4892781 | C | G | 87.37 | NON_SYNONYMOUS_CODING | Glyma.03G039300 |
| Chr03 | 4855072 | A | T | 79.23 | NON_SYNONYMOUS_CODING | Glyma.03G039200 |
| Chr03 | 5979548 | G | C | 30.21 | NON_SYNONYMOUS_CODING | Glyma.03G047000 |
| Chr03 | 34973952 | G | T | 88.2 | NON_SYNONYMOUS_CODING | Glyma.03G134500 |
| Chr03 | 42983052 | T | A | 47.28 | NON_SYNONYMOUS_CODING | Glyma.03G227800 |
| Chr03 | 42233134 | C | A | 431.43 | NON_SYNONYMOUS_CODING | Glyma.03G218700 |
| Chr03 | 36473180 | G | A | 255.86 | NON_SYNONYMOUS_CODING | Glyma.03G149300 |
| Chr03 | 4793636 | C | G | 586.83 | NON_SYNONYMOUS_CODING | Glyma.03G038800 |
| Chr03 | 4596029 | T | C | 57.33 | NON_SYNONYMOUS_CODING | Glyma.03G037400 |
| Chr03 | 4519235 | A | C | 37.42 | NON_SYNONYMOUS_CODING | Glyma.03G037000 |
| Chr03 | 4609116 | C | T | 189.23 | NON_SYNONYMOUS_CODING | Glyma.03G037500 |
| Chr03 | 4894251 | A | T | 68.95 | NON_SYNONYMOUS_CODING | Glyma.03G039300 |
| Chr03 | 36629779 | G | A | 154.23 | NON_SYNONYMOUS_CODING | Glyma.03G151400 |
| Chr03 | 8001418 | A | G | 189.23 | NON_SYNONYMOUS_CODING | Glyma.03G057100 |
| Chr03 | 4200650 | C | A,T | 404.08 | STOP_GAINED | Glyma.03G034900 |
| Chr03 | 38719234 | C | T | 279.54 | NON_SYNONYMOUS_CODING | Glyma.03G173000 |
| Chr03 | 6927658 | C | G | 39.87 | NON_SYNONYMOUS_CODING | Glyma.03G052400 |
| Chr03 | 38206603 | G | A | 184.01 | NON_SYNONYMOUS_CODING | Glyma.03G167800 |
| Chr03 | 4914073 | T | A | 194.37 | NON_SYNONYMOUS_CODING | Glyma.03G039500 |
| Chr03 | 3459026 | A | G | 355.97 | NON_SYNONYMOUS_CODING | Glyma.03G030800 |
| Chr03 | 1703053 | A | G | 55.87 | NON_SYNONYMOUS_CODING | Glyma.03G016900 |
| Chr03 | 1240139 | G | A | 78.87 | NON_SYNONYMOUS_CODING | Glyma.03G012300 |
| Chr03 | 4894032 | A | G | 42.87 | NON_SYNONYMOUS_CODING | Glyma.03G039300 |
| Chr03 | 4892440 | T | C | 615.02 | NON_SYNONYMOUS_CODING | Glyma.03G039300 |
| Chr03 | 5521062 | C | G | 187.37 | NON_SYNONYMOUS_CODING | Glyma.03G043600 |
| Chr03 | 4892778 | C | A | 524.21 | NON_SYNONYMOUS_CODING | Glyma.03G039300 |
| Chr03 | 44002598 | C | T | 442.52 | NON_SYNONYMOUS_CODING | Glyma.03G241900 |
| Chr03 | 4916157 | G | A | 63.02 | NON_SYNONYMOUS_CODING | Glyma.03G039500 |
| Chr03 | 40644081 | A | T | 204.87 | NON_SYNONYMOUS_CODING | Glyma.03G196900 |
| Chr03 | 40607803 | T | C | 67.87 | NON_SYNONYMOUS_CODING | Glyma.03G196400 |
| Chr03 | 38930502 | G | A | 162.72 | NON_SYNONYMOUS_CODING | Glyma.03G176200 |
| Chr03 | 33650201 | G | A | 513.19 | NON_SYNONYMOUS_CODING | Glyma.03G123600 |
| Chr03 | 3129151 | C | A | 666.32 | NON_SYNONYMOUS_CODING | Glyma.03G028700 |
| Chr03 | 5980196 | T | A | 133.86 | NON_SYNONYMOUS_CODING | Glyma.03G047000 |
| Chr03 | 4028405 | G | A | 202.87 | NON_SYNONYMOUS_CODING | Glyma.03G034400 |
| Chr03 | 4569451 | G | T | 135.86 | NON_SYNONYMOUS_CODING | Glyma.03G037300 |
| Chr03 | 4854404 | G | A | 137.92 | NON_SYNONYMOUS_CODING | Glyma.03G039200 |
| Chr03 | 4569181 | C | G | 147.86 | NON_SYNONYMOUS_CODING | Glyma.03G037300 |
| Chr03 | 4916161 | C | G | 63.02 | NON_SYNONYMOUS_CODING | Glyma.03G039500 |
| Chr03 | 42195973 | C | T | 387.81 | NON_SYNONYMOUS_CODING | Glyma.03G218400 |
| Chr03 | 4517716 | G | A | 46.44 | NON_SYNONYMOUS_CODING | Glyma.03G037000 |
| Chr03 | 44791500 | C | T | 155.23 | NON_SYNONYMOUS_CODING | Glyma.03G252300 |
| Chr03 | 5980305 | G | A | 590.6 | NON_SYNONYMOUS_CODING | Glyma.03G047000 |
| Chr03 | 42084457 | A | T | 484.17 | STOP_GAINED | Glyma.03G216800 |
| Chr03 | 2109152 | T | C | 69.84 | NON_SYNONYMOUS_CODING | Glyma.03G020700 |
| Chr03 | 39504554 | C | T | 528.52 | NON_SYNONYMOUS_CODING | Glyma.03G183200 |
| Chr03 | 9610258 | C | T | 58.44 | NON_SYNONYMOUS_CODING | Glyma.03G062400 |
| Chr03 | 2062876 | C | T | 107.86 | NON_SYNONYMOUS_CODING | Glyma.03G020100 |
| Chr03 | 1702870 | G | A | 127.87 | NON_SYNONYMOUS_CODING | Glyma.03G016900 |
| Chr03 | 4545701 | T | G | 96.02 | NON_SYNONYMOUS_CODING | Glyma.03G037100 |
| Chr03 | 4960131 | C | A | 127.41 | NON_SYNONYMOUS_CODING | Glyma.03G039600 |
| Chr03 | 4854447 | C | T | 84.9 | NON_SYNONYMOUS_CODING | Glyma.03G039200 |
| Chr03 | 41634441 | C | T | 461.44 | NON_SYNONYMOUS_CODING | Glyma.03G209500 |
| Chr03 | 5980190 | C | A | 133.86 | NON_SYNONYMOUS_CODING | Glyma.03G047000 |
| Chr03 | 36398091 | G | A | 198.87 | NON_SYNONYMOUS_CODING | Glyma.03G148300 |
| Chr03 | 4028628 | T | A | 30.24 | NON_SYNONYMOUS_CODING | Glyma.03G034400 |
| Chr03 | 8001419 | C | T | 189.23 | NON_SYNONYMOUS_CODING | Glyma.03G057100 |
| Chr03 | 40179280 | T | A | 506.85 | NON_SYNONYMOUS_CODING | Glyma.03G191000 |
| Chr03 | 4794249 | T | A | 255.37 | NON_SYNONYMOUS_CODING | Glyma.03G038800 |
| Chr03 | 4028743 | C | A | 280.54 | NON_SYNONYMOUS_CODING | Glyma.03G034400 |
| Chr03 | 36496881 | G | A | 66.54 | NON_SYNONYMOUS_CODING | Glyma.03G149800 |
| Chr03 | 4597011 | A | G | 72.87 | NON_SYNONYMOUS_CODING | Glyma.03G037400 |
| Chr03 | 1237469 | C | T | 51.87 | NON_SYNONYMOUS_CODING | Glyma.03G012300 |
| Chr03 | 4855040 | C | T | 81.22 | NON_SYNONYMOUS_CODING | Glyma.03G039200 |
| Chr03 | 42300568 | G | A | 212.62 | NON_SYNONYMOUS_CODING | Glyma.03G219500 |
| Chr03 | 4200094 | T | G | 107.44 | NON_SYNONYMOUS_CODING | Glyma.03G034900 |
| Chr03 | 40608867 | A | G | 196.87 | NON_SYNONYMOUS_CODING | Glyma.03G196400 |
| Chr03 | 8001346 | G | A | 332.3 | NON_SYNONYMOUS_CODING | Glyma.03G057100 |
| Chr03 | 4569176 | T | C | 147.86 | NON_SYNONYMOUS_CODING | Glyma.03G037300 |
| Chr03 | 24143409 | T | G | 171.28 | NON_SYNONYMOUS_CODING | Glyma.03G084500 |
| Chr03 | 5445111 | C | A | 186.86 | NON_SYNONYMOUS_CODING | Glyma.03G043000 |
| Chr03 | 4916153 | T | A | 103.95 | NON_SYNONYMOUS_CODING | Glyma.03G039500 |
| Chr03 | 4854602 | A | G | 57.19 | NON_SYNONYMOUS_CODING | Glyma.03G039200 |
| Chr03 | 9610259 | T | A | 58.44 | NON_SYNONYMOUS_CODING | Glyma.03G062400 |
| Chr03 | 44875644 | G | A | 725.19 | NON_SYNONYMOUS_CODING | Glyma.03G253000 |
| Chr03 | 37237229 | G | A | 391.24 | NON_SYNONYMOUS_CODING | Glyma.03G157000 |
| Chr03 | 4597271 | C | A | 300.44 | NON_SYNONYMOUS_CODING | Glyma.03G037400 |
| Chr03 | 5445108 | A | T | 186.86 | NON_SYNONYMOUS_CODING | Glyma.03G043000 |
| Chr03 | 4854857 | G | T | 137.41 | NON_SYNONYMOUS_CODING | Glyma.03G039200 |
| Chr03 | 8001499 | G | A | 845.19 | NON_SYNONYMOUS_CODING | Glyma.03G057100 |
| Chr03 | 9208741 | A | T | 637.21 | NON_SYNONYMOUS_CODING | Glyma.03G061300 |
| Chr03 | 1240130 | T | C | 78.87 | NON_SYNONYMOUS_CODING | Glyma.03G012300 |
| Chr03 | 4916142 | A | C | 36.95 | NON_SYNONYMOUS_CODING | Glyma.03G039500 |
| Chr03 | 42989356 | C | T | 177.72 | NON_SYNONYMOUS_CODING | Glyma.03G227900 |
| Chr03 | 4028579 | G | C | 47.54 | NON_SYNONYMOUS_CODING | Glyma.03G034400 |
| Chr03 | 4545679 | C | A | 94.11 | NON_SYNONYMOUS_CODING | Glyma.03G037100 |
| Chr03 | 6951826 | T | G | 573.83 | NON_SYNONYMOUS_CODING | Glyma.03G052600 |
| Chr03 | 4200857 | G | A | 121.49 | NON_SYNONYMOUS_CODING | Glyma.03G034900 |
| Chr03 | 1240088 | G | A | 78.87 | STOP_GAINED | Glyma.03G012300 |
| Chr03 | 28323084 | G | A | 133.87 | STOP_GAINED | Glyma.03G096100 |
| Chr03 | 4596441 | G | A | 30.73 | NON_SYNONYMOUS_CODING | Glyma.03G037400 |
| Chr03 | 5577694 | C | G | 41.02 | NON_SYNONYMOUS_CODING | Glyma.03G044100 |
| Chr03 | 5520894 | A | G | 46.19 | NON_SYNONYMOUS_CODING | Glyma.03G043600 |
| Chr03 | 40607717 | G | T | 135.87 | NON_SYNONYMOUS_CODING | Glyma.03G196400 |
| Chr03 | 4794472 | G | C | 93.87 | NON_SYNONYMOUS_CODING | Glyma.03G038800 |
| Chr03 | 5980197 | T | C | 133.86 | NON_SYNONYMOUS_CODING | Glyma.03G047000 |
| Chr03 | 1703276 | C | T | 84.2 | NON_SYNONYMOUS_CODING | Glyma.03G016900 |
| Chr03 | 8001398 | G | A | 192.23 | NON_SYNONYMOUS_CODING | Glyma.03G057100 |
| Chr03 | 39050801 | C | T | 314.53 | NON_SYNONYMOUS_CODING | Glyma.03G177700 |
| Chr03 | 42140780 | C | T | 279.83 | NON_SYNONYMOUS_CODING | Glyma.03G217500 |
| Chr03 | 40616204 | C | G | 37.21 | NON_SYNONYMOUS_CODING | Glyma.03G196500 |
| Chr03 | 4028252 | G | A | 52.45 | NON_SYNONYMOUS_CODING | Glyma.03G034400 |
| Chr03 | 5977412 | G | A | 298.49 | NON_SYNONYMOUS_CODING | Glyma.03G047000 |
| Chr03 | 5980204 | C | G | 133.86 | NON_SYNONYMOUS_CODING | Glyma.03G047000 |
| Chr03 | 45778736 | T | C | 77.87 | NON_SYNONYMOUS_CODING | Glyma.03G264900 |
| Chr03 | 4893822 | A | T | 32.87 | NON_SYNONYMOUS_CODING | Glyma.03G039300 |
| Chr03 | 1240083 | A | C | 78.87 | SPLICE_SITE_DONOR | Glyma.03G012300 |
| Chr03 | 1702854 | C | G | 127.87 | NON_SYNONYMOUS_CODING | Glyma.03G016900 |
| Chr03 | 4854966 | G | C | 117.87 | NON_SYNONYMOUS_CODING | Glyma.03G039200 |
| Chr03 | 33759301 | G | A | 49.21 | NON_SYNONYMOUS_CODING | Glyma.03G124500 |
| Chr03 | 38419516 | G | A | 200.62 | NON_SYNONYMOUS_CODING | Glyma.03G169600 |
| Chr03 | 45778744 | A | G | 77.87 | NON_SYNONYMOUS_CODING | Glyma.03G264900 |
| Chr03 | 4854837 | A | G | 150.87 | NON_SYNONYMOUS_CODING | Glyma.03G039200 |
| Chr03 | 5575803 | G | A | 68.71 | NON_SYNONYMOUS_CODING | Glyma.03G044100 |
| Chr03 | 35804755 | G | A | 76.23 | NON_SYNONYMOUS_CODING | Glyma.03G142300 |
| Chr03 | 34973811 | G | A | 40.87 | NON_SYNONYMOUS_CODING | Glyma.03G134500 |
| Chr03 | 4794190 | C | A | 156.19 | NON_SYNONYMOUS_CODING | Glyma.03G038800 |
| Chr03 | 4854060 | G | A | 59.82 | NON_SYNONYMOUS_CODING | Glyma.03G039200 |
| Chr03 | 36398418 | G | T | 204.87 | NON_SYNONYMOUS_CODING | Glyma.03G148300 |
| Chr03 | 1240091 | C | T | 78.87 | NON_SYNONYMOUS_CODING | Glyma.03G012300 |
| Chr03 | 5519647 | G | A | 459.46 | NON_SYNONYMOUS_CODING | Glyma.03G043600 |
| Chr03 | 26543114 | C | T | 76.28 | NON_SYNONYMOUS_CODING | Glyma.03G089000 |
| Chr03 | 4200100 | G | T | 75.44 | NON_SYNONYMOUS_CODING | Glyma.03G034900 |
| Chr03 | 40170769 | G | A | 295.82 | SPLICE_SITE_ACCEPTOR | Glyma.03G190900 |
| Chr03 | 43703418 | C | T | 447.52 | NON_SYNONYMOUS_CODING | Glyma.03G237100 |
| Chr04 | 50308703 | T | A | 57.32 | NON_SYNONYMOUS_CODING | Glyma.04G234600 |
| Chr04 | 6712423 | C | T | 768.19 | NON_SYNONYMOUS_CODING | Glyma.04G079800 |
| Chr04 | 261409 | T | A | 262.23 | STOP_GAINED | Glyma.04G003100 |
| Chr04 | 15993922 | G | A | 191.73 | NON_SYNONYMOUS_CODING | Glyma.04G123600 |
| Chr04 | 41683230 | A | T | 99.19 | NON_SYNONYMOUS_CODING | Glyma.04G166100 |
| Chr04 | 3194685 | A | G | 100.87 | SPLICE_SITE_DONOR | Glyma.04G039800 |
| Chr04 | 6689211 | G | A | 554.19 | NON_SYNONYMOUS_CODING | Glyma.04G079700 |
| Chr04 | 3194233 | C | G | 39.87 | NON_SYNONYMOUS_CODING | Glyma.04G039800 |
| Chr04 | 6062040 | C | T | 309.23 | NON_SYNONYMOUS_CODING | Glyma.04G072600 |
| Chr04 | 20222682 | C | G | 49.44 | NON_SYNONYMOUS_CODING | Glyma.04G135900 |
| Chr04 | 15993872 | C | T | 144.87 | NON_SYNONYMOUS_CODING | Glyma.04G123600 |
| Chr04 | 20222681 | C | T | 49.44 | NON_SYNONYMOUS_CODING | Glyma.04G135900 |
| Chr04 | 2943574 | C | T | 481.36 | NON_SYNONYMOUS_CODING | Glyma.04G037000 |
| Chr05 | 14960985 | T | C | 265.19 | NON_SYNONYMOUS_CODING | Glyma.05G084500 |
| Chr05 | 3224907 | C | G | 189.37 | NON_SYNONYMOUS_CODING | Glyma.05G036700 |
| Chr05 | 4888047 | C | T | 394.2 | NON_SYNONYMOUS_CODING | Glyma.05G053900 |
| Chr05 | 31937938 | C | T | 39.23 | NON_SYNONYMOUS_CODING | Glyma.05G125900 |
| Chr05 | 2729242 | A | G | 162.87 | NON_SYNONYMOUS_CODING | Glyma.05G031300 |
| Chr05 | 3711833 | C | T | 406.28 | NON_SYNONYMOUS_CODING | Glyma.05G041300 |
| Chr05 | 2244705 | C | T | 530.52 | NON_SYNONYMOUS_CODING | Glyma.05G025700 |
| Chr05 | 31018310 | G | A | 130.87 | NON_SYNONYMOUS_CODING | Glyma.05G117300 |
| Chr05 | 36234300 | C | G | 161.23 | NON_SYNONYMOUS_CODING | Glyma.05G172200 |
| Chr05 | 32846451 | G | C | 75.87 | NON_SYNONYMOUS_CODING | Glyma.05G136100 |
| Chr05 | 31018346 | C | T | 162.37 | NON_SYNONYMOUS_CODING | Glyma.05G117300 |
| Chr05 | 6229525 | C | T | 958.19 | STOP_GAINED | Glyma.05G064000 |
| Chr05 | 33836131 | C | T | 61.02 | NON_SYNONYMOUS_CODING | Glyma.05G144500 |
| Chr05 | 42015698 | G | A | 78.87 | NON_SYNONYMOUS_CODING | Glyma.05G246600 |
| Chr05 | 36244218 | T | C | 96.87 | NON_SYNONYMOUS_CODING | Glyma.05G172400 |
| Chr05 | 36168406 | C | G | 150.87 | NON_SYNONYMOUS_CODING | Glyma.05G171400 |
| Chr05 | 8043490 | C | A | 96.19 | NON_SYNONYMOUS_CODING | Glyma.05G072300 |
| Chr05 | 5305804 | T | A | 113.21 | NON_SYNONYMOUS_CODING | Glyma.05G057200 |
| Chr05 | 39815669 | T | C | 66.23 | NON_SYNONYMOUS_CODING | Glyma.05G218300 |
| Chr05 | 14960998 | T | C | 272.19 | NON_SYNONYMOUS_CODING | Glyma.05G084500 |
| Chr05 | 2728548 | A | T | 54.87 | NON_SYNONYMOUS_CODING | Glyma.05G031300 |
| Chr05 | 6133365 | C | T | 123.23 | NON_SYNONYMOUS_CODING | Glyma.05G063500 |
| Chr05 | 37561203 | T | G | 36.86 | NON_SYNONYMOUS_CODING | Glyma.05G189800 |
| Chr05 | 4495990 | T | A | 87.23 | STOP_GAINED | Glyma.05G050200 |
| Chr05 | 31018335 | C | T | 130.87 | NON_SYNONYMOUS_CODING | Glyma.05G117300 |
| Chr05 | 4927248 | C | T | 420.28 | NON_SYNONYMOUS_CODING | Glyma.05G054300 |
| Chr05 | 2728538 | C | T | 54.87 | NON_SYNONYMOUS_CODING | Glyma.05G031300 |
| Chr05 | 31018331 | G | A | 130.87 | NON_SYNONYMOUS_CODING | Glyma.05G117300 |
| Chr05 | 25032367 | C | T | 586.19 | NON_SYNONYMOUS_CODING | Glyma.05G096000 |
| Chr05 | 34703808 | G | A | 57.87 | NON_SYNONYMOUS_CODING | Glyma.05G153400 |
| Chr05 | 3224944 | C | A | 174.54 | NON_SYNONYMOUS_CODING | Glyma.05G036700 |
| Chr05 | 2724384 | C | T | 243.88 | NON_SYNONYMOUS_CODING | Glyma.05G031300 |
| Chr05 | 39815608 | G | A | 63.87 | NON_SYNONYMOUS_CODING | Glyma.05G218300 |
| Chr05 | 39815611 | C | G | 63.87 | NON_SYNONYMOUS_CODING | Glyma.05G218300 |
| Chr05 | 39815660 | A | T | 66.23 | NON_SYNONYMOUS_CODING | Glyma.05G218300 |
| Chr05 | 2729286 | A | C | 180.87 | NON_SYNONYMOUS_CODING | Glyma.05G031300 |
| Chr05 | 27132368 | C | T | 210.23 | NON_SYNONYMOUS_CODING | Glyma.05G102400 |
| Chr05 | 1944055 | C | T | 88.87 | NON_SYNONYMOUS_CODING | Glyma.05G022200 |
| Chr05 | 36169028 | G | C | 35.54 | NON_SYNONYMOUS_CODING | Glyma.05G171400 |
| Chr05 | 3224934 | A | C | 174.54 | NON_SYNONYMOUS_CODING | Glyma.05G036700 |
| Chr05 | 36234087 | C | G | 143.71 | NON_SYNONYMOUS_CODING | Glyma.05G172200 |
| Chr05 | 3224889 | A | T | 189.87 | NON_SYNONYMOUS_CODING | Glyma.05G036700 |
| Chr05 | 2729279 | C | G | 180.87 | NON_SYNONYMOUS_CODING | Glyma.05G031300 |
| Chr05 | 36234322 | T | A | 63.23 | NON_SYNONYMOUS_CODING | Glyma.05G172200 |
| Chr05 | 2729266 | T | A | 168.23 | NON_SYNONYMOUS_CODING | Glyma.05G031300 |
| Chr05 | 2724327 | T | C | 243.87 | NON_SYNONYMOUS_CODING | Glyma.05G031300 |
| Chr05 | 32846454 | A | G | 75.87 | NON_SYNONYMOUS_CODING | Glyma.05G136100 |
| Chr05 | 31018329 | C | T | 130.87 | NON_SYNONYMOUS_CODING | Glyma.05G117300 |
| Chr05 | 28141531 | G | A | 241.37 | NON_SYNONYMOUS_CODING | Glyma.05G105900 |
| Chr05 | 37561206 | T | G | 37.44 | NON_SYNONYMOUS_CODING | Glyma.05G189800 |
| Chr05 | 2728568 | C | T | 75.87 | NON_SYNONYMOUS_CODING | Glyma.05G031300 |
| Chr06 | 2672455 | C | T | 353.81 | NON_SYNONYMOUS_CODING | Glyma.06G034400 |
| Chr06 | 7139932 | T | C | 43.37 | NON_SYNONYMOUS_CODING | Glyma.06G091200 |
| Chr06 | 3644542 | A | C | 126.87 | NON_SYNONYMOUS_CODING | Glyma.06G048500 |
| Chr06 | 4096016 | G | A | 245.57 | NON_SYNONYMOUS_CODING | Glyma.06G054200 |
| Chr06 | 4141067 | C | T | 235.57 | NON_SYNONYMOUS_CODING | Glyma.06G054800 |
| Chr06 | 16856514 | T | C | 261.87 | START_GAINED | Glyma.06G191700 |
| Chr06 | 2764807 | A | C | 133.87 | NON_SYNONYMOUS_CODING | Glyma.06G036000 |
| Chr06 | 42948102 | A | T | 94.37 | NON_SYNONYMOUS_CODING | Glyma.06G252800 |
| Chr06 | 16464773 | T | A | 135.87 | NON_SYNONYMOUS_CODING | Glyma.06G188700 |
| Chr06 | 8661495 | C | G | 98.23 | NON_SYNONYMOUS_CODING | Glyma.06G107700 |
| Chr06 | 3536226 | T | G | 39.23 | NON_SYNONYMOUS_CODING | Glyma.06G046800 |
| Chr06 | 26337386 | T | C | 63.21 | NON_SYNONYMOUS_CODING | Glyma.06G220600 |
| Chr06 | 48969396 | C | T | 56.23 | START_GAINED | Glyma.06G300800 |
| Chr06 | 2764306 | C | T | 102.87 | NON_SYNONYMOUS_CODING | Glyma.06G036000 |
| Chr06 | 16464779 | A | G | 133.71 | NON_SYNONYMOUS_CODING | Glyma.06G188700 |
| Chr06 | 8661493 | C | T | 98.23 | NON_SYNONYMOUS_CODING | Glyma.06G107700 |
| Chr06 | 1972541 | A | C | 98.44 | NON_SYNONYMOUS_CODING | Glyma.06G025800 |
| Chr06 | 1972515 | A | C | 70.87 | NON_SYNONYMOUS_CODING | Glyma.06G025800 |
| Chr06 | 38799182 | G | A | 30.63 | NON_SYNONYMOUS_CODING | Glyma.06G237800 |
| Chr06 | 17125216 | C | T | 321.69 | NON_SYNONYMOUS_CODING | Glyma.06G193000 |
| Chr06 | 7139926 | A | G | 43.37 | NON_SYNONYMOUS_CODING | Glyma.06G091200 |
| Chr06 | 49393513 | G | A | 286.28 | NON_SYNONYMOUS_CODING | Glyma.06G304800 |
| Chr06 | 21738123 | A | G | 56.87 | NON_SYNONYMOUS_CODING | Glyma.06G213800 |
| Chr06 | 8661551 | C | G | 63.23 | NON_SYNONYMOUS_CODING | Glyma.06G107700 |
| Chr06 | 5652825 | G | T | 79.87 | NON_SYNONYMOUS_CODING | Glyma.06G073300 |
| Chr06 | 9512248 | C | T | 69.21 | NON_SYNONYMOUS_CODING | Glyma.06G116900 |
| Chr06 | 7139992 | A | G | 52.41 | NON_SYNONYMOUS_CODING | Glyma.06G091200 |
| Chr06 | 2765443 | G | A | 45.87 | NON_SYNONYMOUS_CODING | Glyma.06G036000 |
| Chr06 | 1972696 | G | C | 55.86 | NON_SYNONYMOUS_CODING | Glyma.06G025800 |
| Chr06 | 16687560 | C | A | 129.86 | NON_SYNONYMOUS_CODING | Glyma.06G190000 |
| Chr06 | 49092384 | G | A | 378.19 | NON_SYNONYMOUS_CODING | Glyma.06G301900 |
| Chr06 | 4520129 | C | T | 35.66 | NON_SYNONYMOUS_CODING | Glyma.06G059600 |
| Chr06 | 2765435 | A | C | 42.37 | NON_SYNONYMOUS_CODING | Glyma.06G036000 |
| Chr06 | 42865565 | T | A | 348.87 | NON_SYNONYMOUS_CODING | Glyma.06G252300 |
| Chr06 | 2764277 | C | T | 102.23 | NON_SYNONYMOUS_CODING | Glyma.06G036000 |
| Chr06 | 50556435 | A | T | 391.44 | NON_SYNONYMOUS_CODING | Glyma.06G316700 |
| Chr06 | 8310587 | A | C | 136.87 | NON_SYNONYMOUS_CODING | Glyma.06G104400 |
| Chr06 | 20398877 | T | A | 42.23 | NON_SYNONYMOUS_CODING | Glyma.06G208700 |
| Chr06 | 12372365 | C | T | 187.19 | NON_SYNONYMOUS_CODING | Glyma.06G151800 |
| Chr06 | 38799185 | A | G | 30.37 | NON_SYNONYMOUS_CODING | Glyma.06G237800 |
| Chr06 | 46234513 | C | T | 87.37 | NON_SYNONYMOUS_CODING | Glyma.06G272100 |
| Chr06 | 3027629 | C | T | 462.52 | NON_SYNONYMOUS_CODING | Glyma.06G040300 |
| Chr06 | 13286670 | C | T | 57.32 | NON_SYNONYMOUS_CODING | Glyma.06G160800 |
| Chr06 | 8661446 | C | A | 33.23 | NON_SYNONYMOUS_CODING | Glyma.06G107700 |
| Chr06 | 9512258 | G | T | 69.21 | NON_SYNONYMOUS_CODING | Glyma.06G116900 |
| Chr06 | 20399659 | G | A | 93.46 | NON_SYNONYMOUS_CODING | Glyma.06G208700 |
| Chr06 | 3489478 | T | C | 212.57 | NON_SYNONYMOUS_CODING | Glyma.06G046000 |
| Chr06 | 8661501 | C | G | 98.23 | NON_SYNONYMOUS_CODING | Glyma.06G107700 |
| Chr06 | 1972380 | C | A | 49.87 | NON_SYNONYMOUS_CODING | Glyma.06G025800 |
| Chr06 | 7139627 | A | T | 33.23 | NON_SYNONYMOUS_CODING | Glyma.06G091200 |
| Chr06 | 42866042 | G | T | 80.32 | STOP_GAINED | Glyma.06G252300 |
| Chr06 | 5098541 | A | C | 42.28 | NON_SYNONYMOUS_CODING | Glyma.06G066900 |
| Chr07 | 15513374 | C | A | 264.87 | NON_SYNONYMOUS_CODING | Glyma.07G130800 |
| Chr07 | 9302617 | T | C | 468.22 | NON_SYNONYMOUS_CODING | Glyma.07G098600 |
| Chr07 | 6249249 | G | A | 88.73 | NON_SYNONYMOUS_CODING | Glyma.07G069100 |
| Chr07 | 9614173 | A | T | 351.21 | NON_SYNONYMOUS_CODING | Glyma.07G101300 |
| Chr07 | 43174341 | G | A | 63.23 | NON_SYNONYMOUS_CODING | Glyma.07G255400 |
| Chr07 | 9833178 | G | C | 78.23 | NON_SYNONYMOUS_CODING | Glyma.07G102700 |
| Chr07 | 17287413 | C | G | 483.24 | NON_SYNONYMOUS_CODING | Glyma.07G144900 |
| Chr07 | 15513181 | C | G | 215.87 | NON_SYNONYMOUS_CODING | Glyma.07G130800 |
| Chr07 | 7577564 | A | T | 72.2 | NON_SYNONYMOUS_CODING | Glyma.07G082600 |
| Chr07 | 5816280 | G | C | 152.87 | NON_SYNONYMOUS_CODING | Glyma.07G065000 |
| Chr07 | 13764329 | G | A | 69.23 | NON_SYNONYMOUS_CODING | Glyma.07G119900 |
| Chr07 | 15511904 | A | G | 89.2 | NON_SYNONYMOUS_CODING | Glyma.07G130800 |
| Chr07 | 2043398 | C | T | 134.67 | NON_SYNONYMOUS_CODING | Glyma.07G026000 |
| Chr07 | 6249271 | A | G | 96.37 | NON_SYNONYMOUS_CODING | Glyma.07G069100 |
| Chr07 | 13755361 | G | A | 298.28 | NON_SYNONYMOUS_CODING | Glyma.07G119900 |
| Chr07 | 1652053 | C | T | 79.23 | NON_SYNONYMOUS_CODING | Glyma.07G021200 |
| Chr07 | 17935894 | G | A | 66.23 | NON_SYNONYMOUS_CODING | Glyma.07G149300 |
| Chr07 | 6249278 | T | C | 96.37 | NON_SYNONYMOUS_CODING | Glyma.07G069100 |
| Chr07 | 9385271 | A | T | 344.28 | NON_SYNONYMOUS_CODING | Glyma.07G099300 |
| Chr07 | 3548928 | G | A | 124.23 | NON_SYNONYMOUS_CODING | Glyma.07G042700 |
| Chr07 | 15512074 | C | A | 54.87 | NON_SYNONYMOUS_CODING | Glyma.07G130800 |
| Chr07 | 30213476 | C | T | 189.2 | SPLICE_SITE_DONOR | Glyma.07G172700 |
| Chr07 | 17141503 | G | T | 109.87 | NON_SYNONYMOUS_CODING | Glyma.07G143900 |
| Chr07 | 6249489 | G | T | 232.33 | NON_SYNONYMOUS_CODING | Glyma.07G069100 |
| Chr07 | 15123417 | G | A | 121.28 | NON_SYNONYMOUS_CODING | Glyma.07G126600 |
| Chr07 | 9833217 | T | A | 72.87 | NON_SYNONYMOUS_CODING | Glyma.07G102700 |
| Chr07 | 20023346 | G | A | 456.19 | NON_SYNONYMOUS_CODING | Glyma.07G159100 |
| Chr07 | 4698655 | C | T | 126.87 | NON_SYNONYMOUS_CODING | Glyma.07G053900 |
| Chr07 | 9302665 | T | C | 616.24 | STOP_LOST | Glyma.07G098600 |
| Chr07 | 9833179 | T | G | 78.23 | NON_SYNONYMOUS_CODING | Glyma.07G102700 |
| Chr07 | 16336308 | A | T | 73.23 | NON_SYNONYMOUS_CODING | Glyma.07G137700 |
| Chr07 | 27510711 | G | A | 399.19 | NON_SYNONYMOUS_CODING | Glyma.07G168300 |
| Chr07 | 15512065 | A | G | 51.2 | NON_SYNONYMOUS_CODING | Glyma.07G130800 |
| Chr07 | 15513138 | G | A | 388.87 | NON_SYNONYMOUS_CODING | Glyma.07G130800 |
| Chr07 | 3538132 | G | A | 211.37 | NON_SYNONYMOUS_CODING | Glyma.07G042600 |
| Chr07 | 10009306 | A | T | 55.86 | NON_SYNONYMOUS_CODING | Glyma.07G104100 |
| Chr07 | 41884976 | T | C | 37.87 | NON_SYNONYMOUS_CODING | Glyma.07G237600 |
| Chr07 | 4698642 | G | C | 126.87 | NON_SYNONYMOUS_CODING | Glyma.07G053900 |
| Chr07 | 9833223 | T | A | 72.87 | NON_SYNONYMOUS_CODING | Glyma.07G102700 |
| Chr07 | 6246783 | C | T | 130.19 | NON_SYNONYMOUS_CODING | Glyma.07G069200 |
| Chr07 | 19077045 | G | A | 292.44 | NON_SYNONYMOUS_CODING | Glyma.07G155300 |
| Chr07 | 6246795 | C | G | 130.19 | NON_SYNONYMOUS_CODING | Glyma.07G069200 |
| Chr07 | 5863690 | G | A | 113.2 | NON_SYNONYMOUS_CODING | Glyma.07G065500 |
| Chr07 | 958208 | C | T | 231.23 | NON_SYNONYMOUS_CODING | Glyma.07G012300 |
| Chr07 | 14910860 | G | C | 39.19 | NON_SYNONYMOUS_CODING | Glyma.07G125200 |
| Chr07 | 17923292 | G | T | 93.87 | NON_SYNONYMOUS_CODING | Glyma.07G149000 |
| Chr07 | 15831750 | A | T | 231.37 | NON_SYNONYMOUS_CODING | Glyma.07G133600 |
| Chr07 | 28616573 | T | A | 51.23 | NON_SYNONYMOUS_CODING | Glyma.07G169700 |
| Chr07 | 5896916 | A | G | 111.87 | NON_SYNONYMOUS_CODING | Glyma.07G065600 |
| Chr07 | 15511484 | T | G | 102.87 | NON_SYNONYMOUS_CODING | Glyma.07G130800 |
| Chr07 | 17141597 | G | T | 204.19 | NON_SYNONYMOUS_CODING | Glyma.07G143900 |
| Chr07 | 3352180 | C | T | 470.19 | NON_SYNONYMOUS_CODING | Glyma.07G040500 |
| Chr07 | 8571475 | C | A | 123.86 | NON_SYNONYMOUS_CODING | Glyma.07G091800 |
| Chr07 | 17141564 | G | T | 207.87 | NON_SYNONYMOUS_CODING | Glyma.07G143900 |
| Chr07 | 9302659 | T | C | 616.24 | NON_SYNONYMOUS_CODING | Glyma.07G098600 |
| Chr07 | 16569180 | G | A | 123.21 | NON_SYNONYMOUS_CODING | Glyma.07G139500 |
| Chr07 | 36355482 | C | T | 307.23 | NON_SYNONYMOUS_CODING | Glyma.07G195100 |
| Chr07 | 15513153 | C | A | 225.21 | NON_SYNONYMOUS_CODING | Glyma.07G130800 |
| Chr07 | 15351254 | G | A | 146.23 | NON_SYNONYMOUS_CODING | Glyma.07G128600 |
| Chr07 | 6249248 | T | C | 88.73 | NON_SYNONYMOUS_CODING | Glyma.07G069100 |
| Chr07 | 41057027 | A | T | 31.24 | NON_SYNONYMOUS_CODING | Glyma.07G230200 |
| Chr07 | 17287407 | C | G | 483.24 | NON_SYNONYMOUS_CODING | Glyma.07G144900 |
| Chr07 | 15511471 | A | G | 105.47 | NON_SYNONYMOUS_CODING | Glyma.07G130800 |
| Chr07 | 1222138 | C | T | 252.28 | NON_SYNONYMOUS_CODING | Glyma.07G015500 |
| Chr07 | 10871788 | G | A | 88.23 | NON_SYNONYMOUS_CODING | Glyma.07G109400 |
| Chr07 | 15512063 | A | G | 51.62 | NON_SYNONYMOUS_CODING | Glyma.07G130800 |
| Chr07 | 15512434 | A | C | 306.87 | NON_SYNONYMOUS_CODING | Glyma.07G130800 |
| Chr07 | 4698639 | A | T | 126.87 | NON_SYNONYMOUS_CODING | Glyma.07G053900 |
| Chr07 | 19032402 | G | A | 47.23 | NON_SYNONYMOUS_CODING | Glyma.07G154900 |
| Chr07 | 15559292 | G | C | 79.87 | NON_SYNONYMOUS_CODING | Glyma.07G131700 |
| Chr07 | 17287521 | C | A | 567.21 | NON_SYNONYMOUS_CODING | Glyma.07G144900 |
| Chr07 | 2048584 | G | A | 30.41 | NON_SYNONYMOUS_CODING | Glyma.07G026100 |
| Chr07 | 5727063 | A | T | 114.87 | NON_SYNONYMOUS_CODING | Glyma.07G064100 |
| Chr07 | 6246791 | A | G | 130.19 | NON_SYNONYMOUS_CODING | Glyma.07G069200 |
| Chr07 | 2072160 | C | T | 242.47 | NON_SYNONYMOUS_CODING | Glyma.07G026500 |
| Chr07 | 15559641 | T | A | 52.28 | NON_SYNONYMOUS_CODING | Glyma.07G131700 |
| Chr07 | 15511955 | A | C | 318.19 | NON_SYNONYMOUS_CODING | Glyma.07G130800 |
| Chr07 | 9832279 | A | T | 78.23 | NON_SYNONYMOUS_CODING | Glyma.07G102700 |
| Chr08 | 20813837 | C | T | 323.21 | NON_SYNONYMOUS_CODING | Glyma.08G243000 |
| Chr08 | 15210844 | G | A | 128.62 | NON_SYNONYMOUS_CODING | Glyma.08G189500 |
| Chr08 | 44793358 | A | T | 49.84 | START_GAINED | Glyma.08G330200 |
| Chr08 | 20397801 | A | G | 65.62 | NON_SYNONYMOUS_CODING | Glyma.08G238900 |
| Chr08 | 30742522 | G | A | 101.44 | STOP_GAINED | Glyma.08G264900 |
| Chr08 | 31512080 | G | A | 435.19 | NON_SYNONYMOUS_CODING | Glyma.08G265300 |
| Chr08 | 6870413 | C | T | 35.87 | NON_SYNONYMOUS_CODING | Glyma.08G091400 |
| Chr08 | 37481366 | A | T | 298.23 | NON_SYNONYMOUS_CODING | Glyma.08G277900 |
| Chr08 | 33258256 | G | A | 677.19 | NON_SYNONYMOUS_CODING | Glyma.08G266300 |
| Chr08 | 44901150 | A | T | 154.23 | NON_SYNONYMOUS_CODING | Glyma.08G331400 |
| Chr08 | 7675761 | A | G | 78.19 | NON_SYNONYMOUS_CODING | Glyma.08G100100 |
| Chr08 | 6870349 | G | C | 30.24 | STOP_GAINED | Glyma.08G091400 |
| Chr08 | 142378 | T | A | 99.87 | NON_SYNONYMOUS_CODING | Glyma.08G002100 |
| Chr08 | 43718334 | T | G | 204.87 | NON_SYNONYMOUS_CODING | Glyma.08G317700 |
| Chr08 | 5635557 | T | A | 154.19 | NON_SYNONYMOUS_CODING | Glyma.08G073700 |
| Chr08 | 46589849 | T | A | 30.87 | NON_SYNONYMOUS_CODING | Glyma.08G352700 |
| Chr08 | 41217667 | G | A | 211.54 | NON_SYNONYMOUS_CODING | Glyma.08G296300 |
| Chr08 | 44762218 | A | G | 221.19 | NON_SYNONYMOUS_CODING | Glyma.08G329900 |
| Chr08 | 38661689 | G | A | 454.2 | SYNONYMOUS_STOP | Glyma.08G281900 |
| Chr08 | 28529843 | G | A | 148.23 | NON_SYNONYMOUS_CODING | Glyma.08G263200 |
| Chr08 | 15983594 | A | T | 32.41 | NON_SYNONYMOUS_CODING | Glyma.08G198000 |
| Chr08 | 41343592 | A | G | 237.23 | NON_SYNONYMOUS_CODING | Glyma.08G297200 |
| Chr08 | 15983584 | A | T | 32.41 | NON_SYNONYMOUS_CODING | Glyma.08G198000 |
| Chr08 | 4121586 | C | T | 90.23 | NON_SYNONYMOUS_CODING | Glyma.08G053300 |
| Chr08 | 46562682 | G | A | 679.83 | NON_SYNONYMOUS_CODING | Glyma.08G352600 |
| Chr08 | 40875659 | A | T | 162.54 | NON_SYNONYMOUS_CODING | Glyma.08G293700 |
| Chr08 | 3507841 | C | T | 433.44 | NON_SYNONYMOUS_CODING | Glyma.08G044300 |
| Chr08 | 15552715 | C | G | 30.87 | NON_SYNONYMOUS_CODING | Glyma.08G193200 |
| Chr08 | 43718337 | T | A | 204.87 | NON_SYNONYMOUS_CODING | Glyma.08G317700 |
| Chr08 | 18621684 | G | T | 412.87 | NON_SYNONYMOUS_CODING | Glyma.08G228400 |
| Chr08 | 4123876 | C | T | 626.19 | NON_SYNONYMOUS_CODING | Glyma.08G053300 |
| Chr08 | 41729509 | T | C | 102.23 | NON_SYNONYMOUS_CODING | Glyma.08G299200 |
| Chr09 | 409431 | C | T | 48.86 | NON_SYNONYMOUS_CODING | Glyma.09G005100 |
| Chr09 | 2263167 | G | A | 44.54 | NON_SYNONYMOUS_CODING | Glyma.09G027700 |
| Chr09 | 7997903 | C | T | 516.19 | NON_SYNONYMOUS_CODING | Glyma.09G074300 |
| Chr09 | 39627201 | A | G | 100.19 | NON_SYNONYMOUS_CODING | Glyma.09G171400 |
| Chr09 | 2254740 | T | A | 129.87 | NON_SYNONYMOUS_CODING | Glyma.09G027600 |
| Chr09 | 1989108 | G | A | 74.87 | NON_SYNONYMOUS_CODING | Glyma.09G024700 |
| Chr09 | 35515238 | G | A | 114.37 | STOP_GAINED | Glyma.09G142900 |
| Chr09 | 42889966 | C | T | 42.54 | NON_SYNONYMOUS_CODING | Glyma.09G204800 |
| Chr09 | 21195959 | G | A | 255.19 | NON_SYNONYMOUS_CODING | Glyma.09G109000 |
| Chr09 | 49559506 | A | G | 48.26 | NON_SYNONYMOUS_CODING | Glyma.09G280600 |
| Chr09 | 1989534 | T | A | 245.87 | NON_SYNONYMOUS_CODING | Glyma.09G024700 |
| Chr09 | 49819665 | T | C | 38.2 | NON_SYNONYMOUS_CODING | Glyma.09G282500 |
| Chr09 | 1990789 | G | A | 81.41 | NON_SYNONYMOUS_CODING | Glyma.09G024700 |
| Chr09 | 49559331 | A | G | 81.32 | NON_SYNONYMOUS_CODING | Glyma.09G280600 |
| Chr09 | 24727192 | G | A | 116.23 | NON_SYNONYMOUS_CODING | Glyma.09G114500 |
| Chr09 | 20314673 | C | G | 81.87 | NON_SYNONYMOUS_CODING | Glyma.09G107400 |
| Chr09 | 2254083 | T | G | 69.23 | NON_SYNONYMOUS_CODING | Glyma.09G027600 |
| Chr09 | 11938416 | G | A | 228.21 | NON_SYNONYMOUS_CODING | Glyma.09G089500 |
| Chr09 | 1989553 | A | G | 125.19 | NON_SYNONYMOUS_CODING | Glyma.09G024700 |
| Chr09 | 2263221 | C | A | 243.87 | NON_SYNONYMOUS_CODING | Glyma.09G027700 |
| Chr09 | 41310525 | C | T | 39.87 | NON_SYNONYMOUS_CODING | Glyma.09G188600 |
| Chr09 | 6618248 | G | A | 134.87 | NON_SYNONYMOUS_CODING | Glyma.09G067000 |
| Chr09 | 33897223 | C | G | 207.41 | NON_SYNONYMOUS_CODING | Glyma.09G137000 |
| Chr09 | 2254567 | A | G | 667.54 | NON_SYNONYMOUS_CODING | Glyma.09G027600 |
| Chr09 | 39610381 | T | A | 270.37 | NON_SYNONYMOUS_CODING | Glyma.09G171100 |
| Chr09 | 12223759 | T | C | 144.87 | NON_SYNONYMOUS_CODING | Glyma.09G091000 |
| Chr09 | 16564789 | C | T | 487.2 | NON_SYNONYMOUS_CODING | Glyma.09G098000 |
| Chr09 | 2263222 | A | G | 243.87 | NON_SYNONYMOUS_CODING | Glyma.09G027700 |
| Chr09 | 33896739 | G | C | 102.21 | NON_SYNONYMOUS_CODING | Glyma.09G137000 |
| Chr09 | 17246457 | C | G | 53.84 | NON_SYNONYMOUS_CODING | Glyma.09G098400 |
| Chr09 | 27963830 | G | A | 145.21 | NON_SYNONYMOUS_CODING | Glyma.09G117900 |
| Chr09 | 2254610 | C | T | 66.54 | NON_SYNONYMOUS_CODING | Glyma.09G027600 |
| Chr09 | 8406941 | G | A | 158.23 | NON_SYNONYMOUS_CODING | Glyma.09G076800 |
| Chr09 | 49559452 | T | A | 166.23 | NON_SYNONYMOUS_CODING | Glyma.09G280600 |
| Chr09 | 43240908 | G | A | 161.87 | NON_SYNONYMOUS_CODING | Glyma.09G208000 |
| Chr09 | 3084211 | G | A | 109.2 | NON_SYNONYMOUS_CODING | Glyma.09G037000 |
| Chr09 | 40521688 | G | A | 55.23 | NON_SYNONYMOUS_CODING | Glyma.09G180100 |
| Chr09 | 17246500 | C | A | 159.19 | NON_SYNONYMOUS_CODING | Glyma.09G098400 |
| Chr09 | 22128497 | T | C | 33.23 | NON_SYNONYMOUS_CODING | Glyma.09G111800 |
| Chr09 | 2263216 | G | A | 243.87 | NON_SYNONYMOUS_CODING | Glyma.09G027700 |
| Chr09 | 7056286 | C | T | 355.19 | NON_SYNONYMOUS_CODING | Glyma.09G069400 |
| Chr09 | 2254732 | C | G | 129.87 | NON_SYNONYMOUS_CODING | Glyma.09G027600 |
| Chr09 | 3654801 | C | T | 375.21 | NON_SYNONYMOUS_CODING | Glyma.09G042700 |
| Chr09 | 12469471 | A | T | 71.23 | STOP_GAINED | Glyma.09G092000 |
| Chr09 | 22128523 | A | G | 33.86 | NON_SYNONYMOUS_CODING | Glyma.09G111800 |
| Chr09 | 2254564 | C | A | 667.54 | NON_SYNONYMOUS_CODING | Glyma.09G027600 |
| Chr09 | 22128547 | T | C | 30.86 | NON_SYNONYMOUS_CODING | Glyma.09G111800 |
| Chr09 | 37849840 | G | A | 176.23 | NON_SYNONYMOUS_CODING | Glyma.09G155400 |
| Chr09 | 29612153 | T | A | 352.37 | SPLICE_SITE_ACCEPTOR | Glyma.09G122900 |
| Chr09 | 33896733 | A | G | 102.26 | NON_SYNONYMOUS_CODING | Glyma.09G137000 |
| Chr09 | 32165885 | G | A | 115.86 | NON_SYNONYMOUS_CODING | Glyma.09G128600 |
| Chr09 | 37154712 | G | A | 253.23 | NON_SYNONYMOUS_CODING | Glyma.09G150600 |
| Chr09 | 22128517 | T | C | 33.86 | NON_SYNONYMOUS_CODING | Glyma.09G111800 |
| Chr09 | 33307510 | T | C | 279.23 | NON_SYNONYMOUS_CODING | Glyma.09G133900 |
| Chr09 | 1989537 | G | A | 242.2 | NON_SYNONYMOUS_CODING | Glyma.09G024700 |
| Chr09 | 2263209 | G | C | 243.87 | NON_SYNONYMOUS_CODING | Glyma.09G027700 |
| Chr09 | 39627177 | A | G | 100.19 | NON_SYNONYMOUS_CODING | Glyma.09G171400 |
| Chr09 | 42889971 | T | C | 42.28 | NON_SYNONYMOUS_CODING | Glyma.09G204800 |
| Chr09 | 20314689 | C | G | 81.87 | NON_SYNONYMOUS_CODING | Glyma.09G107400 |
| Chr09 | 41310536 | G | A | 38.87 | NON_SYNONYMOUS_CODING | Glyma.09G188600 |
| Chr09 | 22128496 | T | A | 33.23 | NON_SYNONYMOUS_CODING | Glyma.09G111800 |
| Chr09 | 15788016 | C | T | 100.23 | NON_SYNONYMOUS_CODING | Glyma.09G097600 |
| Chr09 | 20314677 | A | G | 81.87 | NON_SYNONYMOUS_CODING | Glyma.09G107400 |
| Chr09 | 49306125 | A | T | 162.87 | NON_SYNONYMOUS_CODING | Glyma.09G277700 |
| Chr09 | 36039228 | G | C | 59.23 | NON_SYNONYMOUS_CODING | Glyma.09G145100 |
| Chr09 | 49559370 | C | A | 172.87 | NON_SYNONYMOUS_CODING | Glyma.09G280600 |
| Chr10 | 50504826 | C | T | 168.73 | NON_SYNONYMOUS_CODING | Glyma.10G284500 |
| Chr10 | 37536357 | A | T | 55.23 | SPLICE_SITE_ACCEPTOR | Glyma.10G141900 |
| Chr10 | 44729399 | A | G | 285.87 | NON_SYNONYMOUS_CODING | Glyma.10G215100 |
| Chr10 | 33780772 | A | G | 138.86 | NON_SYNONYMOUS_CODING | Glyma.10G126900 |
| Chr10 | 37536380 | T | G | 72.87 | NON_SYNONYMOUS_CODING | Glyma.10G141900 |
| Chr10 | 1809687 | T | A | 491.23 | NON_SYNONYMOUS_CODING | Glyma.10G020900 |
| Chr10 | 37534681 | C | T | 72.87 | STOP_GAINED | Glyma.10G141900 |
| Chr10 | 2043312 | A | T | 76.87 | NON_SYNONYMOUS_CODING | Glyma.10G023500 |
| Chr10 | 41545288 | G | C | 32.23 | NON_SYNONYMOUS_CODING | Glyma.10G182000 |
| Chr10 | 40489009 | A | C | 105.23 | NON_SYNONYMOUS_CODING | Glyma.10G171300 |
| Chr10 | 17202292 | T | C | 526.19 | START_GAINED | Glyma.10G098700 |
| Chr10 | 37535224 | A | T | 84.87 | NON_SYNONYMOUS_CODING | Glyma.10G141900 |
| Chr10 | 40658733 | C | T | 66.23 | START_GAINED | Glyma.10G172800 |
| Chr10 | 37536381 | C | A | 72.87 | STOP_GAINED | Glyma.10G141900 |
| Chr10 | 22554694 | A | G | 33.23 | NON_SYNONYMOUS_CODING | Glyma.10G104200 |
| Chr10 | 51218051 | G | A | 163.27 | NON_SYNONYMOUS_CODING | Glyma.10G295100 |
| Chr10 | 50504802 | C | T | 58.84 | NON_SYNONYMOUS_CODING | Glyma.10G284500 |
| Chr10 | 37534704 | A | T | 33.23 | NON_SYNONYMOUS_CODING | Glyma.10G141900 |
| Chr10 | 49334415 | A | T | 102.23 | NON_SYNONYMOUS_CODING | Glyma.10G271100 |
| Chr10 | 47155842 | C | T | 34.87 | NON_SYNONYMOUS_CODING | Glyma.10G243000 |
| Chr10 | 2114281 | G | A | 114.23 | NON_SYNONYMOUS_CODING | Glyma.10G024000 |
| Chr10 | 42098245 | C | T | 539.35 | NON_SYNONYMOUS_CODING | Glyma.10G188000 |
| Chr10 | 3600847 | C | T | 525.19 | NON_SYNONYMOUS_CODING | Glyma.10G041000 |
| Chr10 | 2043322 | T | G | 73.47 | NON_SYNONYMOUS_CODING | Glyma.10G023500 |
| Chr10 | 49323696 | C | T | 66.21 | NON_SYNONYMOUS_CODING | Glyma.10G271000 |
| Chr10 | 37534685 | A | G | 72.87 | NON_SYNONYMOUS_CODING | Glyma.10G141900 |
| Chr10 | 41545284 | T | A | 32.23 | NON_SYNONYMOUS_CODING | Glyma.10G182000 |
| Chr10 | 47156032 | G | C | 148.87 | NON_SYNONYMOUS_CODING | Glyma.10G243000 |
| Chr10 | 3343505 | A | T | 51.23 | NON_SYNONYMOUS_CODING | Glyma.10G037700 |
| Chr10 | 51071432 | C | T | 457.19 | NON_SYNONYMOUS_CODING | Glyma.10G292900 |
| Chr10 | 1641119 | C | T | 761.19 | NON_SYNONYMOUS_CODING | Glyma.10G019000 |
| Chr10 | 40489010 | C | A | 105.23 | NON_SYNONYMOUS_CODING | Glyma.10G171300 |
| Chr10 | 41545091 | G | T | 44.23 | NON_SYNONYMOUS_CODING | Glyma.10G182000 |
| Chr10 | 49316206 | A | T | 108.87 | NON_SYNONYMOUS_CODING | Glyma.10G270900 |
| Chr10 | 49316218 | A | C | 108.87 | NON_SYNONYMOUS_CODING | Glyma.10G270900 |
| Chr10 | 3343504 | A | G | 51.23 | NON_SYNONYMOUS_CODING | Glyma.10G037700 |
| Chr10 | 2762057 | G | A | 79.23 | NON_SYNONYMOUS_CODING | Glyma.10G031700 |
| Chr11 | 8492480 | G | A | 110.23 | NON_SYNONYMOUS_CODING | Glyma.11G111300 |
| Chr11 | 6997048 | C | T | 354.86 | NON_SYNONYMOUS_CODING | Glyma.11G092200 |
| Chr11 | 28357408 | G | A | 88.87 | NON_SYNONYMOUS_CODING | Glyma.11G203000 |
| Chr11 | 31184999 | C | T | 288.21 | NON_SYNONYMOUS_CODING | Glyma.11G217400 |
| Chr11 | 16168655 | T | A | 289.59 | NON_SYNONYMOUS_CODING | Glyma.11G166500 |
| Chr11 | 32849489 | A | T | 103.87 | NON_SYNONYMOUS_CODING | Glyma.11G233300 |
| Chr11 | 2754482 | C | T | 142.23 | STOP_GAINED | Glyma.11G038500 |
| Chr11 | 8019414 | C | T | 174.72 | NON_SYNONYMOUS_CODING | Glyma.11G105500 |
| Chr11 | 32619741 | G | A | 42.54 | NON_SYNONYMOUS_CODING | Glyma.11G230700 |
| Chr11 | 5096928 | G | A | 478.19 | NON_SYNONYMOUS_CODING | Glyma.11G067800 |
| Chr11 | 7466127 | A | T | 344.55 | STOP_GAINED | Glyma.11G098100 |
| Chr11 | 6669301 | T | A | 145.84 | STOP_GAINED | Glyma.11G088300 |
| Chr11 | 5659579 | G | A | 78.87 | NON_SYNONYMOUS_CODING | Glyma.11G075500 |
| Chr11 | 31904909 | T | A | 273.55 | NON_SYNONYMOUS_CODING | Glyma.11G224200 |
| Chr11 | 17261930 | A | C | 58.54 | NON_SYNONYMOUS_CODING | Glyma.11G168300 |
| Chr11 | 3347711 | G | A | 513.19 | NON_SYNONYMOUS_CODING | Glyma.11G045300 |
| Chr11 | 28357427 | C | A | 54.87 | NON_SYNONYMOUS_CODING | Glyma.11G203000 |
| Chr11 | 31770312 | G | A | 165.44 | NON_SYNONYMOUS_CODING | Glyma.11G222600 |
| Chr11 | 16168603 | T | G | 132.67 | NON_SYNONYMOUS_CODING | Glyma.11G166500 |
| Chr11 | 31560918 | G | A | 114.23 | NON_SYNONYMOUS_CODING | Glyma.11G220300 |
| Chr11 | 32619634 | A | G | 73.44 | NON_SYNONYMOUS_CODING | Glyma.11G230700 |
| Chr11 | 6414629 | G | A | 432.19 | NON_SYNONYMOUS_CODING | Glyma.11G085200 |
| Chr11 | 28357441 | G | A | 78.87 | NON_SYNONYMOUS_CODING | Glyma.11G203000 |
| Chr11 | 16168616 | T | A | 310.28 | NON_SYNONYMOUS_CODING | Glyma.11G166500 |
| Chr11 | 33434113 | G | A | 38.54 | NON_SYNONYMOUS_CODING | Glyma.11G239800 |
| Chr11 | 8143663 | G | A | 307.53 | NON_SYNONYMOUS_CODING | Glyma.11G106800 |
| Chr11 | 4602843 | G | A | 385.21 | SPLICE_SITE_DONOR | Glyma.11G061700 |
| Chr11 | 5160125 | T | C | 39.87 | NON_SYNONYMOUS_CODING | Glyma.11G068700 |
| Chr11 | 6781209 | A | T | 385.44 | NON_SYNONYMOUS_CODING | Glyma.11G089500 |
| Chr11 | 32088203 | G | A | 52.19 | START_GAINED | Glyma.11G225800 |
| Chr11 | 32849829 | C | T | 42.86 | NON_SYNONYMOUS_CODING | Glyma.11G233300 |
| Chr11 | 7344521 | G | A | 250.52 | NON_SYNONYMOUS_CODING | Glyma.11G096400 |
| Chr11 | 7882415 | G | A | 452.52 | NON_SYNONYMOUS_CODING | Glyma.11G103800 |
| Chr11 | 5367682 | G | A | 514.2 | NON_SYNONYMOUS_CODING | Glyma.11G071900 |
| Chr11 | 17266351 | C | T | 37.87 | NON_SYNONYMOUS_CODING | Glyma.11G168500 |
| Chr11 | 17266335 | G | C | 45.87 | STOP_GAINED | Glyma.11G168500 |
| Chr11 | 16168637 | G | A | 443.37 | NON_SYNONYMOUS_CODING | Glyma.11G166500 |
| Chr11 | 2766419 | C | T | 181.23 | NON_SYNONYMOUS_CODING | Glyma.11G038700 |
| Chr11 | 28357307 | G | A | 303.87 | START_GAINED | Glyma.11G203000 |
| Chr12 | 2865958 | C | T | 486.2 | NON_SYNONYMOUS_CODING | Glyma.12G039600 |
| Chr12 | 6313722 | C | T | 599.73 | NON_SYNONYMOUS_CODING | Glyma.12G080300 |
| Chr12 | 3814901 | T | G | 105.87 | NON_SYNONYMOUS_CODING | Glyma.12G053000 |
| Chr12 | 18210704 | T | A | 113.84 | NON_SYNONYMOUS_CODING | Glyma.12G142400 |
| Chr12 | 18210728 | C | T | 37.87 | NON_SYNONYMOUS_CODING | Glyma.12G142400 |
| Chr12 | 37979332 | T | G | 108.3 | NON_SYNONYMOUS_CODING | Glyma.12G220400 |
| Chr12 | 18210696 | A | C | 114.87 | NON_SYNONYMOUS_CODING | Glyma.12G142400 |
| Chr12 | 394151 | T | A | 259.28 | NON_SYNONYMOUS_CODING | Glyma.12G005000 |
| Chr12 | 6632594 | C | T | 466.19 | NON_SYNONYMOUS_CODING | Glyma.12G083300 |
| Chr12 | 37979232 | C | G | 276.87 | NON_SYNONYMOUS_CODING | Glyma.12G220400 |
| Chr12 | 20763011 | G | A | 79.87 | START_GAINED | Glyma.12G148200 |
| Chr12 | 20762052 | A | C | 56.87 | NON_SYNONYMOUS_CODING | Glyma.12G148200 |
| Chr12 | 7979907 | C | G | 142.23 | NON_SYNONYMOUS_CODING | Glyma.12G095200 |
| Chr12 | 37800998 | A | T | 79.87 | NON_SYNONYMOUS_CODING | Glyma.12G218200 |
| Chr12 | 20762044 | A | C | 56.87 | NON_SYNONYMOUS_CODING | Glyma.12G148200 |
| Chr12 | 20762610 | T | C | 389.54 | NON_SYNONYMOUS_CODING | Glyma.12G148200 |
| Chr12 | 5118137 | C | T | 58.44 | NON_SYNONYMOUS_CODING | Glyma.12G070100 |
| Chr12 | 18210154 | C | G | 134.04 | NON_SYNONYMOUS_CODING | Glyma.12G142400 |
| Chr12 | 1731598 | G | A | 328.44 | NON_SYNONYMOUS_CODING | Glyma.12G023500 |
| Chr12 | 3815075 | C | A | 92.87 | NON_SYNONYMOUS_CODING | Glyma.12G053000 |
| Chr12 | 4228014 | C | A | 86.87 | NON_SYNONYMOUS_CODING | Glyma.12G058100 |
| Chr12 | 20762594 | C | A | 392.87 | NON_SYNONYMOUS_CODING | Glyma.12G148200 |
| Chr12 | 18209985 | T | A | 99.24 | NON_SYNONYMOUS_CODING | Glyma.12G142400 |
| Chr12 | 20761571 | T | C | 209.19 | NON_SYNONYMOUS_CODING | Glyma.12G148200 |
| Chr12 | 37979331 | C | T | 108.23 | NON_SYNONYMOUS_CODING | Glyma.12G220400 |
| Chr12 | 37979326 | C | G | 108.33 | NON_SYNONYMOUS_CODING | Glyma.12G220400 |
| Chr12 | 7979789 | G | A | 132.86 | NON_SYNONYMOUS_CODING | Glyma.12G095200 |
| Chr12 | 37979346 | G | T | 111.87 | NON_SYNONYMOUS_CODING | Glyma.12G220400 |
| Chr12 | 13663155 | G | C | 35.23 | NON_SYNONYMOUS_CODING | Glyma.12G123900 |
| Chr12 | 3814852 | T | A | 195.87 | NON_SYNONYMOUS_CODING | Glyma.12G053000 |
| Chr12 | 1359516 | G | A | 133.37 | NON_SYNONYMOUS_CODING | Glyma.12G019400 |
| Chr12 | 20761554 | A | C | 132.19 | NON_SYNONYMOUS_CODING | Glyma.12G148200 |
| Chr12 | 37979226 | A | T | 276.87 | NON_SYNONYMOUS_CODING | Glyma.12G220400 |
| Chr12 | 1731638 | A | T | 226.87 | NON_SYNONYMOUS_CODING | Glyma.12G023500 |
| Chr12 | 34488113 | G | T | 36.73 | NON_SYNONYMOUS_CODING | Glyma.12G183800 |
| Chr12 | 13821528 | T | A | 299.23 | NON_SYNONYMOUS_CODING | Glyma.12G126300 |
| Chr12 | 5383409 | C | T | 252.23 | NON_SYNONYMOUS_CODING | Glyma.12G072900 |
| Chr12 | 1176074 | C | T | 282.23 | NON_SYNONYMOUS_CODING | Glyma.12G016600 |
| Chr12 | 3814860 | C | T | 195.87 | NON_SYNONYMOUS_CODING | Glyma.12G053000 |
| Chr12 | 36567213 | G | A | 71.2 | NON_SYNONYMOUS_CODING | Glyma.12G205300 |
| Chr12 | 37980554 | G | A | 41.21 | START_GAINED | Glyma.12G220400 |
| Chr12 | 5427171 | A | G | 32.19 | NON_SYNONYMOUS_CODING | Glyma.12G073300 |
| Chr12 | 20762019 | T | G | 178.87 | NON_SYNONYMOUS_CODING | Glyma.12G148200 |
| Chr12 | 2466237 | C | T | 632.19 | NON_SYNONYMOUS_CODING | Glyma.12G032600 |
| Chr12 | 34488183 | C | A | 81.19 | NON_SYNONYMOUS_CODING | Glyma.12G183800 |
| Chr12 | 5344940 | C | T | 31.45 | NON_SYNONYMOUS_CODING | Glyma.12G072500 |
| Chr12 | 20762616 | A | C | 389.54 | NON_SYNONYMOUS_CODING | Glyma.12G148200 |
| Chr12 | 9396273 | A | G | 76.87 | NON_SYNONYMOUS_CODING | Glyma.12G104300 |
| Chr13 | 30918137 | C | T | 110.87 | NON_SYNONYMOUS_CODING | Glyma.13G195600 |
| Chr13 | 30415137 | T | A | 92.21 | START_GAINED | Glyma.13G190500 |
| Chr13 | 26059227 | T | C | 69.33 | NON_SYNONYMOUS_CODING | Glyma.13G147300 |
| Chr13 | 44484101 | G | C | 285.87 | NON_SYNONYMOUS_CODING | Glyma.13G356700 |
| Chr13 | 30209368 | A | G | 186.87 | NON_SYNONYMOUS_CODING | Glyma.13G188300 |
| Chr13 | 15138804 | A | G | 60.19 | NON_SYNONYMOUS_CODING | Glyma.13G054100 |
| Chr13 | 34171368 | G | A | 54.54 | NON_SYNONYMOUS_CODING | Glyma.13G229600 |
| Chr13 | 30370317 | T | C | 117.87 | NON_SYNONYMOUS_CODING | Glyma.13G190200 |
| Chr13 | 30209170 | C | T | 69.33 | NON_SYNONYMOUS_CODING | Glyma.13G188300 |
| Chr13 | 39817187 | C | A | 135.54 | NON_SYNONYMOUS_CODING | Glyma.13G300000 |
| Chr13 | 30414844 | A | G | 53.62 | NON_SYNONYMOUS_CODING | Glyma.13G190500 |
| Chr13 | 25202475 | A | T | 71.87 | START_GAINED | Glyma.13G139400 |
| Chr13 | 30924876 | C | T | 36.23 | NON_SYNONYMOUS_CODING | Glyma.13G195700 |
| Chr13 | 30177101 | G | C | 85.95 | NON_SYNONYMOUS_CODING | Glyma.13G187900 |
| Chr13 | 30390428 | A | T | 103.95 | NON_SYNONYMOUS_CODING | Glyma.13G190300 |
| Chr13 | 15138837 | T | G | 78.19 | NON_SYNONYMOUS_CODING | Glyma.13G054100 |
| Chr13 | 30356132 | G | A | 245.2 | NON_SYNONYMOUS_CODING | Glyma.13G190000 |
| Chr13 | 17216275 | C | T | 44.87 | NON_SYNONYMOUS_CODING | Glyma.13G071700 |
| Chr13 | 30210604 | A | G | 60.87 | NON_SYNONYMOUS_CODING | Glyma.13G188300 |
| Chr13 | 30210599 | T | G | 60.87 | NON_SYNONYMOUS_CODING | Glyma.13G188300 |
| Chr13 | 22104791 | C | T | 32.23 | NON_SYNONYMOUS_CODING | Glyma.13G106700 |
| Chr13 | 30663647 | G | C | 158.56 | NON_SYNONYMOUS_CODING | Glyma.13G193300 |
| Chr13 | 30390568 | A | T | 95.3 | NON_SYNONYMOUS_CODING | Glyma.13G190300 |
| Chr13 | 30917063 | C | G | 88.87 | NON_SYNONYMOUS_CODING | Glyma.13G195600 |
| Chr13 | 30209037 | G | A | 63.19 | NON_SYNONYMOUS_CODING | Glyma.13G188300 |
| Chr13 | 17431805 | C | T | 333.87 | NON_SYNONYMOUS_CODING | Glyma.13G072800 |
| Chr13 | 44484936 | C | T | 229.71 | NON_SYNONYMOUS_CODING | Glyma.13G356700 |
| Chr13 | 30390425 | A | C | 58.95 | NON_SYNONYMOUS_CODING | Glyma.13G190300 |
| Chr13 | 15138810 | G | A | 60.19 | NON_SYNONYMOUS_CODING | Glyma.13G054100 |
| Chr13 | 29859430 | T | G,C | 270.35 | NON_SYNONYMOUS_CODING | Glyma.13G184800 |
| Chr13 | 30209029 | T | C | 63.19 | NON_SYNONYMOUS_CODING | Glyma.13G188300 |
| Chr13 | 30209179 | A | T | 69.2 | NON_SYNONYMOUS_CODING | Glyma.13G188300 |
| Chr13 | 16096150 | C | A | 62.04 | NON_SYNONYMOUS_CODING | Glyma.13G062900 |
| Chr13 | 39817167 | T | C | 135.23 | NON_SYNONYMOUS_CODING | Glyma.13G300000 |
| Chr13 | 30177098 | G | A | 85.95 | NON_SYNONYMOUS_CODING | Glyma.13G187900 |
| Chr13 | 30175889 | C | T | 53.87 | NON_SYNONYMOUS_CODING | Glyma.13G187900 |
| Chr13 | 30176912 | T | A | 549.71 | NON_SYNONYMOUS_CODING | Glyma.13G187900 |
| Chr13 | 15138783 | A | G | 60.19 | NON_SYNONYMOUS_CODING | Glyma.13G054100 |
| Chr13 | 44484063 | C | T | 58.87 | NON_SYNONYMOUS_CODING | Glyma.13G356700 |
| Chr13 | 29873968 | T | C | 108.31 | NON_SYNONYMOUS_CODING | Glyma.13G184900 |
| Chr13 | 16095536 | A | C | 202.28 | NON_SYNONYMOUS_CODING | Glyma.13G062900 |
| Chr13 | 29859473 | T | G | 430.21 | NON_SYNONYMOUS_CODING | Glyma.13G184800 |
| Chr13 | 15138845 | T | C | 78.19 | NON_SYNONYMOUS_CODING | Glyma.13G054100 |
| Chr13 | 26059185 | T | G | 144.87 | NON_SYNONYMOUS_CODING | Glyma.13G147300 |
| Chr13 | 30440190 | C | A | 284.86 | START_GAINED | Glyma.13G190900 |
| Chr13 | 30727333 | C | T | 96.86 | NON_SYNONYMOUS_CODING | Glyma.13G194100 |
| Chr13 | 30727271 | T | G | 223.44 | NON_SYNONYMOUS_CODING | Glyma.13G194100 |
| Chr13 | 30210658 | A | G | 54.2 | NON_SYNONYMOUS_CODING | Glyma.13G188300 |
| Chr13 | 30175337 | T | C | 155.87 | NON_SYNONYMOUS_CODING | Glyma.13G187900 |
| Chr13 | 21414363 | T | G | 39.37 | START_GAINED | Glyma.13G099000 |
| Chr13 | 31272217 | A | G,T | 464.08 | SPLICE_SITE_DONOR | Glyma.13G199000 |
| Chr13 | 30209032 | G | A | 63.19 | NON_SYNONYMOUS_CODING | Glyma.13G188300 |
| Chr13 | 29862011 | T | C | 282.93 | NON_SYNONYMOUS_CODING | Glyma.13G184800 |
| Chr13 | 26059135 | T | C | 81.87 | NON_SYNONYMOUS_CODING | Glyma.13G147300 |
| Chr13 | 15138798 | T | C | 60.19 | NON_SYNONYMOUS_CODING | Glyma.13G054100 |
| Chr13 | 30439885 | T | C | 73.87 | NON_SYNONYMOUS_CODING | Glyma.13G190900 |
| Chr13 | 16096371 | C | A | 64.04 | NON_SYNONYMOUS_CODING | Glyma.13G062900 |
| Chr13 | 30175193 | A | G | 72.23 | NON_SYNONYMOUS_CODING | Glyma.13G187900 |
| Chr13 | 30390438 | A | T | 58.95 | NON_SYNONYMOUS_CODING | Glyma.13G190300 |
| Chr13 | 30209035 | G | A | 63.19 | NON_SYNONYMOUS_CODING | Glyma.13G188300 |
| Chr13 | 30924919 | A | G | 76.02 | NON_SYNONYMOUS_CODING | Glyma.13G195700 |
| Chr13 | 26059164 | A | G | 66.87 | NON_SYNONYMOUS_CODING | Glyma.13G147300 |
| Chr13 | 30176859 | C | G | 58.87 | NON_SYNONYMOUS_CODING | Glyma.13G187900 |
| Chr13 | 30176880 | T | G | 51.19 | NON_SYNONYMOUS_CODING | Glyma.13G187900 |
| Chr13 | 30177102 | A | G | 85.95 | NON_SYNONYMOUS_CODING | Glyma.13G187900 |
| Chr13 | 32621451 | T | A | 35.19 | NON_SYNONYMOUS_CODING | Glyma.13G212600 |
| Chr13 | 30924886 | C | G | 36.23 | NON_SYNONYMOUS_CODING | Glyma.13G195700 |
| Chr13 | 30390566 | G | A | 91.44 | NON_SYNONYMOUS_CODING | Glyma.13G190300 |
| Chr13 | 30217814 | A | G | 192.71 | NON_SYNONYMOUS_CODING | Glyma.13G188500 |
| Chr13 | 29861157 | A | G | 140.23 | NON_SYNONYMOUS_CODING | Glyma.13G184800 |
| Chr13 | 32757982 | G | A | 76.86 | NON_SYNONYMOUS_CODING | Glyma.13G214100 |
| Chr13 | 38566909 | G | C | 46.44 | NON_SYNONYMOUS_CODING | Glyma.13G284600 |
| Chr13 | 40807193 | T | C | 36.87 | NON_SYNONYMOUS_CODING | Glyma.13G312900 |
| Chr13 | 30356614 | C | A | 172.87 | NON_SYNONYMOUS_CODING | Glyma.13G190000 |
| Chr13 | 44604934 | T | C | 122.19 | NON_SYNONYMOUS_CODING | Glyma.13G358300 |
| Chr13 | 17028452 | C | T | 528.39 | NON_SYNONYMOUS_CODING | Glyma.13G070100 |
| Chr13 | 39766628 | A | G | 84.87 | NON_SYNONYMOUS_CODING | Glyma.13G299400 |
| Chr13 | 40807190 | C | T | 36.87 | NON_SYNONYMOUS_CODING | Glyma.13G312900 |
| Chr13 | 30663608 | G | C | 41.87 | NON_SYNONYMOUS_CODING | Glyma.13G193300 |
| Chr13 | 30209034 | T | C | 63.19 | NON_SYNONYMOUS_CODING | Glyma.13G188300 |
| Chr13 | 39817164 | A | G | 135.23 | START_LOST | Glyma.13G300000 |
| Chr13 | 16096193 | T | C | 41.33 | NON_SYNONYMOUS_CODING | Glyma.13G062900 |
| Chr13 | 30664957 | A | G | 644.19 | NON_SYNONYMOUS_CODING | Glyma.13G193300 |
| Chr13 | 39817182 | A | T | 135.54 | NON_SYNONYMOUS_CODING | Glyma.13G300000 |
| Chr13 | 26059144 | G | A | 81.87 | NON_SYNONYMOUS_CODING | Glyma.13G147300 |
| Chr13 | 26059113 | G | A | 81.87 | NON_SYNONYMOUS_CODING | Glyma.13G147300 |
| Chr13 | 43541322 | A | G | 116.44 | NON_SYNONYMOUS_CODING | Glyma.13G344400 |
| Chr13 | 40807205 | C | T | 33.23 | NON_SYNONYMOUS_CODING | Glyma.13G312900 |
| Chr13 | 30390427 | T | A | 103.95 | NON_SYNONYMOUS_CODING | Glyma.13G190300 |
| Chr13 | 29861154 | A | G | 140.23 | NON_SYNONYMOUS_CODING | Glyma.13G184800 |
| Chr13 | 32728525 | C | G | 62.47 | START_GAINED | Glyma.13G213600 |
| Chr13 | 29861595 | T | G,C | 741.56 | NON_SYNONYMOUS_CODING | Glyma.13G184800 |
| Chr13 | 30210217 | T | A | 99.23 | NON_SYNONYMOUS_CODING | Glyma.13G188300 |
| Chr13 | 30176895 | C | G | 54.19 | NON_SYNONYMOUS_CODING | Glyma.13G187900 |
| Chr13 | 30414822 | A | C | 57.87 | NON_SYNONYMOUS_CODING | Glyma.13G190500 |
| Chr13 | 30208103 | T | A | 170.19 | NON_SYNONYMOUS_CODING | Glyma.13G188300 |
| Chr13 | 26059189 | T | A | 144.87 | NON_SYNONYMOUS_CODING | Glyma.13G147300 |
| Chr13 | 15138782 | C | T | 60.19 | STOP_GAINED | Glyma.13G054100 |
| Chr13 | 16095527 | A | G | 202.23 | NON_SYNONYMOUS_CODING | Glyma.13G062900 |
| Chr13 | 44604955 | G | T | 237.19 | NON_SYNONYMOUS_CODING | Glyma.13G358300 |
| Chr13 | 30177599 | G | T | 314.3 | NON_SYNONYMOUS_CODING | Glyma.13G187900 |
| Chr13 | 32754711 | G | A | 313.44 | NON_SYNONYMOUS_CODING | Glyma.13G214100 |
| Chr13 | 30664961 | T | G,A | 226.85 | NON_SYNONYMOUS_CODING | Glyma.13G193300 |
| Chr13 | 26059139 | T | A | 81.87 | NON_SYNONYMOUS_CODING | Glyma.13G147300 |
| Chr13 | 28940887 | C | A | 204.19 | START_GAINED | Glyma.13G174900 |
| Chr13 | 30727278 | G | A | 224.41 | NON_SYNONYMOUS_CODING | Glyma.13G194100 |
| Chr13 | 34980785 | C | A | 137.23 | START_GAINED | Glyma.13G239400 |
| Chr13 | 30664599 | C | A | 81.87 | NON_SYNONYMOUS_CODING | Glyma.13G193300 |
| Chr13 | 30356102 | A | G | 120.2 | NON_SYNONYMOUS_CODING | Glyma.13G190000 |
| Chr13 | 34142799 | A | G | 69.87 | NON_SYNONYMOUS_CODING | Glyma.13G229200 |
| Chr13 | 30210598 | A | G | 552.19 | NON_SYNONYMOUS_CODING | Glyma.13G188300 |
| Chr13 | 30217760 | C | T | 235.2 | NON_SYNONYMOUS_CODING | Glyma.13G188500 |
| Chr13 | 30664615 | G | A | 81.87 | NON_SYNONYMOUS_CODING | Glyma.13G193300 |
| Chr13 | 30664969 | C | T | 644.19 | NON_SYNONYMOUS_CODING | Glyma.13G193300 |
| Chr13 | 43178158 | A | T | 100.47 | NON_SYNONYMOUS_CODING | Glyma.13G339800 |
| Chr13 | 30175256 | A | G | 39.2 | NON_SYNONYMOUS_CODING | Glyma.13G187900 |
| Chr13 | 26059122 | T | C | 81.87 | NON_SYNONYMOUS_CODING | Glyma.13G147300 |
| Chr13 | 30209044 | T | A | 162.87 | NON_SYNONYMOUS_CODING | Glyma.13G188300 |
| Chr13 | 30390573 | G | A | 95.3 | NON_SYNONYMOUS_CODING | Glyma.13G190300 |
| Chr13 | 29862012 | T | C | 282.93 | NON_SYNONYMOUS_CODING | Glyma.13G184800 |
| Chr13 | 30853245 | C | G | 84.87 | NON_SYNONYMOUS_CODING | Glyma.13G195100 |
| Chr13 | 33685499 | T | A | 36.44 | NON_SYNONYMOUS_CODING | Glyma.13G224000 |
| Chr13 | 30355673 | C | G | 230.89 | NON_SYNONYMOUS_CODING | Glyma.13G190000 |
| Chr13 | 29862044 | C | A | 326.85 | NON_SYNONYMOUS_CODING | Glyma.13G184800 |
| Chr13 | 30217769 | T | C | 235.2 | NON_SYNONYMOUS_CODING | Glyma.13G188500 |
| Chr13 | 30665053 | A | G | 40.87 | NON_SYNONYMOUS_CODING | Glyma.13G193300 |
| Chr13 | 30402314 | G | T | 138.55 | NON_SYNONYMOUS_CODING | Glyma.13G190400 |
| Chr13 | 30209574 | G | A | 152.24 | NON_SYNONYMOUS_CODING | Glyma.13G188300 |
| Chr13 | 30175493 | C | A | 32.87 | NON_SYNONYMOUS_CODING | Glyma.13G187900 |
| Chr13 | 16095537 | T | C | 202.28 | NON_SYNONYMOUS_CODING | Glyma.13G062900 |
| Chr13 | 30391714 | T | G | 127.87 | NON_SYNONYMOUS_CODING | Glyma.13G190300 |
| Chr13 | 29861620 | C | A | 929.19 | NON_SYNONYMOUS_CODING | Glyma.13G184800 |
| Chr13 | 34420706 | C | G | 30.87 | NON_SYNONYMOUS_CODING | Glyma.13G233400 |
| Chr13 | 29861145 | G | T | 140.23 | NON_SYNONYMOUS_CODING | Glyma.13G184800 |
| Chr13 | 44484047 | C | A | 55.37 | NON_SYNONYMOUS_CODING | Glyma.13G356700 |
| Chr13 | 30207385 | T | G | 98.24 | NON_SYNONYMOUS_CODING | Glyma.13G188300 |
| Chr13 | 30176865 | T | A | 58.87 | NON_SYNONYMOUS_CODING | Glyma.13G187900 |
| Chr13 | 26059007 | C | G | 72.22 | NON_SYNONYMOUS_CODING | Glyma.13G147300 |
| Chr13 | 30208006 | C | G | 133.87 | NON_SYNONYMOUS_CODING | Glyma.13G188300 |
| Chr13 | 30209175 | G | C | 69.2 | NON_SYNONYMOUS_CODING | Glyma.13G188300 |
| Chr13 | 39815377 | G | C | 44.86 | NON_SYNONYMOUS_CODING | Glyma.13G300000 |
| Chr13 | 30664606 | G | A | 81.87 | NON_SYNONYMOUS_CODING | Glyma.13G193300 |
| Chr13 | 44484068 | G | A | 58.87 | NON_SYNONYMOUS_CODING | Glyma.13G356700 |
| Chr13 | 26059183 | A | C | 144.87 | NON_SYNONYMOUS_CODING | Glyma.13G147300 |
| Chr13 | 30177613 | G | T | 300.34 | NON_SYNONYMOUS_CODING | Glyma.13G187900 |
| Chr13 | 30664967 | C | A | 644.19 | NON_SYNONYMOUS_CODING | Glyma.13G193300 |
| Chr13 | 26059200 | G | T | 144.87 | NON_SYNONYMOUS_CODING | Glyma.13G147300 |
| Chr13 | 43178157 | T | C | 100.47 | NON_SYNONYMOUS_CODING | Glyma.13G339800 |
| Chr14 | 45407576 | C | T | 136.23 | NON_SYNONYMOUS_CODING | Glyma.14G189300 |
| Chr14 | 4351877 | G | C | 54.23 | NON_SYNONYMOUS_CODING | Glyma.14G054500 |
| Chr14 | 27627807 | T | G | 52.87 | NON_SYNONYMOUS_CODING | Glyma.14G140800 |
| Chr14 | 39033154 | G | A | 336.23 | NON_SYNONYMOUS_CODING | Glyma.14G162300 |
| Chr14 | 46436325 | A | C | 57.21 | NON_SYNONYMOUS_CODING | Glyma.14G199400 |
| Chr14 | 10749593 | C | T | 606.19 | SPLICE_SITE_ACCEPTOR | Glyma.14G104800 |
| Chr14 | 1336743 | C | T | 362.2 | START_LOST | Glyma.14G018500 |
| Chr14 | 45103744 | A | G | 144.19 | NON_SYNONYMOUS_CODING | Glyma.14G186600 |
| Chr14 | 45884846 | C | T | 242.83 | NON_SYNONYMOUS_CODING | Glyma.14G193900 |
| Chr14 | 4351853 | A | G | 54.23 | NON_SYNONYMOUS_CODING | Glyma.14G054500 |
| Chr14 | 20325427 | T | A | 35.88 | STOP_GAINED | Glyma.14G127000 |
| Chr14 | 728372 | C | T | 392.21 | NON_SYNONYMOUS_CODING | Glyma.14G009300 |
| Chr14 | 12310997 | C | T | 120.87 | NON_SYNONYMOUS_CODING | Glyma.14G109800 |
| Chr14 | 46436340 | G | A | 57.21 | NON_SYNONYMOUS_CODING | Glyma.14G199400 |
| Chr14 | 42619370 | G | A | 113.87 | NON_SYNONYMOUS_CODING | Glyma.14G172200 |
| Chr14 | 4351892 | G | A | 102.87 | NON_SYNONYMOUS_CODING | Glyma.14G054500 |
| Chr14 | 4351843 | T | A | 54.23 | NON_SYNONYMOUS_CODING | Glyma.14G054500 |
| Chr14 | 45103734 | G | C | 144.19 | NON_SYNONYMOUS_CODING | Glyma.14G186600 |
| Chr14 | 46447052 | G | C | 125.23 | START_LOST | Glyma.14G199500 |
| Chr14 | 7081937 | C | A | 550.19 | NON_SYNONYMOUS_CODING | Glyma.14G081900 |
| Chr14 | 45296745 | C | T | 79.23 | NON_SYNONYMOUS_CODING | Glyma.14G188200 |
| Chr14 | 46436328 | A | C | 57.21 | NON_SYNONYMOUS_CODING | Glyma.14G199400 |
| Chr14 | 10711007 | T | C | 274.44 | NON_SYNONYMOUS_CODING | Glyma.14G104700 |
| Chr14 | 45588376 | G | A | 178.62 | NON_SYNONYMOUS_CODING | Glyma.14G191100 |
| Chr14 | 46688957 | C | T | 418.4 | NON_SYNONYMOUS_CODING | Glyma.14G201500 |
| Chr14 | 42619318 | G | T | 351.87 | NON_SYNONYMOUS_CODING | Glyma.14G172200 |
| Chr14 | 45893523 | C | T | 381.52 | NON_SYNONYMOUS_CODING | Glyma.14G194000 |
| Chr14 | 4351832 | G | T | 54.23 | NON_SYNONYMOUS_CODING | Glyma.14G054500 |
| Chr14 | 46436316 | A | G | 57.21 | NON_SYNONYMOUS_CODING | Glyma.14G199400 |
| Chr14 | 44810582 | C | T | 144.87 | NON_SYNONYMOUS_CODING | Glyma.14G184700 |
| Chr14 | 4351854 | C | G | 54.23 | STOP_GAINED | Glyma.14G054500 |
| Chr14 | 45735031 | G | A | 280.53 | NON_SYNONYMOUS_CODING | Glyma.14G192800 |
| Chr14 | 46436607 | T | C | 78.87 | NON_SYNONYMOUS_CODING | Glyma.14G199400 |
| Chr14 | 3676267 | C | T | 102.87 | NON_SYNONYMOUS_CODING | Glyma.14G047900 |
| Chr14 | 47548856 | A | G | 42.23 | NON_SYNONYMOUS_CODING | Glyma.14G210400 |
| Chr14 | 6524649 | T | G | 45.89 | NON_SYNONYMOUS_CODING | Glyma.14G077400 |
| Chr14 | 4351846 | G | A | 54.23 | NON_SYNONYMOUS_CODING | Glyma.14G054500 |
| Chr14 | 47666461 | T | A | 255.47 | NON_SYNONYMOUS_CODING | Glyma.14G211800 |
| Chr14 | 45103728 | C | T | 144.19 | NON_SYNONYMOUS_CODING | Glyma.14G186600 |
| Chr14 | 1173206 | C | T | 771.83 | NON_SYNONYMOUS_CODING | Glyma.14G016300 |
| Chr14 | 46436337 | G | A | 57.21 | NON_SYNONYMOUS_CODING | Glyma.14G199400 |
| Chr14 | 1832979 | T | A | 569.19 | NON_SYNONYMOUS_CODING | Glyma.14G025600 |
| Chr14 | 45103749 | G | C | 144.19 | NON_SYNONYMOUS_CODING | Glyma.14G186600 |
| Chr14 | 6089502 | C | T | 98.28 | SPLICE_SITE_ACCEPTOR | Glyma.14G072400 |
| Chr14 | 48493911 | C | T | 610.19 | NON_SYNONYMOUS_CODING | Glyma.14G219900 |
| Chr14 | 488329 | C | T | 52.28 | NON_SYNONYMOUS_CODING | Glyma.14G006100 |
| Chr14 | 4351944 | G | C | 63.21 | NON_SYNONYMOUS_CODING | Glyma.14G054500 |
| Chr14 | 1553077 | C | T | 219.86 | NON_SYNONYMOUS_CODING | Glyma.14G021900 |
| Chr14 | 45103710 | T | C | 144.19 | NON_SYNONYMOUS_CODING | Glyma.14G186600 |
| Chr14 | 4351906 | A | T | 102.87 | NON_SYNONYMOUS_CODING | Glyma.14G054500 |
| Chr14 | 46436307 | C | G | 57.21 | NON_SYNONYMOUS_CODING | Glyma.14G199400 |
| Chr14 | 46436339 | A | C | 57.21 | NON_SYNONYMOUS_CODING | Glyma.14G199400 |
| Chr14 | 12310353 | C | G | 61.23 | NON_SYNONYMOUS_CODING | Glyma.14G109800 |
| Chr14 | 46436336 | G | T | 57.21 | NON_SYNONYMOUS_CODING | Glyma.14G199400 |
| Chr14 | 4351912 | T | G | 102.87 | NON_SYNONYMOUS_CODING | Glyma.14G054500 |
| Chr15 | 7677620 | G | A | 85.23 | NON_SYNONYMOUS_CODING | Glyma.15G098700 |
| Chr15 | 6500474 | T | A | 162.87 | NON_SYNONYMOUS_CODING | Glyma.15G084700 |
| Chr15 | 6758890 | A | G | 48.28 | NON_SYNONYMOUS_CODING | Glyma.15G088000 |
| Chr15 | 33698169 | G | A | 34.23 | NON_SYNONYMOUS_CODING | Glyma.15G213400 |
| Chr15 | 43747860 | G | C | 45.87 | START_GAINED | Glyma.15G232800 |
| Chr15 | 9515044 | G | A | 63.54 | NON_SYNONYMOUS_CODING | Glyma.15G120300 |
| Chr15 | 6147647 | G | A | 214.62 | STOP_GAINED | Glyma.15G080000 |
| Chr15 | 12274564 | G | A | 361.37 | SPLICE_SITE_DONOR | Glyma.15G149000 |
| Chr15 | 43523562 | C | T | 32.25 | NON_SYNONYMOUS_CODING | Glyma.15G231500 |
| Chr15 | 9514893 | C | T | 132.21 | STOP_GAINED | Glyma.15G120300 |
| Chr15 | 9055950 | G | A | 445.19 | NON_SYNONYMOUS_CODING | Glyma.15G115100 |
| Chr15 | 48619408 | T | C | 160.73 | NON_SYNONYMOUS_CODING | Glyma.15G256800 |
| Chr15 | 43921846 | A | T | 45.23 | NON_SYNONYMOUS_CODING | Glyma.15G233400 |
| Chr15 | 6985168 | G | A | 542.52 | NON_SYNONYMOUS_CODING | Glyma.15G091000 |
| Chr15 | 6745968 | G | A | 623.19 | NON_SYNONYMOUS_CODING | Glyma.15G087600 |
| Chr15 | 43522206 | A | G | 94.87 | START_GAINED | Glyma.15G231500 |
| Chr15 | 43744587 | G | C | 111.47 | NON_SYNONYMOUS_CODING | Glyma.15G232800 |
| Chr15 | 2924804 | G | A | 305.43 | NON_SYNONYMOUS_CODING | Glyma.15G037100 |
| Chr15 | 48736902 | C | T | 69.28 | NON_SYNONYMOUS_CODING | Glyma.15G257700 |
| Chr15 | 10726048 | G | C | 30.86 | NON_SYNONYMOUS_CODING | Glyma.15G133400 |
| Chr15 | 46974274 | G | T | 110.32 | NON_SYNONYMOUS_CODING | Glyma.15G246800 |
| Chr15 | 9515052 | G | T | 63.54 | NON_SYNONYMOUS_CODING | Glyma.15G120300 |
| Chr15 | 11019801 | G | A | 524.39 | NON_SYNONYMOUS_CODING | Glyma.15G136300 |
| Chr15 | 43799181 | T | A | 96.2 | NON_SYNONYMOUS_CODING | Glyma.15G233100 |
| Chr15 | 6500458 | A | G | 162.87 | NON_SYNONYMOUS_CODING | Glyma.15G084700 |
| Chr15 | 6500468 | A | C | 162.87 | NON_SYNONYMOUS_CODING | Glyma.15G084700 |
| Chr15 | 33697887 | A | T | 296.33 | NON_SYNONYMOUS_CODING | Glyma.15G213400 |
| Chr15 | 6501077 | C | G | 101.87 | NON_SYNONYMOUS_CODING | Glyma.15G084700 |
| Chr15 | 2066269 | A | G | 196.87 | NON_SYNONYMOUS_CODING | Glyma.15G025500 |
| Chr15 | 10726183 | C | G | 70.71 | NON_SYNONYMOUS_CODING | Glyma.15G133400 |
| Chr15 | 10726062 | T | G | 109.44 | NON_SYNONYMOUS_CODING | Glyma.15G133400 |
| Chr15 | 8883398 | G | A | 485.23 | NON_SYNONYMOUS_CODING | Glyma.15G112700 |
| Chr15 | 4615844 | C | T | 55.86 | NON_SYNONYMOUS_CODING | Glyma.15G059700 |
| Chr15 | 43744581 | A | G | 111.62 | NON_SYNONYMOUS_CODING | Glyma.15G232800 |
| Chr15 | 20116508 | G | A | 141.87 | NON_SYNONYMOUS_CODING | Glyma.15G189600 |
| Chr15 | 2249378 | C | T | 412.52 | NON_SYNONYMOUS_CODING | Glyma.15G028000 |
| Chr15 | 43747560 | T | C | 59.04 | NON_SYNONYMOUS_CODING | Glyma.15G232800 |
| Chr15 | 5659732 | G | A | 67.95 | NON_SYNONYMOUS_CODING | Glyma.15G074000 |
| Chr15 | 43522311 | C | G | 38.87 | START_GAINED | Glyma.15G231500 |
| Chr15 | 43921825 | G | C | 34.23 | NON_SYNONYMOUS_CODING | Glyma.15G233400 |
| Chr15 | 1531900 | C | T | 322.49 | SPLICE_SITE_DONOR | Glyma.15G019600 |
| Chr15 | 10726070 | C | T | 110.41 | NON_SYNONYMOUS_CODING | Glyma.15G133400 |
| Chr15 | 6366266 | G | A | 416.47 | NON_SYNONYMOUS_CODING | Glyma.15G083300 |
| Chr15 | 46974143 | C | T | 239.04 | NON_SYNONYMOUS_CODING | Glyma.15G246800 |
| Chr15 | 49595920 | G | A | 35.86 | NON_SYNONYMOUS_CODING | Glyma.15G263100 |
| Chr15 | 48736905 | C | G | 69.28 | NON_SYNONYMOUS_CODING | Glyma.15G257700 |
| Chr15 | 44349304 | T | A | 190.23 | NON_SYNONYMOUS_CODING | Glyma.15G235900 |
| Chr15 | 7179432 | G | A | 66.86 | NON_SYNONYMOUS_CODING | Glyma.15G092600 |
| Chr15 | 29327950 | T | A | 66.22 | STOP_GAINED | Glyma.15G206600 |
| Chr15 | 4255915 | G | A | 346.81 | START_GAINED | Glyma.15G054100 |
| Chr15 | 48619409 | C | A | 160.73 | NON_SYNONYMOUS_CODING | Glyma.15G256800 |
| Chr15 | 43747238 | T | C | 114.87 | NON_SYNONYMOUS_CODING | Glyma.15G232800 |
| Chr15 | 6500482 | A | G | 162.87 | NON_SYNONYMOUS_CODING | Glyma.15G084700 |
| Chr15 | 13185263 | G | A | 205.23 | NON_SYNONYMOUS_CODING | Glyma.15G157100 |
| Chr15 | 43922102 | A | T | 63.87 | NON_SYNONYMOUS_CODING | Glyma.15G233400 |
| Chr15 | 43799179 | C | T | 96.2 | NON_SYNONYMOUS_CODING | Glyma.15G233100 |
| Chr15 | 2066260 | C | G | 193.23 | NON_SYNONYMOUS_CODING | Glyma.15G025500 |
| Chr15 | 43744655 | C | T | 66.23 | NON_SYNONYMOUS_CODING | Glyma.15G232800 |
| Chr15 | 10726061 | G | A | 109.44 | NON_SYNONYMOUS_CODING | Glyma.15G133400 |
| Chr15 | 43744643 | C | T | 66.23 | NON_SYNONYMOUS_CODING | Glyma.15G232800 |
| Chr15 | 6759638 | C | T | 54.28 | NON_SYNONYMOUS_CODING | Glyma.15G088000 |
| Chr15 | 5631368 | A | T | 351.52 | NON_SYNONYMOUS_CODING | Glyma.15G073600 |
| Chr15 | 49890853 | A | C | 181.44 | NON_SYNONYMOUS_CODING | Glyma.15G264900 |
| Chr15 | 48619404 | T | C | 160.73 | NON_SYNONYMOUS_CODING | Glyma.15G256800 |
| Chr15 | 4779212 | C | T | 379.52 | STOP_GAINED | Glyma.15G062100 |
| Chr15 | 7179451 | C | T | 35.86 | NON_SYNONYMOUS_CODING | Glyma.15G092600 |
| Chr15 | 13216424 | G | A | 381.23 | NON_SYNONYMOUS_CODING | Glyma.15G157700 |
| Chr15 | 6229276 | G | A | 331.52 | NON_SYNONYMOUS_CODING | Glyma.15G081500 |
| Chr15 | 10726065 | A | G | 110.04 | NON_SYNONYMOUS_CODING | Glyma.15G133400 |
| Chr15 | 6608111 | T | C | 84.87 | NON_SYNONYMOUS_CODING | Glyma.15G086100 |
| Chr15 | 6758894 | T | A | 48.73 | NON_SYNONYMOUS_CODING | Glyma.15G088000 |
| Chr15 | 4615624 | A | C | 93.89 | NON_SYNONYMOUS_CODING | Glyma.15G059700 |
| Chr15 | 41535923 | A | G | 57.87 | NON_SYNONYMOUS_CODING | Glyma.15G226000 |
| Chr15 | 20116433 | G | A | 30.44 | NON_SYNONYMOUS_CODING | Glyma.15G189600 |
| Chr15 | 7179427 | C | T | 66.86 | NON_SYNONYMOUS_CODING | Glyma.15G092600 |
| Chr16 | 32158831 | T | C | 201.87 | NON_SYNONYMOUS_CODING | Glyma.16G162700 |
| Chr16 | 34346979 | C | T | 35.19 | NON_SYNONYMOUS_CODING | Glyma.16G182500 |
| Chr16 | 34589183 | G | C | 73.71 | NON_SYNONYMOUS_CODING | Glyma.16G184000 |
| Chr16 | 2995006 | T | C | 132.23 | NON_SYNONYMOUS_CODING | Glyma.16G031700 |
| Chr16 | 36995631 | T | C | 456.52 | NON_SYNONYMOUS_CODING | Glyma.16G210900 |
| Chr16 | 32159352 | A | C | 210.54 | NON_SYNONYMOUS_CODING | Glyma.16G162700 |
| Chr16 | 5841060 | G | A | 172.23 | STOP_GAINED | Glyma.16G059800 |
| Chr16 | 33335609 | C | T | 272.23 | NON_SYNONYMOUS_CODING | Glyma.16G172600 |
| Chr16 | 32158839 | C | T | 201.87 | NON_SYNONYMOUS_CODING | Glyma.16G162700 |
| Chr16 | 35682185 | C | T | 345.79 | NON_SYNONYMOUS_CODING | Glyma.16G194800 |
| Chr16 | 34802070 | T | A | 259.54 | NON_SYNONYMOUS_CODING | Glyma.16G186400 |
| Chr16 | 33626924 | T | C | 120.73 | NON_SYNONYMOUS_CODING | Glyma.16G175100 |
| Chr16 | 35407002 | G | A | 45.23 | NON_SYNONYMOUS_CODING | Glyma.16G191700 |
| Chr16 | 32159262 | A | G | 396.86 | NON_SYNONYMOUS_CODING | Glyma.16G162700 |
| Chr16 | 33627335 | G | A | 61.87 | NON_SYNONYMOUS_CODING | Glyma.16G175100 |
| Chr16 | 35416374 | C | G | 747.21 | NON_SYNONYMOUS_CODING | Glyma.16G191800 |
| Chr16 | 10017212 | A | T | 611.19 | NON_SYNONYMOUS_CODING | Glyma.16G085300 |
| Chr16 | 1400376 | C | T | 649.19 | NON_SYNONYMOUS_CODING | Glyma.16G016000 |
| Chr16 | 2995000 | T | G | 132.23 | NON_SYNONYMOUS_CODING | Glyma.16G031700 |
| Chr16 | 33784646 | C | A | 57.87 | NON_SYNONYMOUS_CODING | Glyma.16G176600 |
| Chr16 | 31480894 | G | A | 48.87 | NON_SYNONYMOUS_CODING | Glyma.16G154400 |
| Chr16 | 37101286 | G | A | 253.67 | NON_SYNONYMOUS_CODING | Glyma.16G212800 |
| Chr16 | 36472062 | T | C | 39.87 | NON_SYNONYMOUS_CODING | Glyma.16G203800 |
| Chr16 | 32159359 | C | T | 210.54 | NON_SYNONYMOUS_CODING | Glyma.16G162700 |
| Chr16 | 33627033 | A | C | 40.21 | NON_SYNONYMOUS_CODING | Glyma.16G175100 |
| Chr16 | 35515875 | A | C | 169.44 | NON_SYNONYMOUS_CODING | Glyma.16G193000 |
| Chr16 | 16726149 | C | A | 40.87 | NON_SYNONYMOUS_CODING | Glyma.16G094900 |
| Chr16 | 37071576 | A | T | 172.72 | NON_SYNONYMOUS_CODING | Glyma.16G212400 |
| Chr16 | 12040546 | G | C | 135.23 | NON_SYNONYMOUS_CODING | Glyma.16G089600 |
| Chr16 | 10017230 | C | G | 611.19 | NON_SYNONYMOUS_CODING | Glyma.16G085300 |
| Chr16 | 32159255 | A | C | 396.86 | NON_SYNONYMOUS_CODING | Glyma.16G162700 |
| Chr16 | 34589173 | G | T | 75.87 | NON_SYNONYMOUS_CODING | Glyma.16G184000 |
| Chr16 | 33162410 | C | A | 96.87 | NON_SYNONYMOUS_CODING | Glyma.16G171200 |
| Chr16 | 35515881 | A | G | 169.44 | NON_SYNONYMOUS_CODING | Glyma.16G193000 |
| Chr16 | 33784733 | G | C | 30.24 | NON_SYNONYMOUS_CODING | Glyma.16G176600 |
| Chr16 | 34567166 | G | C | 50.87 | NON_SYNONYMOUS_CODING | Glyma.16G183600 |
| Chr16 | 32159304 | G | A | 237.44 | NON_SYNONYMOUS_CODING | Glyma.16G162700 |
| Chr16 | 32158849 | T | C | 201.87 | NON_SYNONYMOUS_CODING | Glyma.16G162700 |
| Chr16 | 137147 | G | A | 95.87 | NON_SYNONYMOUS_CODING | Glyma.16G002500 |
| Chr16 | 34659466 | G | T | 63.9 | SPLICE_SITE_ACCEPTOR | Glyma.16G184600 |
| Chr16 | 25583886 | A | G | 141.87 | NON_SYNONYMOUS_CODING | Glyma.16G115200 |
| Chr16 | 33162326 | G | A | 78.21 | NON_SYNONYMOUS_CODING | Glyma.16G171200 |
| Chr16 | 10017218 | C | T | 611.19 | NON_SYNONYMOUS_CODING | Glyma.16G085300 |
| Chr16 | 35515880 | C | G | 169.44 | NON_SYNONYMOUS_CODING | Glyma.16G193000 |
| Chr16 | 36528407 | T | A | 354.52 | NON_SYNONYMOUS_CODING | Glyma.16G204300 |
| Chr16 | 31640936 | G | A | 219.86 | NON_SYNONYMOUS_CODING | Glyma.16G156200 |
| Chr16 | 34567057 | G | T | 117.54 | NON_SYNONYMOUS_CODING | Glyma.16G183600 |
| Chr16 | 33627020 | T | A | 34.21 | NON_SYNONYMOUS_CODING | Glyma.16G175100 |
| Chr16 | 32159401 | C | G | 436.28 | NON_SYNONYMOUS_CODING | Glyma.16G162700 |
| Chr16 | 33626972 | G | A | 114.21 | NON_SYNONYMOUS_CODING | Glyma.16G175100 |
| Chr16 | 33784652 | G | T | 57.87 | NON_SYNONYMOUS_CODING | Glyma.16G176600 |
| Chr16 | 25583934 | T | C | 68.23 | NON_SYNONYMOUS_CODING | Glyma.16G115200 |
| Chr16 | 32159454 | T | C | 531.28 | NON_SYNONYMOUS_CODING | Glyma.16G162700 |
| Chr16 | 33784719 | G | T | 33.87 | NON_SYNONYMOUS_CODING | Glyma.16G176600 |
| Chr16 | 35416373 | A | T | 747.21 | NON_SYNONYMOUS_CODING | Glyma.16G191800 |
| Chr16 | 33784650 | C | T | 57.87 | NON_SYNONYMOUS_CODING | Glyma.16G176600 |
| Chr16 | 33162396 | G | A | 93.54 | NON_SYNONYMOUS_CODING | Glyma.16G171200 |
| Chr16 | 32159271 | C | G | 396.86 | NON_SYNONYMOUS_CODING | Glyma.16G162700 |
| Chr16 | 34587348 | G | T | 39.86 | NON_SYNONYMOUS_CODING | Glyma.16G184000 |
| Chr16 | 32159260 | C | T | 396.86 | NON_SYNONYMOUS_CODING | Glyma.16G162700 |
| Chr16 | 32159371 | G | A | 173.37 | STOP_GAINED | Glyma.16G162700 |
| Chr16 | 36236274 | C | T | 102.23 | NON_SYNONYMOUS_CODING | Glyma.16G201000 |
| Chr16 | 32158798 | C | T | 199.44 | NON_SYNONYMOUS_CODING | Glyma.16G162700 |
| Chr16 | 4798992 | C | G | 76.44 | NON_SYNONYMOUS_CODING | Glyma.16G050100 |
| Chr16 | 37182816 | C | T | 289.18 | NON_SYNONYMOUS_CODING | Glyma.16G214000 |
| Chr16 | 32159489 | T | G | 270.32 | NON_SYNONYMOUS_CODING | Glyma.16G162700 |
| Chr16 | 35515882 | C | A | 169.44 | NON_SYNONYMOUS_CODING | Glyma.16G193000 |
| Chr16 | 35416382 | C | A | 747.21 | NON_SYNONYMOUS_CODING | Glyma.16G191800 |
| Chr16 | 27708557 | C | G | 69.87 | NON_SYNONYMOUS_CODING | Glyma.16G126000 |
| Chr16 | 33627781 | C | A | 160.86 | NON_SYNONYMOUS_CODING | Glyma.16G175100 |
| Chr17 | 35736757 | T | C | 101.87 | NON_SYNONYMOUS_CODING | Glyma.17G213500 |
| Chr17 | 33105791 | C | T | 307.53 | NON_SYNONYMOUS_CODING | Glyma.17G204400 |
| Chr17 | 11185695 | C | T | 42.23 | NON_SYNONYMOUS_CODING | Glyma.17G138300 |
| Chr17 | 35736465 | A | G | 95.04 | NON_SYNONYMOUS_CODING | Glyma.17G213500 |
| Chr17 | 34779117 | G | T | 552.52 | START_GAINED | Glyma.17G210800 |
| Chr17 | 35737583 | A | C | 144.87 | NON_SYNONYMOUS_CODING | Glyma.17G213500 |
| Chr17 | 8131046 | C | T | 173.21 | SPLICE_SITE_ACCEPTOR | Glyma.17G103600 |
| Chr17 | 16644738 | T | A | 347.52 | NON_SYNONYMOUS_CODING | Glyma.17G172000 |
| Chr17 | 37811127 | C | T | 371.52 | NON_SYNONYMOUS_CODING | Glyma.17G225200 |
| Chr17 | 24921100 | A | T | 175 | NON_SYNONYMOUS_CODING | Glyma.17G188700 |
| Chr17 | 36053926 | C | A | 85.87 | NON_SYNONYMOUS_CODING | Glyma.17G214600 |
| Chr17 | 12843074 | C | T | 576.52 | NON_SYNONYMOUS_CODING | Glyma.17G153300 |
| Chr17 | 10846244 | C | T | 430.81 | NON_SYNONYMOUS_CODING | Glyma.17G134700 |
| Chr17 | 26889910 | C | T | 311.18 | NON_SYNONYMOUS_CODING | Glyma.17G190900 |
| Chr17 | 36053942 | G | A | 85.87 | NON_SYNONYMOUS_CODING | Glyma.17G214600 |
| Chr17 | 38857683 | G | A | 435.52 | NON_SYNONYMOUS_CODING | Glyma.17G233200 |
| Chr17 | 5291233 | A | C | 108.54 | NON_SYNONYMOUS_CODING | Glyma.17G067800 |
| Chr17 | 5290983 | G | C | 171.2 | NON_SYNONYMOUS_CODING | Glyma.17G067800 |
| Chr17 | 18338517 | C | T | 521.52 | SPLICE_SITE_DONOR | Glyma.17G175500 |
| Chr17 | 36053933 | G | A | 85.87 | NON_SYNONYMOUS_CODING | Glyma.17G214600 |
| Chr17 | 40163934 | A | C | 108.23 | NON_SYNONYMOUS_CODING | Glyma.17G246200 |
| Chr17 | 4892484 | A | T | 39.87 | NON_SYNONYMOUS_CODING | Glyma.17G063600 |
| Chr17 | 7939977 | C | T | 117.23 | NON_SYNONYMOUS_CODING | Glyma.17G101000 |
| Chr17 | 8562388 | C | T | 61.45 | NON_SYNONYMOUS_CODING | Glyma.17G109300 |
| Chr17 | 35736292 | T | C | 90.44 | NON_SYNONYMOUS_CODING | Glyma.17G213500 |
| Chr17 | 35736289 | A | G | 90.44 | NON_SYNONYMOUS_CODING | Glyma.17G213500 |
| Chr17 | 32918725 | T | A | 426.52 | NON_SYNONYMOUS_CODING | Glyma.17G204000 |
| Chr17 | 41491152 | C | A | 32.23 | NON_SYNONYMOUS_CODING | Glyma.17G261200 |
| Chr17 | 36424758 | G | T | 45.87 | STOP_GAINED | Glyma.17G216200 |
| Chr17 | 34558791 | A | G | 67.23 | NON_SYNONYMOUS_CODING | Glyma.17G209500 |
| Chr17 | 9855906 | C | T | 541.19 | NON_SYNONYMOUS_CODING | Glyma.17G123700 |
| Chr17 | 5291093 | T | C | 101.73 | NON_SYNONYMOUS_CODING | Glyma.17G067800 |
| Chr17 | 35878849 | C | A | 289.77 | NON_SYNONYMOUS_CODING | Glyma.17G214300 |
| Chr17 | 36053935 | T | A | 85.87 | NON_SYNONYMOUS_CODING | Glyma.17G214600 |
| Chr17 | 38757474 | C | G | 77.04 | NON_SYNONYMOUS_CODING | Glyma.17G232300 |
| Chr17 | 8278582 | G | A | 497.44 | NON_SYNONYMOUS_CODING | Glyma.17G105500 |
| Chr17 | 10478869 | C | T | 478.17 | NON_SYNONYMOUS_CODING | Glyma.17G130900 |
| Chr17 | 27563855 | A | T | 372.52 | NON_SYNONYMOUS_CODING | Glyma.17G192200 |
| Chr17 | 34558954 | T | A | 125.87 | NON_SYNONYMOUS_CODING | Glyma.17G209500 |
| Chr17 | 35737348 | G | A | 34.19 | NON_SYNONYMOUS_CODING | Glyma.17G213500 |
| Chr17 | 10862779 | C | T | 318.52 | NON_SYNONYMOUS_CODING | Glyma.17G135000 |
| Chr17 | 40163564 | A | C | 39.23 | NON_SYNONYMOUS_CODING | Glyma.17G246200 |
| Chr17 | 38503116 | G | A | 147.91 | NON_SYNONYMOUS_CODING | Glyma.17G230000 |
| Chr17 | 36053953 | T | C | 85.87 | NON_SYNONYMOUS_CODING | Glyma.17G214600 |
| Chr17 | 8891594 | C | T | 118.54 | NON_SYNONYMOUS_CODING | Glyma.17G112600 |
| Chr17 | 36053921 | C | T | 85.87 | NON_SYNONYMOUS_CODING | Glyma.17G214600 |
| Chr17 | 4892476 | C | T | 39.87 | NON_SYNONYMOUS_CODING | Glyma.17G063600 |
| Chr17 | 11874925 | G | A | 81.21 | NON_SYNONYMOUS_CODING | Glyma.17G145100 |
| Chr17 | 5290982 | G | C | 171.2 | NON_SYNONYMOUS_CODING | Glyma.17G067800 |
| Chr17 | 15256892 | C | T | 298.53 | NON_SYNONYMOUS_CODING | Glyma.17G166900 |
| Chr17 | 11874943 | A | G | 81.19 | NON_SYNONYMOUS_CODING | Glyma.17G145100 |
| Chr17 | 36698700 | T | C | 57.87 | NON_SYNONYMOUS_CODING | Glyma.17G217600 |
| Chr17 | 7569411 | G | A | 849.19 | NON_SYNONYMOUS_CODING | Glyma.17G096300 |
| Chr17 | 3732339 | C | G | 120.87 | STOP_GAINED | Glyma.17G049000 |
| Chr17 | 35878839 | T | C | 289.77 | NON_SYNONYMOUS_CODING | Glyma.17G214300 |
| Chr18 | 56407994 | A | C | 563.71 | NON_SYNONYMOUS_CODING | Glyma.18G283200 |
| Chr18 | 49531308 | T | A | 237.87 | NON_SYNONYMOUS_CODING | Glyma.18G209900 |
| Chr18 | 12778942 | C | T | 46.37 | NON_SYNONYMOUS_CODING | Glyma.18G110000 |
| Chr18 | 11947622 | A | G | 131.79 | NON_SYNONYMOUS_CODING | Glyma.18G106600 |
| Chr18 | 56047664 | A | G | 54.19 | NON_SYNONYMOUS_CODING | Glyma.18G279500 |
| Chr18 | 1462199 | C | T | 141.21 | NON_SYNONYMOUS_CODING | Glyma.18G019700 |
| Chr18 | 56074537 | A | G | 81.71 | NON_SYNONYMOUS_CODING | Glyma.18G279800 |
| Chr18 | 57116300 | G | A | 411.19 | NON_SYNONYMOUS_CODING | Glyma.18G292900 |
| Chr18 | 56054837 | T | G | 81.87 | NON_SYNONYMOUS_CODING | Glyma.18G279600 |
| Chr18 | 56054934 | T | C | 96.19 | NON_SYNONYMOUS_CODING | Glyma.18G279600 |
| Chr18 | 15347593 | T | C | 145.86 | NON_SYNONYMOUS_CODING | Glyma.18G121800 |
| Chr18 | 56037732 | C | T | 122.71 | START_GAINED | Glyma.18G279400 |
| Chr18 | 51488352 | A | T | 93.87 | NON_SYNONYMOUS_CODING | Glyma.18G226300 |
| Chr18 | 18648144 | T | C | 242.19 | NON_SYNONYMOUS_CODING | Glyma.18G132600 |
| Chr18 | 51487848 | C | A | 186.87 | NON_SYNONYMOUS_CODING | Glyma.18G226300 |
| Chr18 | 896090 | A | C | 63.37 | NON_SYNONYMOUS_CODING | Glyma.18G012600 |
| Chr18 | 47764957 | G | A | 33.87 | NON_SYNONYMOUS_CODING | Glyma.18G199100 |
| Chr18 | 11947646 | G | A | 131.79 | NON_SYNONYMOUS_CODING | Glyma.18G106600 |
| Chr18 | 51488353 | C | T | 93.87 | NON_SYNONYMOUS_CODING | Glyma.18G226300 |
| Chr18 | 56054909 | A | C | 77.19 | NON_SYNONYMOUS_CODING | Glyma.18G279600 |
| Chr18 | 11947605 | A | G | 131.79 | NON_SYNONYMOUS_CODING | Glyma.18G106600 |
| Chr18 | 8261949 | G | C | 30.72 | NON_SYNONYMOUS_CODING | Glyma.18G084200 |
| Chr18 | 52455274 | A | C | 106.44 | NON_SYNONYMOUS_CODING | Glyma.18G236000 |
| Chr18 | 301777 | T | A | 172.72 | STOP_GAINED | Glyma.18G003600 |
| Chr18 | 37137924 | T | G | 262.93 | NON_SYNONYMOUS_CODING | Glyma.18G163200 |
| Chr18 | 41823120 | C | T | 469.2 | NON_SYNONYMOUS_CODING | Glyma.18G175700 |
| Chr18 | 4402722 | A | G | 237.87 | NON_SYNONYMOUS_CODING | Glyma.18G050800 |
| Chr18 | 56042858 | A | G | 75.87 | NON_SYNONYMOUS_CODING | Glyma.18G279500 |
| Chr18 | 57206823 | A | T | 168.23 | NON_SYNONYMOUS_CODING | Glyma.18G294300 |
| Chr18 | 59885 | T | A | 78.23 | NON_SYNONYMOUS_CODING | Glyma.18G000400 |
| Chr18 | 51487854 | C | A | 95.04 | NON_SYNONYMOUS_CODING | Glyma.18G226300 |
| Chr18 | 51607159 | T | C | 66.37 | NON_SYNONYMOUS_CODING | Glyma.18G226800 |
| Chr18 | 51607167 | C | A | 66.37 | NON_SYNONYMOUS_CODING | Glyma.18G226800 |
| Chr18 | 55743977 | G | C | 35.87 | NON_SYNONYMOUS_CODING | Glyma.18G275200 |
| Chr18 | 56074576 | G | A | 88.19 | NON_SYNONYMOUS_CODING | Glyma.18G279800 |
| Chr18 | 56048470 | G | A | 99.23 | NON_SYNONYMOUS_CODING | Glyma.18G279500 |
| Chr18 | 11947665 | A | G | 131.79 | NON_SYNONYMOUS_CODING | Glyma.18G106600 |
| Chr18 | 55497406 | T | C | 72.23 | NON_SYNONYMOUS_CODING | Glyma.18G271900 |
| Chr18 | 56037791 | G | A | 100.87 | NON_SYNONYMOUS_CODING | Glyma.18G279400 |
| Chr18 | 11947626 | A | G | 131.79 | NON_SYNONYMOUS_CODING | Glyma.18G106600 |
| Chr18 | 53483106 | A | G | 75.87 | NON_SYNONYMOUS_CODING | Glyma.18G247600 |
| Chr18 | 12778891 | G | A | 63.23 | NON_SYNONYMOUS_CODING | Glyma.18G110000 |
| Chr18 | 15347833 | G | A | 45.44 | NON_SYNONYMOUS_CODING | Glyma.18G121800 |
| Chr18 | 49531352 | G | A | 237.87 | SPLICE_SITE_DONOR | Glyma.18G209900 |
| Chr18 | 7999212 | T | C | 83.24 | NON_SYNONYMOUS_CODING | Glyma.18G082200 |
| Chr18 | 56037758 | C | T | 99.84 | START_GAINED | Glyma.18G279400 |
| Chr18 | 52455289 | T | A | 106.44 | NON_SYNONYMOUS_CODING | Glyma.18G236000 |
| Chr18 | 3669759 | G | T | 123.2 | NON_SYNONYMOUS_CODING | Glyma.18G043100 |
| Chr18 | 54118210 | A | G | 51.87 | NON_SYNONYMOUS_CODING | Glyma.18G254900 |
| Chr18 | 55744195 | C | T | 33.87 | NON_SYNONYMOUS_CODING | Glyma.18G275200 |
| Chr18 | 47708114 | T | C | 558.28 | NON_SYNONYMOUS_CODING | Glyma.18G198800 |
| Chr18 | 37138217 | C | T | 187.54 | STOP_GAINED | Glyma.18G163200 |
| Chr18 | 4402790 | A | G | 57.23 | NON_SYNONYMOUS_CODING | Glyma.18G050800 |
| Chr18 | 51488310 | C | G | 54.87 | NON_SYNONYMOUS_CODING | Glyma.18G226300 |
| Chr18 | 56054933 | G | T | 96.19 | NON_SYNONYMOUS_CODING | Glyma.18G279600 |
| Chr18 | 14110147 | G | A | 43.87 | NON_SYNONYMOUS_CODING | Glyma.18G115800 |
| Chr18 | 8602981 | A | G | 202.21 | NON_SYNONYMOUS_CODING | Glyma.18G086600 |
| Chr18 | 52455254 | G | C | 106.44 | NON_SYNONYMOUS_CODING | Glyma.18G236000 |
| Chr18 | 56048796 | G | A | 116.84 | NON_SYNONYMOUS_CODING | Glyma.18G279500 |
| Chr19 | 30701098 | C | T | 34.23 | NON_SYNONYMOUS_CODING | Glyma.19G086300 |
| Chr19 | 42086241 | A | G | 112.23 | NON_SYNONYMOUS_CODING | Glyma.19G159900 |
| Chr19 | 30581309 | A | G | 46.87 | NON_SYNONYMOUS_CODING | Glyma.19G085600 |
| Chr19 | 11330847 | G | A | 71.44 | STOP_GAINED | Glyma.19G058800 |
| Chr19 | 45934154 | T | G | 30.87 | NON_SYNONYMOUS_CODING | Glyma.19G202400 |
| Chr19 | 39743307 | G | A | 62.87 | STOP_GAINED | Glyma.19G136200 |
| Chr19 | 40116286 | C | T | 71.54 | NON_SYNONYMOUS_CODING | Glyma.19G139800 |
| Chr19 | 42086473 | C | T | 68.02 | NON_SYNONYMOUS_CODING | Glyma.19G159900 |
| Chr19 | 42992401 | C | T | 435.21 | NON_SYNONYMOUS_CODING | Glyma.19G169200 |
| Chr19 | 40086025 | G | A | 151.23 | NON_SYNONYMOUS_CODING | Glyma.19G139600 |
| Chr19 | 42276028 | C | T | 277.23 | NON_SYNONYMOUS_CODING | Glyma.19G162100 |
| Chr19 | 30701059 | G | A | 45.23 | NON_SYNONYMOUS_CODING | Glyma.19G086300 |
| Chr19 | 42086182 | G | A | 112.23 | NON_SYNONYMOUS_CODING | Glyma.19G159900 |
| Chr19 | 50083743 | G | A | 83.37 | NON_SYNONYMOUS_CODING | Glyma.19G256800 |
| Chr19 | 30701068 | A | G | 45.23 | NON_SYNONYMOUS_CODING | Glyma.19G086300 |
| Chr19 | 33323501 | G | A | 120.87 | STOP_GAINED | Glyma.19G094200 |
| Chr19 | 40146847 | A | C | 66.87 | NON_SYNONYMOUS_CODING | Glyma.19G140000 |
| Chr19 | 93127 | T | A | 102.23 | NON_SYNONYMOUS_CODING | Glyma.19G001200 |
| Chr19 | 42086263 | C | T | 97.23 | NON_SYNONYMOUS_CODING | Glyma.19G159900 |
| Chr19 | 50673449 | C | T | 472.19 | NON_SYNONYMOUS_CODING | Glyma.19G263900 |
| Chr19 | 2231344 | T | A | 111.87 | NON_SYNONYMOUS_CODING | Glyma.19G020600 |
| Chr19 | 42364832 | C | T | 459.21 | NON_SYNONYMOUS_CODING | Glyma.19G162900 |
| Chr19 | 41359983 | C | T | 262.21 | STOP_GAINED | Glyma.19G153200 |
| Chr19 | 1815665 | T | C | 58.44 | NON_SYNONYMOUS_CODING | Glyma.19G017300 |
| Chr19 | 48269716 | C | T | 46.73 | NON_SYNONYMOUS_CODING | Glyma.19G232500 |
| Chr19 | 326644 | G | A | 416.19 | NON_SYNONYMOUS_CODING | Glyma.19G004100 |
| Chr19 | 37514420 | C | T | 45.87 | START_GAINED | Glyma.19G118300 |
| Chr19 | 39915840 | T | G | 51.23 | NON_SYNONYMOUS_CODING | Glyma.19G137700 |
| Chr19 | 39915852 | G | T | 51.23 | NON_SYNONYMOUS_CODING | Glyma.19G137700 |
| Chr19 | 40116498 | C | G | 33.02 | NON_SYNONYMOUS_CODING | Glyma.19G139800 |
| Chr20 | 35486314 | A | G | 97.87 | NON_SYNONYMOUS_CODING | Glyma.20G112800 |
| Chr20 | 43182995 | C | T | 663.73 | NON_SYNONYMOUS_CODING | Glyma.20G192700 |
| Chr20 | 38079143 | G | A | 161.5 | NON_SYNONYMOUS_CODING | Glyma.20G142200 |
| Chr20 | 36144284 | C | A | 80.47 | NON_SYNONYMOUS_CODING | Glyma.20G118600 |
| Chr20 | 17723515 | G | A | 141.84 | START_GAINED | Glyma.20G058600 |
| Chr20 | 8930019 | G | C | 451.41 | NON_SYNONYMOUS_CODING | Glyma.20G047500 |
| Chr20 | 8930039 | T | A | 384.37 | NON_SYNONYMOUS_CODING | Glyma.20G047500 |
| Chr20 | 45031746 | A | T | 253.86 | NON_SYNONYMOUS_CODING | Glyma.20G214200 |
| Chr20 | 37847705 | G | A | 137.23 | NON_SYNONYMOUS_CODING | Glyma.20G139200 |
| Chr20 | 44555807 | G | C | 157.44 | START_GAINED | Glyma.20G209200 |
| Chr20 | 8930010 | G | T | 451.41 | NON_SYNONYMOUS_CODING | Glyma.20G047500 |
| Chr20 | 44866259 | G | A | 516.2 | NON_SYNONYMOUS_CODING | Glyma.20G212000 |
| Chr20 | 8930035 | A | G | 384.37 | NON_SYNONYMOUS_CODING | Glyma.20G047500 |
| Chr20 | 432279 | G | A | 116.41 | NON_SYNONYMOUS_CODING | Glyma.20G004300 |
| Chr20 | 28467612 | G | A | 106.37 | NON_SYNONYMOUS_CODING | Glyma.20G077700 |
| scaffold_138 | 36418 | A | C | 62.87 | NON_SYNONYMOUS_CODING | Glyma.U005000 |
| scaffold_138 | 36509 | C | T | 67.89 | NON_SYNONYMOUS_CODING | Glyma.U005000 |
| scaffold_195 | 43141 | A | G | 285.44 | NON_SYNONYMOUS_CODING | Glyma.U008000 |
| scaffold_31 | 305986 | G | A | 596.29 | NON_SYNONYMOUS_CODING | Glyma.U032800 |
| scaffold_614 | 4578 | G | A | 39.23 | NON_SYNONYMOUS_CODING | Glyma.U040600 |
| scaffold_76 | 65593 | C | T | 156.86 | NON_SYNONYMOUS_CODING | Glyma.U043000 |

STable 3. InDel in gene with change

| #CHROM | POS | REF | ALT | QUAL | SNPEFF_EFFECT | GENE |
| --- | --- | --- | --- | --- | --- | --- |
| Chr01 | 27893909 | AT | A | 10849.19 | FRAME_SHIFT | Glyma.01G091900 |
| Chr02 | 5906801 | C | CT | 48.37 | FRAME_SHIFT | Glyma.02G066600 |
| Chr03 | 4794284 | T | TCTGG | 388.73 | FRAME_SHIFT | Glyma.03G038800 |
| Chr03 | 5980293 | A | AGTCATTACCTG | 37.19 | FRAME_SHIFT | Glyma.03G047000 |
| Chr03 | 5980288 | G | GAA | 37.19 | FRAME_SHIFT | Glyma.03G047000 |
| Chr03 | 4960121 | T | TGTTGGAAGCCCAGGTTCCACGTTCCAAATTTTTATTCCCATTAATTTATTTTGGAA | 227.44 | FRAME_SHIFT | Glyma.03G039600 |
| Chr04 | 4632737 | GTA | G | 86.87 | SPLICE_SITE_DONOR | Glyma.04G057200 |
| Chr04 | 52093753 | TGAG | T | 51.87 | CODON_DELETION | Glyma.04G254200 |
| Chr04 | 11893472 | T | TGATGTGAACCTGAGTAGGAGTGGATGATGTGAACC | 99.59 | FRAME_SHIFT | Glyma.04G109800 |
| Chr05 | 28038394 | CCAA | C | 30.24 | CODON_DELETION | Glyma.05G105600 |
| Chr06 | 3536217 | CTTA | C | 39.23 | CODON_CHANGE_PLUS_CODON_DELETION | Glyma.06G046800 |
| Chr06 | 3545246 | GTC | G | 43.23 | SPLICE_SITE_DONOR | Glyma.06G047000 |
| Chr07 | 44348164 | GTC | G | 45.87 | SPLICE_SITE_DONOR | Glyma.07G271000 |
| Chr07 | 4698652 | G | GTCC | 126.87 | CODON_CHANGE_PLUS_CODON_INSERTION | Glyma.07G053900 |
| Chr07 | 4698651 | T | TTCACCAATCATTCCA | 126.87 | STOP_GAINED;NON_SYNONYMOUS | Glyma.07G053900 |
| Chr07 | 38568733 | C | CCTT | 305.42 | CODON_INSERTION | Glyma.07G213700 |
| Chr07 | 13568292 | CGTT | C | 33.23 | CODON_DELETION | Glyma.07G119000 |
| Chr07 | 4698645 | A | ACCAC | 123.86 | FRAME_SHIFT | Glyma.07G053900 |
| Chr09 | 2263201 | CAG | C | 240.28 | FRAME_SHIFT | Glyma.09G027700 |
| Chr09 | 45352110 | CTA | C | 55.86 | SPLICE_SITE_ACCEPTOR | Glyma.09G229600 |
| Chr10 | 1627963 | GTCA | G | 30.24 | SPLICE_SITE_DONOR | Glyma.10G018800 |
| Chr10 | 3342067 | CATT | C | 202.28 | CODON_DELETION | Glyma.10G037700 |
| Chr13 | 29862041 | TC | T | 326.85 | FRAME_SHIFT | Glyma.13G184800 |
| Chr13 | 28611944 | A | ATGAGGCACGAGAAGGTAATGCCAACCCAGG | 408.53 | CODON_INSERTION | Glyma.13G172100 |
| Chr13 | 29862036 | A | AT | 331.47 | FRAME_SHIFT | Glyma.13G184800 |
| Chr14 | 45792717 | T | TGAA | 49.37 | CODON_INSERTION | Glyma.14G193200 |
| Chr14 | 21935380 | ATAC | A | 33.23 | CODON_CHANGE_PLUS_CODON_DELETION | Glyma.14G129700 |
| Chr16 | 6645783 | GGAA | G | 40.44 | CODON_CHANGE_PLUS_CODON_DELETION | Glyma.16G067000 |
| Chr16 | 37690497 | TAATCTGGTCGTCGATGAACTTTTTTAATTCCACAGAC | T | 111.33 | FRAME_SHIFT | Glyma.16G219700 |
| Chr17 | 35736147 | A | AC,ACC | 475.59 | SPLICE_SITE_DONOR | Glyma.17G213500 |
| Chr17 | 14502131 | GAAC | G | 37.87 | CODON_DELETION | Glyma.17G162800 |
| Chr17 | 33488360 | CTGTTGTGGTTGCTGCTGCTGCTGCTGT | C | 186.51 | CODON_CHANGE_PLUS_CODON_DELETION | Glyma.17G205700 |
| Chr18 | 39984940 | GTA | G | 122.44 | SPLICE_SITE_DONOR | Glyma.18G170300 |
| Chr18 | 11947648 | AG | A | 131.79 | FRAME_SHIFT | Glyma.18G106600 |
| Chr18 | 41018483 | C | CA | 67.86 | FRAME_SHIFT | Glyma.18G173100 |
| Chr18 | 41018484 | C | CCT | 70.87 | FRAME_SHIFT | Glyma.18G173100 |
| Chr18 | 11947653 | G | GTAGGACCCACA | 131.79 | FRAME_SHIFT | Glyma.18G106600 |
| Chr19 | 45227864 | AAAG | A | 39.23 | CODON_DELETION | Glyma.19G194900 |
| Chr20 | 44073752 | A | AGGTTGATATTTTGGTGGTGATTTTTCATGTGGTGGATTCTCATGAGGTGGTTTGTACTCTGGTGGTGGCTTCTCATGAGGT | 385.61 | CODON_CHANGE_PLUS_CODON_INSERTION | Glyma.20G203800 |

Supplemental Table 4. DEGs between dwarf mutant and control

| Gene_id | readcount_Mutant | readcount_Control | Fold_change(log2) | *P* value | Corrected *P* value | Functional description |
| --- | --- | --- | --- | --- | --- | --- |
| Glyma.01G002600 | 7.83 | 31.94 | -2.03 | 5.26E-05 | 2.41E-03 | Alkaline-phosphatase-like family protein |
| Glyma.01G019800 | 428.35 | 186.66 | 1.20 | 2.11E-21 | 6.49E-19 | Protein of unknown function (duplicated DUF1399) |
| Glyma.01G034000 | 18.25 | 81.34 | -2.16 | 2.08E-11 | 2.93E-09 | MLP-like protein 423 |
| Glyma.01G068000 | 91.48 | 185.18 | -1.02 | 2.73E-09 | 2.98E-07 | Chaperone protein htpG family protein |
| Glyma.01G081300 | 21.70 | 142.98 | -2.72 | 4.52E-24 | 1.72E-21 | alpha-xylosidase 1 |
| Glyma.01G081900 | 2.22 | 25.99 | -3.55 | 1.19E-06 | 8.13E-05 | 2-oxoacid dehydrogenases acyltransferase family protein |
| Glyma.01G086700 | 32.77 | 7.43 | 2.14 | 4.97E-05 | 2.30E-03 | myb-like transcription factor family protein |
| Glyma.01G092300 | 60.66 | 23.70 | 1.36 | 7.70E-05 | 3.36E-03 | -//- |
| Glyma.01G102600 | 166.62 | 74.20 | 1.17 | 6.84E-09 | 7.01E-07 | NAD(P)-linked oxidoreductase superfamily protein |
| Glyma.01G103500 | 21.46 | 58.07 | -1.44 | 1.58E-05 | 8.54E-04 | auxin response factor 9 |
| Glyma.01G106100 | 2.36 | 22.85 | -3.28 | 1.02E-05 | 5.79E-04 | glutathione S-transferase TAU 8 |
| Glyma.01G112600 | 88.40 | 29.04 | 1.61 | 5.22E-08 | 4.64E-06 | Laccase/Diphenol oxidase family protein |
| Glyma.01G113000 | 725.92 | 315.99 | 1.20 | 3.33E-35 | 2.28E-32 | sulfoquinovosyldiacylglycerol 2 |
| Glyma.01G127200 | 62.88 | 19.95 | 1.66 | 2.63E-06 | 1.70E-04 | Disease resistance-responsive (dirigent-like protein) family protein |
| Glyma.01G135500 | 56.69 | 17.65 | 1.68 | 6.35E-06 | 3.77E-04 | SPX domain gene 3 |
| Glyma.01G161500 | 300.24 | 148.22 | 1.02 | 4.42E-12 | 6.66E-10 | Rhodanese/Cell cycle control phosphatase superfamily protein |
| Glyma.01G167700 | 96.91 | 35.93 | 1.43 | 1.82E-07 | 1.44E-05 | -//- |
| Glyma.01G175200 | 5.57 | 27.30 | -2.29 | 5.66E-05 | 2.57E-03 | Sulfite exporter TauE/SafE family protein |
| Glyma.01G186800 | 3.35 | 26.81 | -3.00 | 4.28E-06 | 2.62E-04 | ralf-like 34 |
| Glyma.01G195000 | 1.06 | 18.25 | -4.11 | 1.97E-05 | 1.04E-03 | Galactosyl transferase GMA12/MNN10 family protein |
| Glyma.01G209500 | 0.00 | 21.04 | -6.27 | 1.65E-06 | 1.10E-04 | Pectin lyase-like superfamily protein |
| Glyma.01G214800 | 90.80 | 40.21 | 1.18 | 1.69E-05 | 9.01E-04 | KNOTTED-like homeobox of Arabidopsis thaliana 7 |
| Glyma.01G215500 | 22.45 | 84.38 | -1.91 | 2.51E-10 | 3.08E-08 | hydroxymethylglutaryl-CoA synthase / HMG-CoA synthase / 3-hydroxy-3-methylglutaryl coenzyme A synthase |
| Glyma.01G220600 | 11.69 | 52.91 | -2.18 | 5.40E-08 | 4.78E-06 | plasma membrane intrinsic protein 1;4 |
| Glyma.01G221500 | 103.03 | 36.92 | 1.48 | 3.35E-08 | 3.10E-06 | POX (plant homeobox) family protein |
| Glyma.01G226000 | 33.56 | 82.79 | -1.30 | 1.58E-06 | 1.05E-04 | alpha/beta-Hydrolases superfamily protein |
| Glyma.02G000700 | 5.09 | 25.99 | -2.35 | 6.70E-05 | 2.99E-03 | -//- |
| Glyma.02G001500 | 70.63 | 169.79 | -1.27 | 1.73E-11 | 2.48E-09 | 3-ketoacyl-CoA synthase 11 |
| Glyma.02G008000 | 88.61 | 32.22 | 1.46 | 4.09E-07 | 3.04E-05 | ATP binding cassette subfamily B4 |
| Glyma.02G010600 | 0.00 | 57.36 | -7.39 | 1.71E-14 | 3.16E-12 | glycerol-3-phosphate acyltransferase 3 |
| Glyma.02G015000 | 97.66 | 43.43 | 1.17 | 8.88E-06 | 5.08E-04 | -//- |
| Glyma.02G015500 | 7.89 | 78.41 | -3.31 | 2.21E-16 | 4.53E-14 | P-loop nucleoside triphosphate hydrolases superfamily protein with CH (Calponin Homology) domain |
| Glyma.02G020000 | 9.57 | 44.53 | -2.22 | 4.59E-07 | 3.38E-05 | ubiquitin carrier protein 1/ubiquiting-conjugating enzyme 2 |
| Glyma.02G028200 | 17.46 | 60.58 | -1.79 | 2.67E-07 | 2.04E-05 | Matrixin family protein |
| Glyma.02G046800 | 26.59 | 72.36 | -1.44 | 1.31E-06 | 8.85E-05 | Glycosyl hydrolase family 38 protein |
| Glyma.02G048400 | 75.32 | 291.52 | -1.95 | 8.68E-33 | 5.10E-30 | flavanone 3-hydroxylase |
| Glyma.02G083500 | 26.18 | 114.30 | -2.13 | 3.38E-15 | 6.43E-13 | -//- |
| Glyma.02G104300 | 116.05 | 38.44 | 1.59 | 5.61E-10 | 6.63E-08 | UDP-glycosyltransferase 73B4 |
| Glyma.02G104600 | 17.70 | 61.01 | -1.79 | 2.67E-07 | 2.04E-05 | UDP-glucosyl transferase 73B3 |
| Glyma.02G124500 | 54.57 | 138.28 | -1.34 | 2.32E-10 | 2.86E-08 | Chaperone protein htpG family protein |
| Glyma.02G129900 | 53.89 | 14.54 | 1.89 | 1.73E-06 | 1.14E-04 | -//- |
| Glyma.02G154500 | 63.90 | 24.69 | 1.37 | 4.19E-05 | 1.99E-03 | -//- |
| Glyma.02G156800 | 76.24 | 396.52 | -2.38 | 2.29E-55 | 3.18E-52 | Concanavalin A-like lectin protein kinase family protein |
| Glyma.02G188300 | 79.83 | 30.49 | 1.39 | 3.79E-06 | 2.34E-04 | ABC-2 type transporter family protein |
| Glyma.02G205800 | 52.35 | 11.71 | 2.16 | 2.50E-07 | 1.93E-05 | Cellulose synthase family protein |
| Glyma.02G215700 | 101.63 | 8.84 | 3.52 | 1.05E-20 | 3.07E-18 | matrix metalloproteinase |
| Glyma.02G234200 | 163.65 | 68.54 | 1.26 | 1.05E-09 | 1.19E-07 | Peroxidase superfamily protein |
| Glyma.02G235100 | 129.51 | 60.12 | 1.11 | 1.03E-06 | 7.09E-05 | SOS3-interacting protein 4 |
| Glyma.02G262100 | 70.09 | 19.56 | 1.84 | 8.56E-08 | 7.22E-06 | TRICHOME BIREFRINGENCE-LIKE 33 |
| Glyma.02G264900 | 1.06 | 26.21 | -4.63 | 1.44E-07 | 1.15E-05 | myb domain protein 73 |
| Glyma.02G266600 | 3.21 | 33.31 | -3.37 | 6.94E-08 | 5.97E-06 | GDSL-like Lipase/Acylhydrolase superfamily protein |
| Glyma.02G269400 | 1.13 | 16.73 | -3.89 | 5.78E-05 | 2.61E-03 | glycosyl hydrolase 9B8 |
| Glyma.02G270200 | 47.53 | 161.16 | -1.76 | 1.14E-16 | 2.39E-14 | MD-2-related lipid recognition domain-containing protein / ML domain-containing protein |
| Glyma.02G273300 | 17.70 | 166.40 | -3.23 | 2.43E-32 | 1.39E-29 | 3-oxo-5-alpha-steroid 4-dehydrogenase family protein |
| Glyma.02G282300 | 8.85 | 107.44 | -3.60 | 3.12E-23 | 1.10E-20 | -//- |
| Glyma.02G287400 | 0.00 | 20.37 | -6.90 | 3.34E-06 | 2.10E-04 | protodermal factor 1 |
| Glyma.02G301000 | 3.38 | 23.06 | -2.77 | 4.12E-05 | 1.96E-03 | NOL1/NOP2/sun family protein / antitermination NusB domain-containing protein |
| Glyma.03G008100 | 0.00 | 15.03 | -5.46 | 4.86E-05 | 2.26E-03 | cytochrome P450, family 86, subfamily A, polypeptide 8 |
| Glyma.03G014300 | 21.43 | 88.45 | -2.05 | 1.34E-11 | 1.94E-09 | NAD(P)-binding Rossmann-fold superfamily protein |
| Glyma.03G028000 | 10.01 | 61.04 | -2.61 | 9.91E-11 | 1.30E-08 | Arginase/deacetylase superfamily protein |
| Glyma.03G040100 | 1.61 | 827.00 | -9.01 | 3.94E-158 | 2.05E-154 | lipid transfer protein 1 |
| Glyma.03G066800 | 86.63 | 31.37 | 1.47 | 5.08E-07 | 3.70E-05 | NAD(P)-linked oxidoreductase superfamily protein |
| Glyma.03G077900 | 72.41 | 29.78 | 1.28 | 3.66E-05 | 1.77E-03 | Laccase/Diphenol oxidase family protein |
| Glyma.03G078700 | 40.19 | 94.46 | -1.23 | 8.60E-07 | 6.03E-05 | plasma membrane intrinsic protein 1;4 |
| Glyma.03G086200 | 39.61 | 82.30 | -1.06 | 4.54E-05 | 2.12E-03 | rotamase FKBP 1 |
| Glyma.03G098600 | 155.11 | 51.92 | 1.58 | 1.09E-12 | 1.77E-10 | alkenal reductase |
| Glyma.03G101000 | 74.39 | 32.08 | 1.21 | 6.33E-05 | 2.84E-03 | multidrug resistance-associated protein 9 |
| Glyma.03G101800 | 0.00 | 204.13 | -9.74 | 8.02E-37 | 5.58E-34 | Fatty acid hydroxylase superfamily |
| Glyma.03G103600 | 32.29 | 72.36 | -1.16 | 3.73E-05 | 1.80E-03 | GroES-like family protein |
| Glyma.03G122500 | 49.69 | 17.75 | 1.48 | 1.21E-04 | 5.00E-03 | Major facilitator superfamily protein |
| Glyma.03G129300 | 142.02 | 55.35 | 1.36 | 1.34E-09 | 1.51E-07 | S-adenosyl-L-methionine-dependent methyltransferases superfamily protein |
| Glyma.03G129600 | 3.28 | 32.89 | -3.33 | 1.00E-07 | 8.34E-06 | Glutaredoxin family protein |
| Glyma.03G131700 | 20.85 | 138.00 | -2.73 | 2.42E-23 | 8.78E-21 | Gibberellin-regulated family protein |
| Glyma.03G139900 | 64.07 | 5.13 | 3.64 | 6.05E-14 | 1.07E-11 | -//- |
| Glyma.03G145600 | 20.95 | 1.41 | 3.89 | 1.11E-05 | 6.25E-04 | peroxidase 2 |
| Glyma.03G148100 | 1.06 | 16.98 | -4.00 | 4.38E-05 | 2.06E-03 | Histone superfamily protein |
| Glyma.03G148300 | 40.02 | 11.78 | 1.76 | 8.68E-05 | 3.73E-03 | alpha/beta-Hydrolases superfamily protein |
| Glyma.03G160200 | 41.76 | 11.49 | 1.86 | 3.12E-05 | 1.54E-03 | cytochrome P450, family 94, subfamily B, polypeptide 1 |
| Glyma.03G161300 | 67.05 | 21.82 | 1.62 | 1.84E-06 | 1.21E-04 | poly(ADP-ribose) polymerase 2 |
| Glyma.03G170900 | 0.00 | 96.83 | -9.15 | 1.01E-19 | 2.80E-17 | -//- |
| Glyma.03G171900 | 0.00 | 102.35 | -7.64 | 3.63E-24 | 1.39E-21 | NAD(P)-binding Rossmann-fold superfamily protein |
| Glyma.03G179200 | 41.18 | 6.19 | 2.73 | 1.41E-07 | 1.13E-05 | Seven transmembrane MLO family protein |
| Glyma.03G179900 | 86.56 | 33.85 | 1.35 | 2.37E-06 | 1.53E-04 | FASCICLIN-like arabinogalactan-protein 11 |
| Glyma.03G180000 | 36.94 | 8.77 | 2.07 | 2.46E-05 | 1.26E-03 | FASCICLIN-like arabinogalactan-protein 11 |
| Glyma.03G180300 | 71.18 | 294.24 | -2.05 | 5.15E-35 | 3.47E-32 | -//- |
| Glyma.03G187000 | 38.92 | 9.27 | 2.07 | 1.53E-05 | 8.28E-04 | UDP-glucosyl transferase 73C1 |
| Glyma.03G189800 | 5.67 | 30.91 | -2.45 | 8.79E-06 | 5.04E-04 | Leucine-rich repeat protein kinase family protein |
| Glyma.03G205900 | 58.06 | 18.35 | 1.66 | 6.07E-06 | 3.62E-04 | Peptidase M28 family protein |
| Glyma.03G221300 | 0.00 | 29.28 | -8.74 | 2.99E-07 | 2.27E-05 | glycerol-3-phosphate acyltransferase 2 |
| Glyma.03G221400 | 0.00 | 558.43 | -9.75 | 1.91E-97 | 5.69E-94 | AMP-dependent synthetase and ligase family protein |
| Glyma.03G222000 | 62.02 | 13.16 | 2.24 | 9.47E-09 | 9.50E-07 | galactinol synthase 1 |
| Glyma.03G223600 | 51.84 | 17.54 | 1.56 | 4.43E-05 | 2.07E-03 | RAD-like 6 |
| Glyma.03G230600 | 245.73 | 549.52 | -1.16 | 7.92E-30 | 4.13E-27 | Protein of unknown function, DUF538 |
| Glyma.03G246600 | 8.61 | 32.36 | -1.91 | 8.96E-05 | 3.84E-03 | HXXXD-type acyl-transferase family protein |
| Glyma.03G248400 | 102.28 | 46.65 | 1.13 | 9.58E-06 | 5.43E-04 | NAD(P)-linked oxidoreductase superfamily protein |
| Glyma.03G252200 | 0.00 | 14.64 | -4.84 | 7.47E-05 | 3.28E-03 | Protein of Unknown Function (DUF239) |
| Glyma.03G252700 | 15.69 | 141.11 | -3.17 | 3.07E-27 | 1.38E-24 | GDSL-like Lipase/Acylhydrolase superfamily protein |
| Glyma.04G003200 | 2.97 | 61.25 | -4.36 | 1.99E-15 | 3.82E-13 | plasma membrane intrinsic protein 3 |
| Glyma.04G005100 | 7.24 | 39.11 | -2.43 | 6.20E-07 | 4.45E-05 | Pollen Ole e 1 allergen and extensin family protein |
| Glyma.04G022900 | 67.87 | 263.23 | -1.96 | 8.01E-30 | 4.13E-27 | GDSL-like Lipase/Acylhydrolase superfamily protein |
| Glyma.04G026400 | 346.98 | 64.68 | 2.42 | 3.67E-46 | 3.83E-43 | glyoxal oxidase-related protein |
| Glyma.04G041400 | 0.00 | 18.92 | -4.26 | 1.15E-05 | 6.42E-04 | Leucine-rich repeat protein kinase family protein |
| Glyma.04G063800 | 104.81 | 35.47 | 1.56 | 6.39E-09 | 6.59E-07 | cellulose synthase family protein |
| Glyma.04G091800 | 1.54 | 72.78 | -5.56 | 3.71E-19 | 9.56E-17 | Eukaryotic aspartyl protease family protein |
| Glyma.04G102400 | 1.23 | 37.24 | -4.92 | 2.41E-10 | 2.96E-08 | -//- |
| Glyma.04G103400 | 46.27 | 5.27 | 3.13 | 2.21E-09 | 2.43E-07 | tetratricopeptide repeat (TPR)-containing protein |
| Glyma.04G104600 | 73.88 | 167.88 | -1.18 | 1.97E-10 | 2.47E-08 | Pollen Ole e 1 allergen and extensin family protein |
| Glyma.04G104900 | 98.28 | 335.80 | -1.77 | 2.92E-33 | 1.74E-30 | acetyl-CoA carboxylase 1 |
| Glyma.04G107600 | 1.37 | 17.22 | -3.66 | 6.43E-05 | 2.88E-03 | glutathione S-transferase TAU 8 |
| Glyma.04G122900 | 57.55 | 115.51 | -1.01 | 3.28E-06 | 2.07E-04 | acyl-CoA-binding protein 6 |
| Glyma.04G123700 | 41.31 | 8.95 | 2.21 | 3.41E-06 | 2.14E-04 | solute:sodium symporters;urea transmembrane transporters |
| Glyma.04G123800 | 66.36 | 17.65 | 1.91 | 8.92E-08 | 7.51E-06 | alternative oxidase 1A |
| Glyma.04G136000 | 4.07 | 72.85 | -4.16 | 1.13E-17 | 2.59E-15 | Eukaryotic aspartyl protease family protein |
| Glyma.04G151600 | 36.87 | 98.67 | -1.42 | 2.39E-08 | 2.27E-06 | fatty acyl-ACP thioesterases B |
| Glyma.04G196400 | 46.13 | 13.79 | 1.74 | 2.98E-05 | 1.48E-03 | Major facilitator superfamily protein |
| Glyma.04G227700 | 202.30 | 89.97 | 1.17 | 1.62E-10 | 2.08E-08 | O-methyltransferase 1 |
| Glyma.04G235400 | 15.82 | 50.15 | -1.66 | 8.61E-06 | 4.96E-04 | IQ-domain 21 |
| Glyma.04G255400 | 47.43 | 137.15 | -1.53 | 3.75E-12 | 5.76E-10 | cellulose synthase like G2 |
| Glyma.05G011200 | 23.82 | 167.32 | -2.81 | 8.70E-29 | 4.22E-26 | -//- |
| Glyma.05G027000 | 0.00 | 25.22 | -6.72 | 2.02E-07 | 1.59E-05 | myb domain protein 94 |
| Glyma.05G044600 | 7.59 | 33.77 | -2.15 | 1.59E-05 | 8.55E-04 | GDSL-like Lipase/Acylhydrolase superfamily protein |
| Glyma.05G045200 | 27.20 | 65.96 | -1.28 | 2.41E-05 | 1.23E-03 | nucleoside diphosphate kinase 2 |
| Glyma.05G055200 | 0.00 | 44.95 | -8.78 | 2.36E-10 | 2.91E-08 | Plant invertase/pectin methylesterase inhibitor superfamily protein |
| Glyma.05G056100 | 16.27 | 49.55 | -1.61 | 1.58E-05 | 8.54E-04 | Cupredoxin superfamily protein |
| Glyma.05G068000 | 102.82 | 44.10 | 1.22 | 2.27E-06 | 1.47E-04 | chloroplast beta-amylase |
| Glyma.05G075100 | 0.00 | 15.00 | -7.19 | 7.79E-05 | 3.40E-03 | AMP-dependent synthetase and ligase family protein |
| Glyma.05G085700 | 34.34 | 7.29 | 2.24 | 1.95E-05 | 1.02E-03 | Protein of unknown function (DUF579) |
| Glyma.05G102100 | 48.32 | 114.41 | -1.24 | 5.02E-08 | 4.48E-06 | arabinogalactan protein 18 |
| Glyma.05G110200 | 202.61 | 446.74 | -1.14 | 6.02E-24 | 2.26E-21 | Tubulin/FtsZ family protein/tubulin alpha-4 chain |
| Glyma.05G116300 | 3.11 | 33.14 | -3.41 | 6.55E-08 | 5.68E-06 | GDSL-like Lipase/Acylhydrolase superfamily protein |
| Glyma.05G125800 | 49.72 | 122.19 | -1.30 | 6.14E-09 | 6.34E-07 | FAD-binding Berberine family protein |
| Glyma.05G127700 | 88.64 | 41.52 | 1.09 | 6.29E-05 | 2.83E-03 | O-acetyltransferase family protein |
| Glyma.05G149900 | 23.31 | 67.80 | -1.54 | 9.43E-07 | 6.55E-05 | FKBP-type peptidyl-prolyl cis-trans isomerase family protein |
| Glyma.05G163400 | 17.60 | 50.43 | -1.52 | 2.83E-05 | 1.41E-03 | GDSL-like Lipase/Acylhydrolase superfamily protein |
| Glyma.05G169900 | 10.35 | 90.36 | -3.13 | 8.13E-18 | 1.90E-15 | plantacyanin |
| Glyma.05G196100 | 18.11 | 51.99 | -1.52 | 2.08E-05 | 1.08E-03 | diacylglycerol kinase 2 |
| Glyma.05G203000 | 65.17 | 27.23 | 1.26 | 1.14E-04 | 4.77E-03 | HXXXD-type acyl-transferase family protein |
| Glyma.05G208700 | 47.36 | 176.19 | -1.90 | 9.42E-20 | 2.62E-17 | plasma membrane intrinsic protein 1;4 |
| Glyma.05G210300 | 56.25 | 21.71 | 1.37 | 1.20E-04 | 4.98E-03 | POX (plant homeobox) family protein |
| Glyma.05G216400 | 199.74 | 420.68 | -1.07 | 8.48E-21 | 2.49E-18 | glycosyl hydrolase 9C2 |
| Glyma.05G223400 | 0.00 | 22.99 | -8.39 | 3.63E-06 | 2.26E-04 | S-adenosyl-L-methionine-dependent methyltransferases superfamily protein |
| Glyma.05G233700 | 94.25 | 46.15 | 1.03 | 8.96E-05 | 3.84E-03 | B-box type zinc finger protein with CCT domain |
| Glyma.05G247900 | 128.42 | 59.91 | 1.10 | 1.31E-06 | 8.85E-05 | purple acid phosphatase 17 |
| Glyma.06G017700 | 27.17 | 124.59 | -2.20 | 4.97E-17 | 1.08E-14 | Patched family protein |
| Glyma.06G022800 | 16.85 | 150.31 | -3.16 | 8.14E-29 | 4.00E-26 | GDSL-like Lipase/Acylhydrolase superfamily protein |
| Glyma.06G063500 | 81.36 | 34.73 | 1.23 | 2.39E-05 | 1.23E-03 | response regulator 12 |
| Glyma.06G065000 | 134.40 | 40.85 | 1.72 | 1.72E-12 | 2.72E-10 | cellulose synthase family protein |
| Glyma.06G081100 | 415.26 | 189.70 | 1.13 | 5.35E-19 | 1.36E-16 | BURP domain-containing protein |
| Glyma.06G084200 | 86.11 | 370.56 | -2.11 | 4.03E-45 | 4.10E-42 | Phosphorylase superfamily protein |
| Glyma.06G087100 | 43.36 | 111.51 | -1.36 | 8.52E-09 | 8.61E-07 | alpha/beta-Hydrolases superfamily protein |
| Glyma.06G087800 | 18.83 | 85.51 | -2.18 | 4.46E-12 | 6.69E-10 | NADP-malic enzyme 3 |
| Glyma.06G090500 | 252.36 | 79.82 | 1.66 | 4.13E-21 | 1.25E-18 | tubulin alpha-2 chain |
| Glyma.06G097300 | 51.67 | 192.21 | -1.90 | 2.10E-21 | 6.48E-19 | HXXXD-type acyl-transferase family protein |
| Glyma.06G103700 | 1.30 | 26.06 | -4.33 | 2.34E-07 | 1.82E-05 | -//- |
| Glyma.06G104600 | 79.07 | 6.47 | 3.61 | 9.48E-17 | 2.02E-14 | tetratricopeptide repeat (TPR)-containing protein |
| Glyma.06G109200 | 691.41 | 267.93 | 1.37 | 3.41E-41 | 2.90E-38 | nitrate reductase 1 |
| Glyma.06G116200 | 459.44 | 216.12 | 1.09 | 1.21E-19 | 3.30E-17 | DPP6 N-terminal domain-like protein |
| Glyma.06G129400 | 97.29 | 45.37 | 1.10 | 2.52E-05 | 1.28E-03 | Glycogen/starch synthases, ADP-glucose type |
| Glyma.06G136200 | 190.24 | 86.51 | 1.14 | 1.39E-09 | 1.56E-07 | Polyketide cyclase/dehydrase and lipid transport superfamily protein |
| Glyma.06G137100 | 170.21 | 76.21 | 1.16 | 5.76E-09 | 5.98E-07 | O-methyltransferase 1 |
| Glyma.06G142900 | 92.81 | 190.37 | -1.04 | 9.49E-10 | 1.08E-07 | -//- |
| Glyma.06G143000 | 35.23 | 76.89 | -1.13 | 3.41E-05 | 1.67E-03 | Chalcone-flavanone isomerase family protein |
| Glyma.06G157200 | 2.87 | 21.93 | -2.93 | 3.90E-05 | 1.87E-03 | beta-galactosidase 10 |
| Glyma.06G157400 | 87.58 | 39.50 | 1.15 | 3.40E-05 | 1.67E-03 | NAC (No Apical Meristem) domain transcriptional regulator superfamily protein |
| Glyma.06G158800 | 49.38 | 13.83 | 1.84 | 7.27E-06 | 4.27E-04 | Eukaryotic aspartyl protease family protein |
| Glyma.06G166800 | 177.97 | 60.23 | 1.56 | 3.83E-14 | 6.92E-12 | Nodulin MtN3 family protein/homolog of Medicago truncatula MTN3 |
| Glyma.06G170300 | 191.91 | 468.24 | -1.29 | 1.13E-29 | 5.75E-27 | purple acid phosphatase 27 |
| Glyma.06G202200 | 12.81 | 53.76 | -2.07 | 1.09E-07 | 8.99E-06 | heat shock protein 101 |
| Glyma.06G211600 | 7.65 | 32.04 | -2.07 | 4.20E-05 | 1.99E-03 | Leucine-rich repeat protein kinase family protein |
| Glyma.06G225500 | 66.87 | 25.71 | 1.38 | 2.58E-05 | 1.31E-03 | Cellulose synthase family protein |
| Glyma.06G235000 | 11.28 | 167.32 | -3.89 | 4.62E-37 | 3.32E-34 | UDP-Glycosyltransferase superfamily protein |
| Glyma.06G284900 | 4.51 | 28.08 | -2.64 | 1.02E-05 | 5.75E-04 | Basic-leucine zipper (bZIP) transcription factor family protein |
| Glyma.06G308800 | 18.15 | 51.39 | -1.50 | 2.76E-05 | 1.38E-03 | strictosidine synthase-like 3 |
| Glyma.06G323700 | 90.08 | 35.15 | 1.36 | 1.42E-06 | 9.54E-05 | Sucrose-phosphate synthase family protein |
| Glyma.07G001300 | 127.05 | 261.71 | -1.04 | 5.77E-13 | 9.51E-11 | Terpenoid cyclases family protein |
| Glyma.07G011900 | 74.70 | 28.29 | 1.40 | 6.73E-06 | 3.98E-04 | Pyridoxal phosphate phosphatase-related protein |
| Glyma.07G014500 | 3174.22 | 14769.37 | -2.22 | 0.00E+00 | 0.00E+00 | HAD superfamily, subfamily IIIB acid phosphatase |
| Glyma.07G014600 | 13.29 | 231.19 | -4.12 | 3.25E-52 | 4.37E-49 | HAD superfamily, subfamily IIIB acid phosphatase |
| Glyma.07G018000 | 10.01 | 57.29 | -2.52 | 7.88E-10 | 9.14E-08 | delta tonoplast integral protein |
| Glyma.07G034400 | 76.92 | 28.72 | 1.42 | 3.80E-06 | 2.35E-04 | Galactose oxidase/kelch repeat superfamily protein |
| Glyma.07G034900 | 3.21 | 37.95 | -3.56 | 4.18E-09 | 4.43E-07 | lipoxygenase 1 |
| Glyma.07G049100 | 2.32 | 23.02 | -3.31 | 8.71E-06 | 5.00E-04 | basic helix-loop-helix (bHLH) DNA-binding superfamily protein |
| Glyma.07G069500 | 2.15 | 33.60 | -3.96 | 9.91E-09 | 9.90E-07 | cytochrome P450, family 86, subfamily A, polypeptide 8 |
| Glyma.07G070300 | 56.73 | 17.05 | 1.73 | 3.93E-06 | 2.42E-04 | U-box domain-containing protein kinase family protein |
| Glyma.07G073500 | 35.30 | 7.36 | 2.26 | 1.29E-05 | 7.12E-04 | -//- |
| Glyma.07G073700 | 393.73 | 176.55 | 1.16 | 9.19E-19 | 2.28E-16 | K+ efflux antiporter 2 |
| Glyma.07G088000 | 1.85 | 608.44 | -8.37 | 6.99E-126 | 2.65E-122 | -//- |
| Glyma.07G088300 | 32.70 | 81.98 | -1.33 | 1.31E-06 | 8.85E-05 | Leucine-rich repeat (LRR) family protein |
| Glyma.07G089300 | 139.42 | 62.31 | 1.16 | 1.29E-07 | 1.04E-05 | alpha/beta-Hydrolases superfamily protein |
| Glyma.07G108000 | 0.00 | 20.12 | -5.20 | 2.80E-06 | 1.80E-04 | -//- |
| Glyma.07G115400 | 141.99 | 374.88 | -1.40 | 4.66E-27 | 2.02E-24 | Acyl transferase/acyl hydrolase/lysophospholipase superfamily protein |
| Glyma.07G115500 | 73.91 | 338.38 | -2.19 | 2.12E-43 | 2.01E-40 | Acyl transferase/acyl hydrolase/lysophospholipase superfamily protein |
| Glyma.07G120400 | 16.85 | 52.31 | -1.63 | 7.19E-06 | 4.23E-04 | GroES-like family protein |
| Glyma.07G121900 | 105.25 | 48.31 | 1.12 | 8.21E-06 | 4.77E-04 | multidrug resistance-associated protein 9 |
| Glyma.07G126600 | 3.25 | 71.62 | -4.46 | 6.08E-18 | 1.43E-15 | myo-inositol oxygenase 2/myo-inositol oxygenase 4 |
| Glyma.07G134100 | 54.33 | 16.06 | 1.76 | 5.06E-06 | 3.05E-04 | laccase 17 |
| Glyma.07G139700 | 169.02 | 65.14 | 1.38 | 2.47E-11 | 3.44E-09 | glutathione S-transferase TAU 8 |
| Glyma.07G139800 | 71.01 | 26.77 | 1.41 | 1.05E-05 | 5.93E-04 | glutathione S-transferase TAU 8 |
| Glyma.07G140400 | 355.60 | 166.18 | 1.10 | 9.26E-16 | 1.82E-13 | glutathione S-transferase TAU 8 |
| Glyma.07G152000 | 22.83 | 2.19 | 3.38 | 1.38E-05 | 7.59E-04 | Plant protein of unknown function (DUF828) |
| Glyma.07G156100 | 178.04 | 71.97 | 1.31 | 4.87E-11 | 6.60E-09 | MATE efflux family protein |
| Glyma.07G162900 | 2.77 | 23.16 | -3.07 | 1.59E-05 | 8.55E-04 | allene oxide synthase |
| Glyma.07G163300 | 246.83 | 73.03 | 1.76 | 2.54E-22 | 8.33E-20 | -//- |
| Glyma.07G183200 | 11.76 | 59.24 | -2.33 | 2.09E-09 | 2.32E-07 | UDP-glucosyl transferase 78D2 |
| Glyma.07G200200 | 20.67 | 75.90 | -1.88 | 3.03E-09 | 3.27E-07 | HSP20-like chaperones superfamily protein |
| Glyma.07G201100 | 11.14 | 43.08 | -1.95 | 4.61E-06 | 2.80E-04 | -//- |
| Glyma.07G201600 | 8.75 | 47.71 | -2.45 | 3.32E-08 | 3.08E-06 | HAESA-like 1 |
| Glyma.07G205400 | 21.90 | 2.51 | 3.12 | 3.96E-05 | 1.89E-03 | Cysteine proteinases superfamily protein |
| Glyma.07G229000 | 2.80 | 22.07 | -2.98 | 3.24E-05 | 1.60E-03 | expansin A15 |
| Glyma.07G239700 | 5.43 | 35.12 | -2.69 | 6.08E-07 | 4.38E-05 | ATP citrate lyase subunit B 2 |
| Glyma.07G242600 | 0.00 | 35.79 | -8.03 | 3.88E-09 | 4.12E-07 | myb domain protein 111 |
| Glyma.07G253700 | 108.36 | 41.84 | 1.37 | 9.44E-08 | 7.91E-06 | pyruvate orthophosphate dikinase |
| Glyma.07G254600 | 10.87 | 49.58 | -2.19 | 1.29E-07 | 1.04E-05 | UDP-Glycosyltransferase superfamily protein |
| Glyma.08G004100 | 69.78 | 25.78 | 1.44 | 9.05E-06 | 5.16E-04 | SAUR-like auxin-responsive protein family |
| Glyma.08G009900 | 45.28 | 1.77 | 4.68 | 1.11E-11 | 1.61E-09 | homolog of Medicago truncatula MTN3 |
| Glyma.08G015600 | 64.14 | 146.03 | -1.19 | 2.76E-09 | 2.99E-07 | cytochrome P450, family 86, subfamily A, polypeptide 7 |
| Glyma.08G021700 | 0.00 | 19.03 | -4.87 | 6.17E-06 | 3.67E-04 | HMG (high mobility group) box protein |
| Glyma.08G049200 | 35.91 | 96.83 | -1.43 | 2.71E-08 | 2.54E-06 | -//- |
| Glyma.08G056200 | 122.30 | 53.61 | 1.19 | 4.54E-07 | 3.35E-05 | Protein of unknown function (DUF760) |
| Glyma.08G065200 | 2.19 | 18.96 | -3.12 | 8.41E-05 | 3.64E-03 | -//- |
| Glyma.08G070800 | 340.46 | 128.27 | 1.41 | 4.75E-22 | 1.54E-19 | white-brown complex homolog protein 11 |
| Glyma.08G070900 | 0.00 | 24.01 | -9.46 | 9.28E-06 | 5.27E-04 | white-brown complex homolog protein 11 |
| Glyma.08G083700 | 1.78 | 21.75 | -3.61 | 7.73E-06 | 4.52E-04 | -//- |
| Glyma.08G116900 | 37.15 | 99.06 | -1.42 | 2.43E-08 | 2.30E-06 | Granulin repeat cysteine protease family protein |
| Glyma.08G137000 | 25.12 | 221.78 | -3.14 | 1.52E-41 | 1.32E-38 | Lipase/lipooxygenase, PLAT/LH2 family protein |
| Glyma.08G152800 | 53.72 | 193.63 | -1.85 | 6.29E-21 | 1.86E-18 | Leucine-rich repeat (LRR) family protein |
| Glyma.08G159900 | 1.26 | 32.01 | -4.66 | 5.88E-09 | 6.08E-07 | ATP-citrate lyase A-1 |
| Glyma.08G175200 | 204.38 | 79.50 | 1.36 | 3.23E-13 | 5.51E-11 | glutathione S-transferase TAU 19 |
| Glyma.08G176600 | 1.13 | 33.21 | -4.88 | 2.33E-09 | 2.55E-07 | Thiamine pyrophosphate dependent pyruvate decarboxylase family protein |
| Glyma.08G178200 | 19.14 | 66.13 | -1.79 | 8.09E-08 | 6.85E-06 | UDP-glucosyl transferase 85A2 |
| Glyma.08G179600 | 73.16 | 23.59 | 1.63 | 5.34E-07 | 3.89E-05 | Peroxidase superfamily protein |
| Glyma.08G181000 | 55.94 | 177.25 | -1.66 | 6.07E-17 | 1.30E-14 | UDP-Glycosyltransferase superfamily protein |
| Glyma.08G195000 | 88.34 | 37.98 | 1.22 | 1.24E-05 | 6.86E-04 | Pyridoxal phosphate phosphatase-related protein |
| Glyma.08G195100 | 399.68 | 184.43 | 1.12 | 5.59E-18 | 1.32E-15 | Pyridoxal phosphate phosphatase-related protein |
| Glyma.08G200000 | 0.00 | 13.97 | -6.09 | 9.12E-05 | 3.90E-03 | HAD superfamily, subfamily IIIB acid phosphatase /vegetative storage protein 1 |
| Glyma.08G200100 | 97.46 | 1021.01 | -3.39 | 1.48E-196 | 1.24E-192 | HAD superfamily, subfamily IIIB acid phosphatase |
| Glyma.08G201300 | 65.54 | 24.26 | 1.43 | 1.74E-05 | 9.24E-04 | ABC-2 and Plant PDR ABC-type transporter family protein/pleiotropic drug resistance 7 |
| Glyma.08G203000 | 6.63 | 30.98 | -2.22 | 2.52E-05 | 1.28E-03 | delta tonoplast integral protein |
| Glyma.08G220000 | 87.14 | 32.61 | 1.42 | 9.09E-07 | 6.34E-05 | Chaperone DnaJ-domain superfamily protein |
| Glyma.08G220200 | 215.35 | 96.37 | 1.16 | 5.59E-11 | 7.53E-09 | hydroxycinnamoyl-CoA shikimate/quinate hydroxycinnamoyl transferase |
| Glyma.08G237500 | 11.35 | 37.13 | -1.71 | 9.58E-05 | 4.06E-03 | FTSH protease 12 |
| Glyma.08G249800 | 52.25 | 14.54 | 1.85 | 3.64E-06 | 2.26E-04 | COBRA-like extracellular glycosyl-phosphatidyl inositol-anchored protein family |
| Glyma.08G261100 | 3.96 | 35.51 | -3.16 | 6.03E-08 | 5.27E-06 | 3-ketoacyl-CoA synthase 6 |
| Glyma.08G262800 | 3.45 | 37.84 | -3.45 | 6.60E-09 | 6.78E-07 | alpha-xylosidase 1 |
| Glyma.08G263200 | 28.50 | 5.23 | 2.44 | 3.97E-05 | 1.89E-03 | DNA glycosylase superfamily protein |
| Glyma.08G279700 | 11.58 | 141.96 | -3.62 | 3.02E-30 | 1.63E-27 | Protein-tyrosine phosphatase-like, PTPLA |
| Glyma.08G282800 | 93.08 | 36.85 | 1.34 | 1.28E-06 | 8.74E-05 | Copper amine oxidase family protein |
| Glyma.08G285400 | 14.90 | 57.61 | -1.95 | 1.17E-07 | 9.54E-06 | Pectin lyase-like superfamily protein |
| Glyma.08G285900 | 276.45 | 127.11 | 1.12 | 5.40E-13 | 8.94E-11 | DC1 domain-containing protein |
| Glyma.08G287500 | 4.65 | 45.55 | -3.29 | 4.29E-10 | 5.12E-08 | polygalacturonase 2 |
| Glyma.08G292700 | 32.40 | 74.73 | -1.21 | 1.67E-05 | 8.93E-04 | -//- |
| Glyma.08G293100 | 103.64 | 31.02 | 1.74 | 3.99E-10 | 4.79E-08 | SIN3-like 3 |
| Glyma.08G293800 | 12.37 | 94.36 | -2.93 | 1.46E-17 | 3.30E-15 | -//- |
| Glyma.08G319200 | 0.00 | 15.81 | -4.27 | 5.99E-05 | 2.70E-03 | -//- |
| Glyma.08G321100 | 706.85 | 219.59 | 1.69 | 2.26E-57 | 3.25E-54 | Eukaryotic aspartyl protease family protein |
| Glyma.08G329900 | 23.92 | 75.75 | -1.66 | 4.61E-08 | 4.14E-06 | FASCICLIN-like arabinogalactan 1 |
| Glyma.08G333700 | 65.68 | 24.15 | 1.44 | 1.55E-05 | 8.37E-04 | Tetratricopeptide repeat (TPR)-like superfamily protein |
| Glyma.08G334000 | 221.09 | 93.15 | 1.25 | 1.76E-12 | 2.77E-10 | alpha-glucan phosphorylase 2 |
| Glyma.08G339500 | 175.68 | 77.66 | 1.18 | 2.03E-09 | 2.26E-07 | -//- |
| Glyma.08G341100 | 0.00 | 72.25 | -7.59 | 1.33E-17 | 3.04E-15 | kunitz trypsin inhibitor 1 |
| Glyma.08G341600 | 78.56 | 191.86 | -1.29 | 4.28E-13 | 7.14E-11 | kunitz trypsin inhibitor 1 |
| Glyma.08G341700 | 36.22 | 77.88 | -1.10 | 3.97E-05 | 1.89E-03 | kunitz trypsin inhibitor 1 |
| Glyma.08G344300 | 3.69 | 22.00 | -2.58 | 1.15E-04 | 4.79E-03 | Bifunctional inhibitor/lipid-transfer protein/seed storage 2S albumin superfamily protein |
| Glyma.08G356800 | 1.09 | 17.08 | -3.97 | 4.36E-05 | 2.05E-03 | Pectin lyase-like superfamily protein |
| Glyma.09G013800 | 0.00 | 13.83 | -4.85 | 1.17E-04 | 4.88E-03 | Heavy metal transport/detoxification superfamily protein |
| Glyma.09G023800 | 47.64 | 16.27 | 1.55 | 1.00E-04 | 4.24E-03 | nuclear factor Y, subunit A7 |
| Glyma.09G038500 | 138.70 | 50.22 | 1.47 | 2.05E-10 | 2.55E-08 | chitinase-like protein 2 |
| Glyma.09G038900 | 0.00 | 25.96 | -5.70 | 9.08E-08 | 7.63E-06 | myb domain protein 12 |
| Glyma.09G040800 | 1.71 | 17.90 | -3.39 | 7.53E-05 | 3.30E-03 | MLP-like protein 423 |
| Glyma.09G047200 | 132.52 | 50.96 | 1.38 | 3.18E-09 | 3.41E-07 | plasma-membrane associated cation-binding protein 1 |
| Glyma.09G051100 | 217.13 | 63.23 | 1.78 | 3.59E-20 | 1.02E-17 | cellulose synthase A4 |
| Glyma.09G062600 | 552.80 | 165.02 | 1.74 | 1.94E-47 | 2.08E-44 | xyloglucan endotransglycosylase 6 |
| Glyma.09G069800 | 738.43 | 271.89 | 1.44 | 1.59E-47 | 1.75E-44 | Cysteine proteinases superfamily protein |
| Glyma.09G070500 | 77.06 | 21.93 | 1.81 | 2.80E-08 | 2.62E-06 | strictosidine synthase-like 4 |
| Glyma.09G073000 | 124.56 | 55.74 | 1.16 | 6.20E-07 | 4.45E-05 | -//- |
| Glyma.09G077600 | 61.61 | 124.59 | -1.02 | 1.10E-06 | 7.56E-05 | Ribosomal L18p/L5e family protein |
| Glyma.09G130400 | 94.04 | 43.43 | 1.11 | 2.82E-05 | 1.41E-03 | Heavy metal transport/detoxification superfamily protein |
| Glyma.09G155500 | 12.13 | 101.82 | -3.07 | 1.40E-19 | 3.76E-17 | Kunitz family trypsin and protease inhibitor protein |
| Glyma.09G160000 | 86.46 | 237.20 | -1.46 | 1.24E-18 | 3.05E-16 | white-brown complex homolog protein 11 |
| Glyma.09G164000 | 4.13 | 173.33 | -5.39 | 3.12E-43 | 2.89E-40 | -//- |
| Glyma.09G178100 | 3.76 | 23.13 | -2.62 | 6.54E-05 | 2.93E-03 | B-S glucosidase 44 |
| Glyma.09G184000 | 0.00 | 118.65 | -9.76 | 5.05E-22 | 1.62E-19 | alpha/beta-Hydrolases superfamily protein |
| Glyma.09G188600 | 21.94 | 76.89 | -1.81 | 5.61E-09 | 5.84E-07 | Leucine-rich repeat (LRR) family protein |
| Glyma.09G188800 | 3.59 | 2345.43 | -9.35 | 0.00E+00 | 0.00E+00 | -//- |
| Glyma.09G196000 | 9.77 | 83.11 | -3.09 | 2.38E-16 | 4.86E-14 | hydroxy methylglutaryl CoA reductase 1 |
| Glyma.09G198900 | 79.48 | 36.71 | 1.11 | 1.18E-04 | 4.91E-03 | Disease resistance-responsive (dirigent-like protein) family protein |
| Glyma.09G202600 | 198.40 | 86.26 | 1.20 | 9.20E-11 | 1.22E-08 | Protein of unknown function (duplicated DUF1399) |
| Glyma.09G206300 | 11.86 | 66.06 | -2.48 | 5.93E-11 | 7.96E-09 | P-glycoprotein 2 |
| Glyma.09G208300 | 85.53 | 18.81 | 2.18 | 3.15E-11 | 4.35E-09 | xyloglucan endotransglucosylase/hydrolase 30 |
| Glyma.09G231900 | 721.48 | 326.85 | 1.14 | 2.30E-32 | 1.34E-29 | Plant invertase/pectin methylesterase inhibitor superfamily protein |
| Glyma.09G236200 | 7.04 | 43.57 | -2.63 | 4.04E-08 | 3.68E-06 | expansin A1 |
| Glyma.09G238300 | 27.65 | 149.85 | -2.44 | 1.52E-22 | 5.10E-20 | GAST1 protein homolog 4/Gibberellin-regulated family protein |
| Glyma.09G247700 | 1.20 | 19.27 | -4.01 | 1.32E-05 | 7.26E-04 | -//- |
| Glyma.09G267700 | 17.84 | 53.44 | -1.58 | 9.14E-06 | 5.20E-04 | FASCICLIN-like arabinogalactan-protein 10 |
| Glyma.09G273400 | 3.08 | 29.71 | -3.27 | 4.95E-07 | 3.62E-05 | alpha-galactosidase 1 |
| Glyma.09G278000 | 3.59 | 40.99 | -3.51 | 1.23E-09 | 1.39E-07 | lipid transfer protein 1 |
| Glyma.10G000500 | 11.82 | 46.82 | -1.99 | 1.36E-06 | 9.19E-05 | -//- |
| Glyma.10G001800 | 7.31 | 89.09 | -3.61 | 1.49E-19 | 3.98E-17 | 3-ketoacyl-CoA synthase 11 |
| Glyma.10G010800 | 65.23 | 148.18 | -1.18 | 2.27E-09 | 2.50E-07 | AMP-dependent synthetase and ligase family protein |
| Glyma.10G011000 | 0.00 | 68.15 | -6.79 | 1.49E-17 | 3.33E-15 | glycerol-3-phosphate acyltransferase 3 |
| Glyma.10G013700 | 58.71 | 19.17 | 1.61 | 8.44E-06 | 4.88E-04 | Adenine nucleotide alpha hydrolases-like superfamily protein |
| Glyma.10G016000 | 4.51 | 64.47 | -3.84 | 3.83E-15 | 7.26E-13 | P-loop nucleoside triphosphate hydrolases superfamily protein with CH (Calponin Homology) domain |
| Glyma.10G019000 | 453.26 | 185.60 | 1.29 | 3.39E-25 | 1.40E-22 | multidrug resistance-associated protein 4 |
| Glyma.10G019800 | 99.10 | 42.72 | 1.21 | 3.88E-06 | 2.39E-04 | -//- |
| Glyma.10G042500 | 1.54 | 22.78 | -3.89 | 2.71E-06 | 1.74E-04 | GDSL-like Lipase/Acylhydrolase superfamily protein |
| Glyma.10G043700 | 13.40 | 217.15 | -4.02 | 1.74E-48 | 1.96E-45 | NAD(P)-binding Rossmann-fold superfamily protein |
| Glyma.10G090100 | 12.99 | 41.77 | -1.69 | 4.21E-05 | 1.99E-03 | Protein of unknown function, DUF642 |
| Glyma.10G112000 | 189.18 | 61.18 | 1.63 | 8.55E-16 | 1.68E-13 | copper chaperone |
| Glyma.10G125000 | 11.07 | 65.18 | -2.56 | 3.73E-11 | 5.10E-09 | Pectin lyase-like superfamily protein |
| Glyma.10G132500 | 1.06 | 166.64 | -7.30 | 2.51E-39 | 2.06E-36 | Bifunctional inhibitor/lipid-transfer protein/seed storage 2S albumin superfamily protein |
| Glyma.10G139200 | 48.05 | 97.57 | -1.02 | 1.48E-05 | 8.03E-04 | Pectinacetylesterase family protein |
| Glyma.10G147600 | 115.40 | 246.54 | -1.10 | 3.72E-13 | 6.29E-11 | calreticulin 1b |
| Glyma.10G159700 | 42.61 | 10.82 | 1.98 | 1.15E-05 | 6.42E-04 | cysteine synthase D1 |
| Glyma.10G167800 | 92.03 | 37.81 | 1.28 | 3.19E-06 | 2.02E-04 | ammonium transporter 1;2 |
| Glyma.10G179400 | 20.54 | 196.49 | -3.26 | 4.01E-38 | 3.16E-35 | 3-ketoacyl-CoA synthase 11 |
| Glyma.10G186500 | 107.98 | 41.84 | 1.37 | 1.08E-07 | 8.93E-06 | phosphate transporter 1;7 |
| Glyma.10G192900 | 104.12 | 36.43 | 1.52 | 1.59E-08 | 1.55E-06 | glutathione S-transferase TAU 15 |
| Glyma.10G193500 | 998.20 | 419.79 | 1.25 | 5.84E-51 | 7.38E-48 | Fe superoxide dismutase 2 |
| Glyma.10G194000 | 6.90 | 144.19 | -4.38 | 3.17E-34 | 2.04E-31 | UDP-Glycosyltransferase superfamily protein |
| Glyma.10G200800 | 147.90 | 360.84 | -1.29 | 3.07E-23 | 1.09E-20 | cytochrome P450, family 76, subfamily C, polypeptide 4 |
| Glyma.10G211300 | 64.11 | 21.29 | 1.59 | 4.21E-06 | 2.57E-04 | PATATIN-like protein 6 |
| Glyma.10G212300 | 54.85 | 19.56 | 1.49 | 5.25E-05 | 2.41E-03 | Seven transmembrane MLO family protein |
| Glyma.10G223400 | 80.89 | 166.26 | -1.04 | 1.00E-08 | 1.00E-06 | ribosomal protein 1 |
| Glyma.10G224300 | 161.81 | 75.93 | 1.09 | 6.84E-08 | 5.89E-06 | -//- |
| Glyma.10G241600 | 1.61 | 18.71 | -3.54 | 3.83E-05 | 1.84E-03 | subtilase 1.3 |
| Glyma.10G242600 | 193.24 | 85.62 | 1.17 | 3.54E-10 | 4.27E-08 | ATP sulfurylase 1 |
| Glyma.10G245100 | 50.30 | 4.88 | 3.37 | 1.19E-10 | 1.54E-08 | Leucine-rich repeat (LRR) family protein |
| Glyma.10G245400 | 14.86 | 44.28 | -1.57 | 5.73E-05 | 2.60E-03 | Kinase interacting (KIP1-like) family protein |
| Glyma.10G262700 | 136.89 | 56.30 | 1.28 | 1.38E-08 | 1.35E-06 | UDP-glucosyl transferase 84B1 |
| Glyma.10G274400 | 382.45 | 774.48 | -1.02 | 4.50E-34 | 2.80E-31 | 3-ketoacyl-CoA synthase 6 |
| Glyma.11G000500 | 179.06 | 50.50 | 1.83 | 1.76E-17 | 3.91E-15 | UDP-glucosyl transferase 73B3 |
| Glyma.11G001700 | 0.00 | 17.75 | -4.56 | 1.58E-05 | 8.54E-04 | Radical SAM superfamily protein |
| Glyma.11G020700 | 66.33 | 27.16 | 1.29 | 7.30E-05 | 3.21E-03 | respiratory burst oxidase protein F |
| Glyma.11G022100 | 0.00 | 39.33 | -6.36 | 6.12E-11 | 8.19E-09 | Protein of unknown function (DUF604) |
| Glyma.11G022200 | 102.28 | 36.25 | 1.50 | 2.90E-08 | 2.70E-06 | POX (plant homeobox) family protein |
| Glyma.11G037200 | 0.00 | 26.67 | -6.29 | 6.95E-08 | 5.97E-06 | HXXXD-type acyl-transferase family protein |
| Glyma.11G046800 | 6.19 | 43.61 | -2.82 | 1.28E-08 | 1.26E-06 | Galactosyl transferase GMA12/MNN10 family protein |
| Glyma.11G055200 | 3.25 | 22.53 | -2.79 | 4.66E-05 | 2.17E-03 | ralf-like 34 |
| Glyma.11G061100 | 99.44 | 42.76 | 1.22 | 3.53E-06 | 2.21E-04 | -//- |
| Glyma.11G077400 | 42.51 | 12.02 | 1.82 | 3.49E-05 | 1.71E-03 | Chaperone DnaJ-domain superfamily protein |
| Glyma.11G093900 | 43.23 | 158.51 | -1.87 | 1.08E-17 | 2.48E-15 | ABC-2 type transporter family protein |
| Glyma.11G099000 | 0.00 | 24.97 | -4.87 | 2.25E-07 | 1.76E-05 | nucleolar essential protein-related |
| Glyma.11G101400 | 25.39 | 70.17 | -1.47 | 1.46E-06 | 9.79E-05 | NAD(P)-binding Rossmann-fold superfamily protein |
| Glyma.11G119000 | 29.76 | 0.00 | 8.13 | 1.27E-07 | 1.03E-05 | -//- |
| Glyma.11G123300 | 64.24 | 26.42 | 1.28 | 1.01E-04 | 4.25E-03 | -//- |
| Glyma.11G130900 | 0.00 | 19.84 | -4.72 | 4.37E-06 | 2.67E-04 | Histone superfamily protein |
| Glyma.11G133300 | 0.00 | 122.90 | -7.91 | 4.75E-28 | 2.23E-25 | aldehyde dehydrogenase 3F1 |
| Glyma.11G139100 | 20.61 | 61.68 | -1.58 | 1.90E-06 | 1.24E-04 | expansin A4 |
| Glyma.11G148100 | 34.51 | 9.23 | 1.90 | 1.21E-04 | 4.98E-03 | FASCICLIN-like arabinogalactan-protein 11 |
| Glyma.11G150300 | 9.57 | 38.87 | -2.02 | 8.51E-06 | 4.91E-04 | Disease resistance-responsive (dirigent-like protein) family protein |
| Glyma.11G150400 | 66.19 | 197.20 | -1.57 | 2.01E-17 | 4.44E-15 | Disease resistance-responsive (dirigent-like protein) family protein |
| Glyma.11G160000 | 62.12 | 133.93 | -1.11 | 6.52E-08 | 5.68E-06 | expansin A8 |
| Glyma.11G164000 | 0.00 | 18.89 | -8.11 | 2.03E-05 | 1.06E-03 | laccase 3 |
| Glyma.11G179300 | 45.45 | 14.54 | 1.64 | 7.11E-05 | 3.14E-03 | FAD/NAD(P)-binding oxidoreductase family protein |
| Glyma.11G179600 | 5.19 | 26.42 | -2.35 | 5.97E-05 | 2.69E-03 | NIMA (never in mitosis, gene A)-related 6 |
| Glyma.11G192700 | 70.12 | 188.36 | -1.43 | 1.05E-14 | 1.95E-12 | copper/zinc superoxide dismutase 2 |
| Glyma.11G207000 | 65.58 | 20.97 | 1.64 | 1.82E-06 | 1.20E-04 | cysteine-rich RLK (RECEPTOR-like protein kinase) 2 |
| Glyma.11G208600 | 0.00 | 41.80 | -8.67 | 7.85E-10 | 9.12E-08 | Uncharacterised protein family (UPF0497) |
| Glyma.11G213500 | 35.68 | 6.26 | 2.51 | 3.01E-06 | 1.92E-04 | TPX2 (targeting protein for Xklp2) protein family |
| Glyma.11G219400 | 0.00 | 20.44 | -4.52 | 3.76E-06 | 2.33E-04 | Barwin-like endoglucanases superfamily protein |
| Glyma.11G238400 | 58.57 | 23.09 | 1.34 | 1.16E-04 | 4.83E-03 | slufate transporter 2;1 |
| Glyma.11G244900 | 0.00 | 17.75 | -6.44 | 1.14E-05 | 6.40E-04 | Pectate lyase family protein |
| Glyma.11G245600 | 9.02 | 83.71 | -3.21 | 5.44E-17 | 1.18E-14 | beta-ketoacyl reductase 1 |
| Glyma.11G247600 | 176.64 | 76.53 | 1.21 | 8.54E-10 | 9.82E-08 | glyceraldehyde-3-phosphate dehydrogenase C subunit 1 |
| Glyma.11G251100 | 85.64 | 345.88 | -2.01 | 4.65E-40 | 3.88E-37 | glycosylphosphatidylinositol-anchored lipid protein transfer 1 |
| Glyma.12G014400 | 387.75 | 187.30 | 1.05 | 6.59E-16 | 1.31E-13 | Nucleotide-diphospho-sugar transferases superfamily protein |
| Glyma.12G044700 | 120.35 | 41.24 | 1.55 | 6.90E-10 | 8.05E-08 | polyol/monosaccharide transporter 5 |
| Glyma.12G055200 | 0.00 | 14.96 | -4.69 | 6.81E-05 | 3.03E-03 | Histone superfamily protein |
| Glyma.12G057400 | 57.31 | 124.21 | -1.12 | 1.68E-07 | 1.33E-05 | aldehyde dehydrogenase 3F1 |
| Glyma.12G065400 | 9.98 | 37.24 | -1.90 | 2.81E-05 | 1.41E-03 | Histone superfamily protein |
| Glyma.12G069400 | 21.53 | 1.20 | 4.16 | 5.34E-06 | 3.22E-04 | FASCICLIN-like arabinogalactan-protein 11 |
| Glyma.12G072200 | 9.77 | 38.44 | -1.98 | 1.28E-05 | 7.08E-04 | ROP interactive partner 5 |
| Glyma.12G081300 | 49.28 | 151.40 | -1.62 | 3.27E-14 | 5.96E-12 | copper/zinc superoxide dismutase 2 |
| Glyma.12G096900 | 35.81 | 77.59 | -1.12 | 3.57E-05 | 1.74E-03 | cellulose synthase-like B3 |
| Glyma.12G114900 | 123.70 | 54.53 | 1.18 | 4.54E-07 | 3.35E-05 | O-acyltransferase (WSD1-like) family protein |
| Glyma.12G132800 | 5.16 | 343.37 | -6.06 | 6.84E-84 | 1.78E-80 | fatty alcohol oxidase 3 |
| Glyma.12G173800 | 104.36 | 30.38 | 1.78 | 1.78E-10 | 2.26E-08 | FASCICLIN-like arabinogalactan-protein 11 |
| Glyma.12G178800 | 9.09 | 34.84 | -1.94 | 4.08E-05 | 1.94E-03 | copper/zinc superoxide dismutase 2 |
| Glyma.12G184400 | 34.82 | 8.49 | 2.04 | 5.22E-05 | 2.41E-03 | alpha/beta-Hydrolases superfamily protein |
| Glyma.12G187200 | 0.00 | 18.78 | -7.52 | 1.26E-05 | 6.97E-04 | -//- |
| Glyma.12G194000 | 66.05 | 27.27 | 1.28 | 8.52E-05 | 3.67E-03 | MATE efflux family protein |
| Glyma.12G217400 | 229.60 | 713.08 | -1.63 | 1.02E-61 | 1.70E-58 | BURP domain-containing protein |
| Glyma.12G221000 | 317.15 | 140.61 | 1.17 | 9.65E-16 | 1.88E-13 | Peroxisomal membrane 22 kDa (Mpv17/PMP22) family protein |
| Glyma.12G225500 | 30.28 | 76.25 | -1.33 | 2.81E-06 | 1.80E-04 | early nodulin-like protein 1 |
| Glyma.13G011900 | 2.02 | 179.13 | -6.47 | 4.14E-44 | 4.02E-41 | -//- |
| Glyma.13G012100 | 0.00 | 26.91 | -5.75 | 5.28E-08 | 4.68E-06 | -//- |
| Glyma.13G012200 | 0.00 | 72.64 | -7.18 | 3.36E-18 | 8.12E-16 | -//- |
| Glyma.13G013600 | 0.00 | 89.94 | -7.36 | 6.89E-22 | 2.18E-19 | -//- |
| Glyma.13G014100 | 0.00 | 38.16 | -6.25 | 1.09E-10 | 1.42E-08 | -//- |
| Glyma.13G015400 | 0.00 | 94.21 | -7.56 | 1.60E-22 | 5.34E-20 | -//- |
| Glyma.13G016700 | 0.00 | 83.07 | -9.25 | 6.16E-17 | 1.32E-14 | -//- |
| Glyma.13G021200 | 0.00 | 20.05 | -9.20 | 3.76E-05 | 1.81E-03 | -//- |
| Glyma.13G023300 | 0.00 | 155.72 | -8.98 | 2.47E-31 | 1.38E-28 | -//- |
| Glyma.13G024100 | 1.61 | 152.82 | -6.57 | 9.80E-38 | 7.57E-35 | -//- |
| Glyma.13G042500 | 8.24 | 32.47 | -1.98 | 5.98E-05 | 2.69E-03 | -//- |
| Glyma.13G043000 | 0.00 | 44.21 | -10.34 | 1.68E-08 | 1.63E-06 | white-brown complex homolog protein 11 |
| Glyma.13G043800 | 46.47 | 15.45 | 1.59 | 9.09E-05 | 3.89E-03 | white-brown complex homolog protein 11 |
| Glyma.13G045100 | 1.26 | 23.34 | -4.21 | 1.18E-06 | 8.12E-05 | GDSL-like Lipase/Acylhydrolase superfamily protein |
| Glyma.13G054800 | 731.49 | 356.95 | 1.04 | 5.82E-28 | 2.70E-25 | -//- |
| Glyma.13G056100 | 54.03 | 19.95 | 1.44 | 9.31E-05 | 3.96E-03 | HXXXD-type acyl-transferase family protein |
| Glyma.13G059500 | 5.60 | 32.93 | -2.55 | 2.62E-06 | 1.69E-04 | early nodulin-like protein 17 |
| Glyma.13G068800 | 76.10 | 23.38 | 1.70 | 1.33E-07 | 1.07E-05 | cytochrome P450, family 82, subfamily C, polypeptide 4 |
| Glyma.13G070900 | 22.52 | 118.19 | -2.39 | 9.21E-18 | 2.14E-15 | Peroxidase superfamily protein |
| Glyma.13G075700 | 24.16 | 77.27 | -1.68 | 2.81E-08 | 2.63E-06 | Bifunctional inhibitor/lipid-transfer protein/seed storage 2S albumin superfamily protein |
| Glyma.13G082300 | 45.93 | 133.51 | -1.54 | 6.10E-12 | 9.02E-10 | flavonol synthase 1 |
| Glyma.13G084000 | 150.77 | 70.27 | 1.10 | 1.54E-07 | 1.22E-05 | nitrate reductase 1 |
| Glyma.13G089600 | 33.93 | 82.72 | -1.29 | 2.02E-06 | 1.32E-04 | Outer arm dynein light chain 1 protein |
| Glyma.13G091200 | 175.58 | 550.97 | -1.65 | 1.12E-48 | 1.30E-45 | Fatty acid hydroxylase superfamily |
| Glyma.13G091500 | 36.29 | 8.81 | 2.04 | 3.49E-05 | 1.71E-03 | sequence-specific DNA binding transcription factors |
| Glyma.13G094100 | 0.00 | 19.73 | -6.37 | 3.61E-06 | 2.25E-04 | CAP (Cysteine-rich secretory proteins, Antigen 5, and Pathogenesis-related 1 protein) superfamily protein |
| Glyma.13G095600 | 115.78 | 43.75 | 1.40 | 1.96E-08 | 1.89E-06 | 4-coumarate:CoA ligase 2 |
| Glyma.13G103200 | 1.91 | 18.21 | -3.25 | 8.63E-05 | 3.71E-03 | -//- |
| Glyma.13G109800 | 121.55 | 50.89 | 1.26 | 1.44E-07 | 1.15E-05 | oxophytodienoate-reductase 3 |
| Glyma.13G112500 | 12.37 | 60.97 | -2.30 | 1.64E-09 | 1.84E-07 | chaperonin 20 |
| Glyma.13G113000 | 88.27 | 38.87 | 1.18 | 1.99E-05 | 1.04E-03 | -//- |
| Glyma.13G119000 | 143.32 | 42.09 | 1.77 | 1.02E-13 | 1.78E-11 | P-glycoprotein 11 |
| Glyma.13G131400 | 11.14 | 108.40 | -3.28 | 6.73E-22 | 2.14E-19 | NAD(P)-binding Rossmann-fold superfamily protein |
| Glyma.13G132100 | 39.30 | 80.35 | -1.03 | 7.47E-05 | 3.28E-03 | Histone superfamily protein |
| Glyma.13G160400 | 34.75 | 419.62 | -3.59 | 1.34E-85 | 3.74E-82 | Bifunctional inhibitor/lipid-transfer protein/seed storage 2S albumin superfamily protein |
| Glyma.13G162600 | 86.56 | 32.01 | 1.44 | 7.79E-07 | 5.53E-05 | monogalactosyldiacylglycerol synthase 2 |
| Glyma.13G175300 | 16.47 | 47.07 | -1.51 | 5.40E-05 | 2.47E-03 | -//- |
| Glyma.13G186200 | 43.30 | 13.26 | 1.71 | 6.74E-05 | 3.01E-03 | 12-oxophytodienoate reductase 2 |
| Glyma.13G186600 | 38.38 | 10.11 | 1.92 | 4.40E-05 | 2.07E-03 | zinc induced facilitator-like 1 |
| Glyma.13G199500 | 0.00 | 26.31 | -5.89 | 7.52E-08 | 6.41E-06 | annexin 8 |
| Glyma.13G199800 | 28.19 | 102.35 | -1.86 | 7.49E-12 | 1.10E-09 | annexin 8 |
| Glyma.13G200300 | 73.44 | 572.47 | -2.96 | 5.33E-99 | 1.71E-95 | -//- |
| Glyma.13G207900 | 16.54 | 50.71 | -1.62 | 1.16E-05 | 6.44E-04 | dehydratase family |
| Glyma.13G214600 | 41.72 | 125.90 | -1.59 | 7.85E-12 | 1.15E-09 | acyl carrier protein 4 |
| Glyma.13G231300 | 43.91 | 145.00 | -1.72 | 9.35E-15 | 1.75E-12 | GDSL-like Lipase/Acylhydrolase superfamily protein |
| Glyma.13G233600 | 1.54 | 18.39 | -3.58 | 4.15E-05 | 1.97E-03 | GDSL-like Lipase/Acylhydrolase superfamily protein |
| Glyma.13G236100 | 17.87 | 191.40 | -3.42 | 1.29E-38 | 1.03E-35 | -//- |
| Glyma.13G237000 | 114.41 | 49.48 | 1.21 | 7.57E-07 | 5.39E-05 | Dormancy/auxin associated family protein/dormancy-associated protein-like 1 |
| Glyma.13G244800 | 7.96 | 38.80 | -2.28 | 1.67E-06 | 1.11E-04 | glycine-rich protein |
| Glyma.13G255800 | 7.55 | 35.01 | -2.21 | 8.01E-06 | 4.67E-04 | UDP-glucosyl transferase 78D2 |
| Glyma.13G261200 | 82.18 | 35.72 | 1.20 | 3.03E-05 | 1.50E-03 | Calcium-dependent lipid-binding (CaLB domain) family protein |
| Glyma.13G267800 | 346.06 | 776.81 | -1.17 | 9.44E-42 | 8.38E-39 | gamma-glutamyl hydrolase 1 |
| Glyma.13G267900 | 711.12 | 1473.10 | -1.05 | 2.36E-66 | 4.29E-63 | gamma-glutamyl hydrolase 3 |
| Glyma.13G275800 | 22.18 | 59.20 | -1.42 | 1.59E-05 | 8.55E-04 | early nodulin-like protein 1 |
| Glyma.13G304400 | 4.85 | 25.07 | -2.37 | 8.42E-05 | 3.64E-03 | xyloglucan endotransglucosylase/hydrolase 9 |
| Glyma.13G307000 | 2.29 | 19.27 | -3.07 | 8.11E-05 | 3.52E-03 | Peroxidase superfamily protein |
| Glyma.13G312900 | 153.91 | 61.04 | 1.33 | 5.07E-10 | 6.03E-08 | BEL1-like homeodomain 1 |
| Glyma.13G321700 | 317.19 | 644.93 | -1.02 | 6.77E-29 | 3.36E-26 | serine carboxypeptidase-like 51 |
| Glyma.13G324900 | 57.85 | 17.40 | 1.73 | 3.18E-06 | 2.02E-04 | Nucleotide/sugar transporter family protein |
| Glyma.13G327100 | 104.19 | 49.26 | 1.08 | 1.77E-05 | 9.35E-04 | FASCICLIN-like arabinogalactan-protein 11 |
| Glyma.13G334500 | 153.09 | 67.27 | 1.19 | 1.79E-08 | 1.73E-06 | UDP-XYL synthase 5 |
| Glyma.13G341500 | 40.46 | 166.79 | -2.04 | 1.65E-20 | 4.74E-18 | Leucine-rich repeat (LRR) family protein |
| Glyma.13G347800 | 212.58 | 454.77 | -1.10 | 5.00E-23 | 1.74E-20 | lipoxygenase 1 |
| Glyma.13G350000 | 3.83 | 33.28 | -3.12 | 1.85E-07 | 1.46E-05 | Protein kinase superfamily protein |
| Glyma.13G361300 | 0.00 | 18.07 | -5.14 | 9.23E-06 | 5.24E-04 | cadmium tolerance 1 |
| Glyma.13G361900 | 127.22 | 54.53 | 1.22 | 1.43E-07 | 1.15E-05 | pleiotropic drug resistance 12 |
| Glyma.13G362800 | 0.00 | 13.76 | -6.65 | 1.20E-04 | 4.98E-03 | Protein of unknown function, DUF642 |
| Glyma.14G032000 | 19.72 | 130.96 | -2.73 | 2.86E-22 | 9.31E-20 | -//- |
| Glyma.14G037300 | 39.57 | 79.79 | -1.01 | 1.02E-04 | 4.29E-03 | Late embryogenesis abundant protein, group 2 |
| Glyma.14G041500 | 163.34 | 81.38 | 1.01 | 4.45E-07 | 3.29E-05 | Ethylene insensitive 3 family protein |
| Glyma.14G043500 | 10.15 | 46.51 | -2.20 | 3.01E-07 | 2.28E-05 | 3-oxo-5-alpha-steroid 4-dehydrogenase family protein |
| Glyma.14G045200 | 0.00 | 14.39 | -5.40 | 7.10E-05 | 3.14E-03 | HSP20-like chaperones superfamily protein |
| Glyma.14G048000 | 25.83 | 154.83 | -2.58 | 1.18E-24 | 4.66E-22 | Subtilisin-like serine endopeptidase family protein |
| Glyma.14G049500 | 285.82 | 133.61 | 1.10 | 5.92E-13 | 9.73E-11 | ethylene-forming enzyme |
| Glyma.14G052000 | 0.00 | 43.25 | -7.31 | 2.35E-11 | 3.29E-09 | glycine-rich protein 3 short isoform |
| Glyma.14G053500 | 4.48 | 24.15 | -2.43 | 8.98E-05 | 3.84E-03 | -//- |
| Glyma.14G062300 | 30.93 | 2.12 | 3.87 | 9.91E-08 | 8.27E-06 | Laccase/Diphenol oxidase family protein |
| Glyma.14G064500 | 0.00 | 275.08 | -9.27 | 4.16E-52 | 5.43E-49 | Subtilisin-like serine endopeptidase family protein |
| Glyma.14G064600 | 2.29 | 1125.20 | -8.94 | 3.36E-216 | 3.51E-212 | Subtilase family protein |
| Glyma.14G068600 | 0.00 | 21.93 | -5.42 | 9.36E-07 | 6.51E-05 | Radical SAM superfamily protein |
| Glyma.14G070600 | 20.37 | 71.26 | -1.81 | 2.07E-08 | 1.98E-06 | beta-galactosidase 5 |
| Glyma.14G071100 | 4.68 | 29.53 | -2.66 | 5.54E-06 | 3.31E-04 | TRAM, LAG1 and CLN8 (TLC) lipid-sensing domain containing protein |
| Glyma.14G087500 | 165.09 | 43.64 | 1.92 | 2.66E-17 | 5.85E-15 | Subtilase family protein |
| Glyma.14G115500 | 258.10 | 597.12 | -1.21 | 3.05E-34 | 1.99E-31 | Bifunctional inhibitor/lipid-transfer protein/seed storage 2S albumin superfamily protein |
| Glyma.14G126200 | 125.17 | 338.81 | -1.44 | 1.82E-25 | 7.59E-23 | Protein of unknown function, DUF642 |
| Glyma.14G140900 | 1.54 | 784.27 | -8.99 | 2.53E-150 | 1.06E-146 | BURP domain-containing protein |
| Glyma.14G141000 | 32.40 | 694.30 | -4.42 | 1.86E-158 | 1.11E-154 | BURP domain-containing protein |
| Glyma.14G158500 | 0.00 | 16.23 | -6.31 | 2.60E-05 | 1.32E-03 | -//- |
| Glyma.14G164900 | 16.37 | 90.50 | -2.47 | 2.14E-14 | 3.93E-12 | nitrate reductase 1 |
| Glyma.14G200900 | 110.75 | 271.61 | -1.29 | 5.05E-18 | 1.20E-15 | O-methyltransferase family protein |
| Glyma.14G201000 | 11.72 | 37.63 | -1.68 | 1.03E-04 | 4.34E-03 | O-methyltransferase family protein |
| Glyma.14G201800 | 38.92 | 10.36 | 1.91 | 4.26E-05 | 2.01E-03 | Peroxidase superfamily protein |
| Glyma.14G203900 | 0.00 | 28.01 | -5.09 | 3.47E-08 | 3.20E-06 | expansin A8 |
| Glyma.14G209100 | 196.73 | 93.05 | 1.08 | 3.72E-09 | 3.95E-07 | -//- |
| Glyma.14G212500 | 33.66 | 7.00 | 2.27 | 2.03E-05 | 1.06E-03 | STELAR K+ outward rectifier |
| Glyma.14G219100 | 29.97 | 5.59 | 2.42 | 2.77E-05 | 1.39E-03 | GAST1 protein homolog 1 |
| Glyma.14G220600 | 6.73 | 29.00 | -2.11 | 7.99E-05 | 3.47E-03 | GDSL-like Lipase/Acylhydrolase superfamily protein |
| Glyma.14G223000 | 1.61 | 42.51 | -4.73 | 1.77E-11 | 2.53E-09 | SKU5 similar 5 |
| Glyma.15G008800 | 16.23 | 70.63 | -2.12 | 6.34E-10 | 7.43E-08 | Embryo-specific protein 3, (ATS3) |
| Glyma.15G011900 | 107.06 | 47.43 | 1.17 | 3.01E-06 | 1.92E-04 | pleiotropic drug resistance 12 |
| Glyma.15G012600 | 0.00 | 29.50 | -8.75 | 2.76E-07 | 2.10E-05 | cadmium tolerance 1 |
| Glyma.15G024200 | 0.00 | 51.21 | -7.38 | 4.15E-13 | 6.95E-11 | Glucose-methanol-choline (GMC) oxidoreductase family protein |
| Glyma.15G026400 | 83.59 | 296.76 | -1.83 | 9.48E-31 | 5.21E-28 | lipoxygenase 1 |
| Glyma.15G031400 | 139.73 | 50.64 | 1.46 | 1.82E-10 | 2.30E-08 | beta glucosidase 15 |
| Glyma.15G056900 | 18.97 | 66.06 | -1.80 | 7.25E-08 | 6.20E-06 | -//- |
| Glyma.15G068900 | 1.95 | 30.20 | -3.95 | 5.60E-08 | 4.93E-06 | glycine-rich protein |
| Glyma.15G077600 | 3.28 | 39.26 | -3.58 | 2.11E-09 | 2.34E-07 | -//- |
| Glyma.15G081300 | 3.45 | 53.90 | -3.97 | 3.85E-13 | 6.47E-11 | GDSL-like Lipase/Acylhydrolase superfamily protein |
| Glyma.15G081600 | 88.98 | 40.25 | 1.14 | 3.10E-05 | 1.53E-03 | TRICHOME BIREFRINGENCE-LIKE 38 |
| Glyma.15G089000 | 11.17 | 997.71 | -6.48 | 5.33E-237 | 7.42E-233 | GDSL-like Lipase/Acylhydrolase superfamily protein |
| Glyma.15G105100 | 125.28 | 62.21 | 1.01 | 9.03E-06 | 5.15E-04 | Aluminium induced protein with YGL and LRDR motifs |
| Glyma.15G107100 | 59.56 | 16.83 | 1.82 | 9.52E-07 | 6.60E-05 | MATE efflux family protein |
| Glyma.15G108000 | 253.66 | 124.91 | 1.02 | 1.74E-10 | 2.22E-08 | starch synthase 3 |
| Glyma.15G110300 | 62.23 | 22.81 | 1.45 | 2.47E-05 | 1.26E-03 | WRKY family transcription factor |
| Glyma.15G112500 | 110.72 | 40.03 | 1.47 | 1.30E-08 | 1.28E-06 | fibrillin |
| Glyma.15G118500 | 19.10 | 68.11 | -1.83 | 3.11E-08 | 2.89E-06 | Heavy metal transport/detoxification superfamily protein |
| Glyma.15G123800 | 43.81 | 116.28 | -1.41 | 1.71E-09 | 1.91E-07 | Eukaryotic aspartyl protease family protein |
| Glyma.15G139800 | 21.12 | 0.00 | 5.40 | 2.23E-06 | 1.45E-04 | -//- |
| Glyma.15G141600 | 212.35 | 83.89 | 1.34 | 2.35E-13 | 4.02E-11 | -//- |
| Glyma.15G143600 | 135.42 | 42.09 | 1.69 | 2.85E-12 | 4.40E-10 | chitinase-like protein 2 |
| Glyma.15G144000 | 0.00 | 22.03 | -5.46 | 8.73E-07 | 6.11E-05 | myb domain protein 111 |
| Glyma.15G156400 | 118.99 | 53.05 | 1.17 | 1.00E-06 | 6.93E-05 | VACUOLAR SORTING RECEPTOR 6 |
| Glyma.15G157100 | 74.94 | 21.18 | 1.82 | 3.86E-08 | 3.52E-06 | cellulose synthase A4 |
| Glyma.15G169100 | 280.49 | 61.64 | 2.19 | 2.52E-33 | 1.53E-30 | xyloglucan endotransglycosylase 6 |
| Glyma.15G176900 | 110.38 | 301.78 | -1.45 | 3.60E-23 | 1.26E-20 | germin 3 |
| Glyma.15G177800 | 345.96 | 137.15 | 1.33 | 1.11E-20 | 3.23E-18 | Cysteine proteinases superfamily protein |
| Glyma.15G180900 | 68.21 | 150.02 | -1.14 | 5.43E-09 | 5.68E-07 | chaperonin 20 |
| Glyma.15G184400 | 25.97 | 72.78 | -1.49 | 7.27E-07 | 5.20E-05 | Plant invertase/pectin methylesterase inhibitor superfamily |
| Glyma.15G215100 | 61.61 | 24.72 | 1.32 | 9.88E-05 | 4.18E-03 | basic helix-loop-helix (bHLH) DNA-binding superfamily protein |
| Glyma.15G215600 | 4.31 | 26.45 | -2.62 | 1.97E-05 | 1.04E-03 | Leucine-rich repeat protein kinase family protein |
| Glyma.15G228900 | 0.00 | 32.85 | -6.59 | 2.66E-09 | 2.90E-07 | UBX domain-containing protein |
| Glyma.15G234300 | 1.20 | 18.67 | -3.96 | 1.92E-05 | 1.01E-03 | Eukaryotic aspartyl protease family protein |
| Glyma.15G239200 | 0.00 | 133.12 | -8.93 | 3.32E-27 | 1.47E-24 | -//- |
| Glyma.15G239300 | 0.00 | 76.04 | -10.12 | 5.34E-14 | 9.53E-12 | -//- |
| Glyma.15G239500 | 20.71 | 194.55 | -3.23 | 1.62E-37 | 1.21E-34 | -//- |
| Glyma.15G239600 | 4.99 | 235.40 | -5.56 | 3.16E-58 | 4.71E-55 | -//- |
| Glyma.15G239700 | 8.00 | 281.37 | -5.14 | 1.50E-68 | 2.98E-65 | -//- |
| Glyma.15G250500 | 157.40 | 362.71 | -1.20 | 2.66E-21 | 8.09E-19 | TCP-1/cpn60 chaperonin family protein |
| Glyma.15G251500 | 257.52 | 89.76 | 1.52 | 4.99E-19 | 1.28E-16 | glutathione S-transferase TAU 19 |
| Glyma.15G257300 | 74.84 | 30.31 | 1.30 | 2.09E-05 | 1.08E-03 | 2-oxoglutarate (2OG) and Fe(II)-dependent oxygenase superfamily protein |
| Glyma.15G262600 | 95.07 | 43.11 | 1.14 | 1.75E-05 | 9.29E-04 | Uncharacterized conserved protein (DUF2358) |
| Glyma.15G267000 | 14.66 | 128.66 | -3.13 | 9.50E-25 | 3.81E-22 | ATP-citrate lyase A-1 |
| Glyma.15G276000 | 0.00 | 15.95 | -4.06 | 6.97E-05 | 3.09E-03 | topoisomerase II |
| Glyma.15G276100 | 5.64 | 28.89 | -2.36 | 2.57E-05 | 1.30E-03 | Protein of unknown function, DUF642 |
| Glyma.16G007700 | 137.13 | 319.46 | -1.22 | 2.73E-19 | 7.15E-17 | germin 3/germin-like protein 1 |
| Glyma.16G026900 | 49.62 | 14.96 | 1.73 | 1.65E-05 | 8.82E-04 | Auxin-responsive GH3 family protein |
| Glyma.16G027200 | 66.64 | 27.23 | 1.29 | 6.81E-05 | 3.03E-03 | SPA (suppressor of phyA-105) protein family |
| Glyma.16G033700 | 80.85 | 184.36 | -1.19 | 2.23E-11 | 3.13E-09 | UDP-glucosyl transferase 73B1/UDP-glycosyltransferase 73B4 |
| Glyma.16G043400 | 49.21 | 158.69 | -1.69 | 1.29E-15 | 2.49E-13 | apyrase 2 |
| Glyma.16G043500 | 52.59 | 255.70 | -2.28 | 1.07E-34 | 7.10E-32 | apyrase 2 |
| Glyma.16G045000 | 5.09 | 42.58 | -3.06 | 4.93E-09 | 5.19E-07 | xyloglucan endotransglucosylase/hydrolase 5 |
| Glyma.16G049000 | 352.35 | 172.69 | 1.03 | 3.83E-14 | 6.92E-12 | CCR-like |
| Glyma.16G058500 | 88.40 | 42.01 | 1.07 | 8.47E-05 | 3.66E-03 | -//- |
| Glyma.16G095000 | 576.62 | 253.57 | 1.19 | 9.15E-28 | 4.19E-25 | Plant protein of unknown function (DUF827) |
| Glyma.16G100400 | 65.37 | 147.41 | -1.17 | 3.21E-09 | 3.43E-07 | low-molecular-weight cysteine-rich 69 |
| Glyma.16G102400 | 3.49 | 24.05 | -2.79 | 2.66E-05 | 1.34E-03 | Sec14p-like phosphatidylinositol transfer family protein |
| Glyma.16G113000 | 9.91 | 60.02 | -2.60 | 1.54E-10 | 1.99E-08 | hydroxy methylglutaryl CoA reductase 1 |
| Glyma.16G131200 | 16.95 | 0.00 | 5.58 | 2.17E-05 | 1.12E-03 | ferulic acid 5-hydroxylase 1 |
| Glyma.16G131800 | 2.49 | 23.80 | -3.25 | 7.11E-06 | 4.19E-04 | beta-galactosidase 3 |
| Glyma.16G150300 | 2.73 | 25.57 | -3.23 | 3.54E-06 | 2.21E-04 | xyloglucan endotransglucosylase/hydrolase 5 |
| Glyma.16G157100 | 71.69 | 24.58 | 1.54 | 1.95E-06 | 1.27E-04 | sucrose-proton symporter 1/sucrose-proton symporter 2 |
| Glyma.16G172600 | 10.32 | 59.59 | -2.53 | 3.23E-10 | 3.92E-08 | multidrug resistance-associated protein 14 |
| Glyma.16G180500 | 61.41 | 161.13 | -1.39 | 2.08E-12 | 3.25E-10 | HXXXD-type acyl-transferase family protein |
| Glyma.16G204600 | 138.81 | 60.19 | 1.21 | 5.53E-08 | 4.88E-06 | Enolase |
| Glyma.16G211700 | 34.10 | 145.39 | -2.09 | 1.44E-18 | 3.54E-16 | Kunitz family trypsin and protease inhibitor protein |
| Glyma.17G012300 | 3.62 | 45.73 | -3.66 | 7.34E-11 | 9.78E-09 | SKU5 similar 4 |
| Glyma.17G015400 | 0.00 | 53.30 | -7.02 | 6.85E-14 | 1.21E-11 | cytochrome P450, family 77, subfamily B, polypeptide 1 |
| Glyma.17G016800 | 7.45 | 34.13 | -2.20 | 1.15E-05 | 6.41E-04 | Heavy metal transport/detoxification superfamily protein |
| Glyma.17G020600 | 163.89 | 62.53 | 1.39 | 3.38E-11 | 4.65E-09 | pyruvate orthophosphate dikinase |
| Glyma.17G031600 | 0.00 | 22.53 | -6.78 | 9.35E-07 | 6.51E-05 | myb domain protein 111 |
| Glyma.17G041200 | 89.91 | 31.12 | 1.53 | 1.21E-07 | 9.85E-06 | P-glycoprotein 11 |
| Glyma.17G047200 | 12.58 | 55.52 | -2.14 | 3.50E-08 | 3.21E-06 | chaperonin 20 |
| Glyma.17G049800 | 17.33 | 145.04 | -3.07 | 3.51E-27 | 1.54E-24 | germin 3 |
| Glyma.17G064600 | 111.16 | 40.95 | 1.44 | 1.97E-08 | 1.89E-06 | 4-coumarate:CoA ligase 2 |
| Glyma.17G067400 | 14.83 | 50.57 | -1.77 | 3.19E-06 | 2.02E-04 | diacylglycerol kinase 7 |
| Glyma.17G069100 | 577.30 | 1289.90 | -1.16 | 1.37E-67 | 2.60E-64 | Fatty acid hydroxylase superfamily |
| Glyma.17G072200 | 54.88 | 14.54 | 1.92 | 1.10E-06 | 7.55E-05 | Cellulose synthase family protein |
| Glyma.17G080400 | 68.07 | 23.73 | 1.52 | 4.62E-06 | 2.80E-04 | transporter associated with antigen processing protein 2 |
| Glyma.17G092400 | 160.13 | 360.59 | -1.17 | 2.26E-20 | 6.46E-18 | spermidine synthase 1 |
| Glyma.17G099800 | 7.96 | 38.73 | -2.28 | 1.74E-06 | 1.14E-04 | myb domain protein 94 |
| Glyma.17G111100 | 5.16 | 39.19 | -2.92 | 3.99E-08 | 3.64E-06 | Bifunctional inhibitor/lipid-transfer protein/seed storage 2S albumin superfamily protein |
| Glyma.17G114700 | 178.69 | 87.46 | 1.03 | 6.78E-08 | 5.86E-06 | SPX domain gene 2 |
| Glyma.17G119000 | 1.88 | 34.06 | -4.18 | 4.67E-09 | 4.93E-07 | -//- |
| Glyma.17G127000 | 40.60 | 145.32 | -1.84 | 5.43E-16 | 1.10E-13 | GDSL-like Lipase/Acylhydrolase superfamily protein |
| Glyma.17G127900 | 15.92 | 44.60 | -1.49 | 1.06E-04 | 4.45E-03 | -//- |
| Glyma.17G131200 | 0.00 | 14.29 | -4.80 | 9.30E-05 | 3.96E-03 | GRAS family transcription factor |
| Glyma.17G138300 | 23.41 | 62.95 | -1.43 | 7.67E-06 | 4.49E-04 | Cupredoxin superfamily protein |
| Glyma.17G148400 | 18.28 | 53.33 | -1.54 | 1.32E-05 | 7.26E-04 | ureidoglycine aminohydrolase |
| Glyma.17G165200 | 9.16 | 43.32 | -2.24 | 5.57E-07 | 4.04E-05 | natural resistance-associated macrophage protein 3 |
| Glyma.17G197700 | 74.77 | 29.67 | 1.33 | 1.49E-05 | 8.08E-04 | -//- |
| Glyma.17G208200 | 81.84 | 286.43 | -1.81 | 2.56E-29 | 1.29E-26 | Protein of unknown function, DUF642 |
| Glyma.17G212200 | 73.37 | 228.50 | -1.64 | 5.53E-21 | 1.66E-18 | Bifunctional inhibitor/lipid-transfer protein/seed storage 2S albumin superfamily protein |
| Glyma.17G224300 | 104.50 | 43.39 | 1.27 | 8.88E-07 | 6.21E-05 | Concanavalin A-like lectin protein kinase family protein |
| Glyma.17G230500 | 161.33 | 66.77 | 1.27 | 9.03E-10 | 1.03E-07 | metallothionein 2A |
| Glyma.17G236800 | 281.13 | 90.25 | 1.64 | 6.42E-23 | 2.21E-20 | Subtilase family protein |
| Glyma.17G237100 | 37.45 | 157.56 | -2.07 | 8.82E-20 | 2.47E-17 | Gibberellin-regulated family protein |
| Glyma.17G242900 | 63.87 | 23.94 | 1.42 | 2.70E-05 | 1.35E-03 | phosphatidic acid phosphohydrolase 2 |
| Glyma.17G253500 | 5.47 | 36.50 | -2.74 | 2.89E-07 | 2.20E-05 | TRAM, LAG1 and CLN8 (TLC) lipid-sensing domain containing protein |
| Glyma.18G005800 | 204.42 | 472.35 | -1.21 | 2.23E-27 | 1.01E-24 | glycosylphosphatidylinositol-anchored lipid protein transfer 1 |
| Glyma.18G009700 | 122.03 | 49.19 | 1.31 | 4.86E-08 | 4.34E-06 | glyceraldehyde-3-phosphate dehydrogenase C subunit 1/glyceraldehyde-3-phosphate dehydrogenase C2 |
| Glyma.18G011600 | 37.93 | 263.51 | -2.80 | 4.07E-44 | 4.02E-41 | beta-ketoacyl reductase 1 |
| Glyma.18G030700 | 0.00 | 25.99 | -7.57 | 2.96E-07 | 2.24E-05 | cellulase 2 |
| Glyma.18G045300 | 1.13 | 25.46 | -4.50 | 2.54E-07 | 1.95E-05 | Uncharacterised protein family (UPF0497) |
| Glyma.18G050600 | 65.92 | 25.64 | 1.36 | 3.52E-05 | 1.72E-03 | Ribosomal protein L16p/L10e family protein |
| Glyma.18G059600 | 38.65 | 8.13 | 2.25 | 5.48E-06 | 3.28E-04 | Leucine-rich repeat protein kinase family protein |
| Glyma.18G062000 | 16.57 | 61.93 | -1.90 | 6.53E-08 | 5.68E-06 | fatty acid desaturase 8 |
| Glyma.18G067200 | 181.97 | 88.27 | 1.04 | 3.69E-08 | 3.37E-06 | alpha-glucan phosphorylase 2 |
| Glyma.18G073500 | 1.06 | 22.49 | -4.41 | 1.41E-06 | 9.48E-05 | kinesin-like protein 1 |
| Glyma.18G080000 | 157.81 | 69.10 | 1.19 | 9.64E-09 | 9.65E-07 | serine acetyltransferase 2;2 |
| Glyma.18G090900 | 709.55 | 272.25 | 1.38 | 6.59E-43 | 5.98E-40 | Eukaryotic aspartyl protease family protein |
| Glyma.18G098400 | 28.23 | 64.61 | -1.19 | 7.05E-05 | 3.12E-03 | alpha/beta-Hydrolases superfamily protein |
| Glyma.18G103000 | 53.34 | 13.37 | 2.00 | 7.80E-07 | 5.53E-05 | TRICHOME BIREFRINGENCE-LIKE 43 |
| Glyma.18G106400 | 0.00 | 48.17 | -7.88 | 5.83E-12 | 8.66E-10 | protodermal factor 1 |
| Glyma.18G113100 | 49.93 | 140.47 | -1.49 | 5.15E-12 | 7.68E-10 | spermidine hydroxycinnamoyl transferase |
| Glyma.18G138000 | 1.74 | 36.46 | -4.39 | 8.52E-10 | 9.82E-08 | polygalacturonase 2 |
| Glyma.18G139900 | 9.26 | 41.55 | -2.17 | 1.57E-06 | 1.05E-04 | Pectin lyase-like superfamily protein |
| Glyma.18G146900 | 7.59 | 101.61 | -3.74 | 1.28E-22 | 4.33E-20 | Protein-tyrosine phosphatase-like, PTPLA |
| Glyma.18G153500 | 9.12 | 102.91 | -3.50 | 7.26E-22 | 2.28E-19 | expansin A4 |
| Glyma.18G186800 | 93.12 | 45.48 | 1.03 | 9.38E-05 | 3.99E-03 | NAD(P)-binding Rossmann-fold superfamily protein |
| Glyma.18G195200 | 50.98 | 16.06 | 1.67 | 2.14E-05 | 1.11E-03 | receptor like protein 13 |
| Glyma.18G203100 | 23.92 | 2.62 | 3.19 | 1.44E-05 | 7.87E-04 | Plant protein of unknown function (DUF828) |
| Glyma.18G207900 | 0.00 | 19.77 | -4.53 | 5.38E-06 | 3.23E-04 | SEC14 cytosolic factor family protein / phosphoglyceride transfer family protein |
| Glyma.18G210900 | 26.69 | 303.23 | -3.51 | 2.87E-61 | 4.44E-58 | lipid transfer protein 1 |
| Glyma.18G222200 | 8.03 | 63.76 | -2.99 | 1.47E-12 | 2.34E-10 | FASCICLIN-like arabinogalactan protein 8 |
| Glyma.18G236700 | 0.00 | 41.24 | -6.15 | 1.84E-11 | 2.61E-09 | Pectin lyase-like superfamily protein |
| Glyma.18G239500 | 32.09 | 6.79 | 2.24 | 3.59E-05 | 1.74E-03 | Subtilisin-like serine endopeptidase family protein |
| Glyma.18G250100 | 24.19 | 85.44 | -1.82 | 6.89E-10 | 8.05E-08 | Subtilisin-like serine endopeptidase family protein |
| Glyma.18G250700 | 10.05 | 35.90 | -1.84 | 5.76E-05 | 2.61E-03 | Subtilisin-like serine endopeptidase family protein |
| Glyma.18G258000 | 7.35 | 29.39 | -2.00 | 1.21E-04 | 4.98E-03 | HXXXD-type acyl-transferase family protein |
| Glyma.18G259400 | 9.81 | 91.81 | -3.23 | 1.53E-18 | 3.73E-16 | GAST1 protein homolog 4 |
| Glyma.18G260900 | 1.03 | 18.25 | -4.15 | 1.86E-05 | 9.81E-04 | expansin A1 |
| Glyma.18G266200 | 0.00 | 15.03 | -4.97 | 5.60E-05 | 2.55E-03 | Histone superfamily protein |
| Glyma.18G289000 | 85.40 | 40.46 | 1.08 | 1.06E-04 | 4.43E-03 | ACT domain-containing protein |
| Glyma.18G290000 | 48.25 | 15.14 | 1.67 | 3.40E-05 | 1.67E-03 | Acyl-CoA N-acyltransferases (NAT) superfamily protein |
| Glyma.19G008200 | 75.21 | 26.28 | 1.52 | 1.52E-06 | 1.02E-04 | Tetratricopeptide repeat (TPR)-like superfamily protein |
| Glyma.19G015500 | 96.37 | 207.28 | -1.11 | 1.98E-11 | 2.80E-09 | -//- |
| Glyma.19G018600 | 120.25 | 47.50 | 1.34 | 3.49E-08 | 3.21E-06 | AAA-ATPase 1 |
| Glyma.19G020000 | 129.24 | 60.44 | 1.10 | 1.29E-06 | 8.80E-05 | SPFH/Band 7/PHB domain-containing membrane-associated protein family |
| Glyma.19G021400 | 1.61 | 22.99 | -3.84 | 2.67E-06 | 1.72E-04 | basic helix-loop-helix (bHLH) DNA-binding family protein |
| Glyma.19G028500 | 82.66 | 36.85 | 1.17 | 4.57E-05 | 2.13E-03 | UDP-glucose 6-dehydrogenase family protein |
| Glyma.19G028800 | 16.71 | 59.91 | -1.84 | 1.93E-07 | 1.52E-05 | biotin carboxyl carrier protein 2 |
| Glyma.19G034900 | 12.27 | 43.50 | -1.83 | 1.03E-05 | 5.82E-04 | Pollen Ole e 1 allergen and extensin family protein |
| Glyma.19G045800 | 168.43 | 80.67 | 1.06 | 7.56E-08 | 6.44E-06 | Rhomboid-related intramembrane serine protease family protein |
| Glyma.19G046000 | 16.23 | 52.55 | -1.70 | 3.99E-06 | 2.45E-04 | O-acyltransferase (WSD1-like) family protein |
| Glyma.19G050000 | 3.08 | 33.03 | -3.43 | 6.61E-08 | 5.72E-06 | GDSL-like Lipase/Acylhydrolase superfamily protein |
| Glyma.19G050800 | 1.78 | 18.07 | -3.35 | 7.55E-05 | 3.31E-03 | GDSL-like Lipase/Acylhydrolase superfamily protein |
| Glyma.19G051000 | 0.00 | 17.22 | -5.28 | 1.43E-05 | 7.83E-04 | GDSL-like Lipase/Acylhydrolase superfamily protein |
| Glyma.19G051100 | 5.37 | 261.60 | -5.61 | 1.57E-64 | 2.72E-61 | GDSL-like Lipase/Acylhydrolase superfamily protein |
| Glyma.19G057300 | 3.42 | 24.69 | -2.85 | 1.66E-05 | 8.88E-04 | cytochrome P450, family 96, subfamily A, polypeptide 1 |
| Glyma.19G064200 | 708.32 | 309.20 | 1.20 | 3.30E-34 | 2.09E-31 | -//- |
| Glyma.19G064300 | 94.90 | 42.12 | 1.17 | 1.15E-05 | 6.41E-04 | -//- |
| Glyma.19G105100 | 9.81 | 464.07 | -5.56 | 5.68E-113 | 1.98E-109 | Chalcone and stilbene synthase family protein |
| Glyma.19G142700 | 80.65 | 8.49 | 3.25 | 9.88E-16 | 1.92E-13 | -//- |
| Glyma.19G151000 | 5.43 | 29.50 | -2.44 | 1.45E-05 | 7.89E-04 | Disease resistance-responsive (dirigent-like protein) family protein |
| Glyma.19G151400 | 0.00 | 17.40 | -4.35 | 2.34E-05 | 1.21E-03 | gamma histone variant H2AX |
| Glyma.19G164300 | 76.37 | 21.43 | 1.83 | 2.51E-08 | 2.37E-06 | phosphate transporter 1;7 |
| Glyma.19G166500 | 273.38 | 79.68 | 1.78 | 5.85E-25 | 2.37E-22 | Long-chain fatty alcohol dehydrogenase family protein |
| Glyma.19G172800 | 17.05 | 322.15 | -4.24 | 4.12E-73 | 9.55E-70 | NAD(P)-binding Rossmann-fold superfamily protein |
| Glyma.19G181000 | 21.70 | 70.02 | -1.69 | 1.08E-07 | 8.89E-06 | -//- |
| Glyma.19G187000 | 145.13 | 71.65 | 1.02 | 1.49E-06 | 9.98E-05 | UDP-glucosyl transferase 73C2 |
| Glyma.19G192800 | 385.43 | 170.89 | 1.17 | 8.47E-19 | 2.12E-16 | starch branching enzyme 2.1/starch branching enzyme 2.2 |
| Glyma.19G212800 | 158.35 | 52.20 | 1.60 | 3.65E-13 | 6.19E-11 | sucrose synthase 3 |
| Glyma.19G218300 | 2.94 | 732.00 | -7.96 | 2.24E-157 | 1.04E-153 | AMP-dependent synthetase and ligase family protein |
| Glyma.19G223000 | 121.14 | 18.64 | 2.70 | 3.00E-19 | 7.78E-17 | Glycosyl hydrolase superfamily protein |
| Glyma.19G234100 | 0.00 | 16.02 | -4.29 | 5.24E-05 | 2.41E-03 | histone H2A 7 |
| Glyma.19G240400 | 37.25 | 76.46 | -1.04 | 1.04E-04 | 4.39E-03 | copper/zinc superoxide dismutase 1 |
| Glyma.19G244100 | 3.55 | 30.52 | -3.10 | 6.35E-07 | 4.55E-05 | HXXXD-type acyl-transferase family protein |
| Glyma.19G245400 | 71.56 | 29.04 | 1.30 | 3.27E-05 | 1.61E-03 | pathogenesis-related 4 |
| Glyma.19G246000 | 79.01 | 31.16 | 1.34 | 7.61E-06 | 4.47E-04 | TPX2 (targeting protein for Xklp2) protein family |
| Glyma.19G250000 | 0.00 | 14.11 | -5.37 | 8.41E-05 | 3.64E-03 | Li-tolerant lipase 1 |
| Glyma.19G250200 | 10.05 | 150.98 | -3.91 | 1.05E-33 | 6.47E-31 | Li-tolerant lipase 1 |
| Glyma.19G256400 | 8.92 | 37.52 | -2.07 | 8.90E-06 | 5.09E-04 | Eukaryotic aspartyl protease family protein |
| Glyma.19G258700 | 46.03 | 8.74 | 2.40 | 2.45E-07 | 1.89E-05 | cytochrome P450, family 78, subfamily A, polypeptide 6 |
| Glyma.19G259800 | 41.76 | 85.69 | -1.04 | 4.02E-05 | 1.92E-03 | 60S acidic ribosomal protein family |
| Glyma.20G008300 | 114.41 | 44.42 | 1.36 | 4.80E-08 | 4.31E-06 | -//- |
| Glyma.20G017900 | 5.84 | 49.44 | -3.08 | 2.63E-10 | 3.21E-08 | Polyketide cyclase/dehydrase and lipid transport superfamily protein |
| Glyma.20G018000 | 100.19 | 29.71 | 1.75 | 6.27E-10 | 7.38E-08 | phosphoglucomutase, putative / glucose phosphomutase, putative |
| Glyma.20G033900 | 30.00 | 132.45 | -2.14 | 1.65E-17 | 3.68E-15 | expansin A15 |
| Glyma.20G049800 | 2.05 | 90.11 | -5.46 | 2.62E-23 | 9.42E-21 | Bifunctional inhibitor/lipid-transfer protein/seed storage 2S albumin superfamily protein |
| Glyma.20G057500 | 81.98 | 199.50 | -1.28 | 1.74E-13 | 2.98E-11 | UDP-glucosyl transferase 85A2 |
| Glyma.20G077300 | 40.36 | 107.97 | -1.42 | 5.30E-09 | 5.55E-07 | Pectin lyase-like superfamily protein |
| Glyma.20G090500 | 50.64 | 15.56 | 1.70 | 1.69E-05 | 9.01E-04 | Pectinacetylesterase family protein |
| Glyma.20G109900 | 23.48 | 70.13 | -1.58 | 3.91E-07 | 2.93E-05 | GHMP kinase family protein |
| Glyma.20G113300 | 332.70 | 154.12 | 1.11 | 4.17E-15 | 7.85E-13 | P-loop containing nucleoside triphosphate hydrolases superfamily protein |
| Glyma.20G115500 | 171.48 | 549.37 | -1.68 | 1.05E-49 | 1.29E-46 | 3-ketoacyl-CoA synthase 6 |
| Glyma.20G129200 | 4.07 | 24.44 | -2.59 | 4.60E-05 | 2.14E-03 | Leucine-rich repeat protein kinase family protein |
| Glyma.20G129300 | 21.02 | 66.06 | -1.65 | 3.74E-07 | 2.81E-05 | Domain of unknown function (DUF303) |
| Glyma.20G151500 | 190.34 | 72.92 | 1.38 | 1.08E-12 | 1.76E-10 | ATP sulfurylase 1 |
| Glyma.20G152600 | 0.00 | 19.24 | -6.33 | 4.71E-06 | 2.85E-04 | subtilase 1.3 |
| Glyma.20G159200 | 7.52 | 31.48 | -2.07 | 4.91E-05 | 2.28E-03 | tubulin beta 8 |
| Glyma.20G162000 | 23.48 | 94.71 | -2.01 | 4.26E-12 | 6.49E-10 | detoxifying efflux carrier 35 |
| Glyma.20G167500 | 1548.61 | 687.09 | 1.17 | 2.01E-70 | 4.20E-67 | -//- |
| Glyma.20G179600 | 102.79 | 25.25 | 2.03 | 4.37E-12 | 6.63E-10 | PATATIN-like protein 6 |
| Glyma.20G188000 | 44.32 | 112.04 | -1.34 | 1.24E-08 | 1.22E-06 | cytochrome P450, family 77, subfamily A, polypeptide 4 |
| Glyma.20G204000 | 48.29 | 15.74 | 1.62 | 5.26E-05 | 2.41E-03 | phosphate transporter 1;7 |
| Glyma.20G238000 | 197.75 | 72.15 | 1.45 | 4.46E-14 | 8.03E-12 | phospholipase D P1 |
| Glyma.20G240300 | 25.05 | 118.72 | -2.24 | 1.11E-16 | 2.34E-14 | dehydroascorbate reductase 2 |
| Glyma.20G241700 | 2.67 | 81.77 | -4.94 | 6.08E-21 | 1.81E-18 | Chalcone-flavanone isomerase family protein |
| Glyma.20G246300 | 15.00 | 0.00 | 6.41 | 6.92E-05 | 3.07E-03 | inositol transporter 1 |
| Glyma.20G247400 | 17.87 | 0.00 | 4.52 | 2.27E-05 | 1.17E-03 | Major facilitator superfamily protein |
| Glyma.U021100 | 14.69 | 44.91 | -1.61 | 3.80E-05 | 1.83E-03 | ROP interactive partner 5 |
| Glyma.U033600 | 0.00 | 20.72 | -4.39 | 3.75E-06 | 2.32E-04 | Histone superfamily protein |
| Novel00125 | 0.00 | 24.54 | -4.84 | 2.90E-07 | 2.21E-05 | -//- |

**STable 5. GO categories of DEGs**

| DEGs | GO term | **Ontology** | **Description** | **Number in input list** | **Number in BG/Ref** | **p-value** | **FDR** |
| --- | --- | --- | --- | --- | --- | --- | --- |
| Up-regulated | GO:0005976 | P | polysaccharide metabolic process | [14](http://bioinfo.cau.edu.cn/agriGO/termDetail.php?session=398503700&GO=GO:0005976) | 331 | 4.30E-08 | 1.40E-05 |
|  | GO:0006073 | P | cellular glucan metabolic process | [10](http://bioinfo.cau.edu.cn/agriGO/termDetail.php?session=398503700&GO=GO:0006073) | 159 | 1.20E-07 | 1.50E-05 |
|  | GO:0044042 | P | glucan metabolic process | [10](http://bioinfo.cau.edu.cn/agriGO/termDetail.php?session=398503700&GO=GO:0044042) | 161 | 1.40E-07 | 1.50E-05 |
|  | GO:0030243 | P | cellulose metabolic process | [7](http://bioinfo.cau.edu.cn/agriGO/termDetail.php?session=398503700&GO=GO:0030243) | 72 | 6.70E-07 | 4.30E-05 |
|  | GO:0030244 | P | cellulose biosynthetic process | [7](http://bioinfo.cau.edu.cn/agriGO/termDetail.php?session=398503700&GO=GO:0030244) | 70 | 5.60E-07 | 4.30E-05 |
|  | GO:0044264 | P | cellular polysaccharide metabolic process | [11](http://bioinfo.cau.edu.cn/agriGO/termDetail.php?session=398503700&GO=GO:0044264) | 293 | 3.80E-06 | 1.70E-04 |
|  | GO:0009250 | P | glucan biosynthetic process | [7](http://bioinfo.cau.edu.cn/agriGO/termDetail.php?session=398503700&GO=GO:0009250) | 93 | 3.30E-06 | 1.70E-04 |
|  | GO:0005975 | P | carbohydrate metabolic process | [24](http://bioinfo.cau.edu.cn/agriGO/termDetail.php?session=398503700&GO=GO:0005975) | 1443 | 1.70E-05 | 6.80E-04 |
|  | GO:0000271 | P | polysaccharide biosynthetic process | [8](http://bioinfo.cau.edu.cn/agriGO/termDetail.php?session=398503700&GO=GO:0000271) | 228 | 1.30E-04 | 4.10E-03 |
|  | GO:0033692 | P | cellular polysaccharide biosynthetic process | [8](http://bioinfo.cau.edu.cn/agriGO/termDetail.php?session=398503700&GO=GO:0033692) | 227 | 1.20E-04 | 4.10E-03 |
|  | GO:0044262 | P | cellular carbohydrate metabolic process | [14](http://bioinfo.cau.edu.cn/agriGO/termDetail.php?session=398503700&GO=GO:0044262) | 686 | 1.40E-04 | 4.20E-03 |
|  | GO:0055085 | P | transmembrane transport | [19](http://bioinfo.cau.edu.cn/agriGO/termDetail.php?session=398503700&GO=GO:0055085) | 1167 | 1.80E-04 | 4.70E-03 |
|  | GO:0055114 | P | oxidation reduction | [30](http://bioinfo.cau.edu.cn/agriGO/termDetail.php?session=398503700&GO=GO:0055114) | 2408 | 2.80E-04 | 6.90E-03 |
|  | GO:0016760 | F | cellulose synthase (UDP-forming) activity | [7](http://bioinfo.cau.edu.cn/agriGO/termDetail.php?session=398503700&GO=GO:0016760) | 60 | 2.10E-07 | 1.90E-05 |
|  | GO:0016757 | F | transferase activity, transferring glycosyl groups | [20](http://bioinfo.cau.edu.cn/agriGO/termDetail.php?session=398503700&GO=GO:0016757) | 797 | 2.40E-07 | 1.90E-05 |
|  | GO:0016759 | F | cellulose synthase activity | [7](http://bioinfo.cau.edu.cn/agriGO/termDetail.php?session=398503700&GO=GO:0016759) | 60 | 2.10E-07 | 1.90E-05 |
|  | GO:0016758 | F | transferase activity, transferring hexosyl groups | [18](http://bioinfo.cau.edu.cn/agriGO/termDetail.php?session=398503700&GO=GO:0016758) | 615 | 1.10E-07 | 1.90E-05 |
|  | GO:0046527 | F | glucosyltransferase activity | [7](http://bioinfo.cau.edu.cn/agriGO/termDetail.php?session=398503700&GO=GO:0046527) | 86 | 2.00E-06 | 1.10E-04 |
|  | GO:0035251 | F | UDP-glucosyltransferase activity | [7](http://bioinfo.cau.edu.cn/agriGO/termDetail.php?session=398503700&GO=GO:0035251) | 86 | 2.00E-06 | 1.10E-04 |
|  | GO:0022857 | F | transmembrane transporter activity | [20](http://bioinfo.cau.edu.cn/agriGO/termDetail.php?session=398503700&GO=GO:0022857) | 1020 | 9.30E-06 | 4.30E-04 |
|  | GO:0005215 | F | transporter activity | [23](http://bioinfo.cau.edu.cn/agriGO/termDetail.php?session=398503700&GO=GO:0005215) | 1340 | 1.60E-05 | 5.20E-04 |
|  | GO:0042626 | F | ATPase activity, coupled to transmembrane movement of substances | [7](http://bioinfo.cau.edu.cn/agriGO/termDetail.php?session=398503700&GO=GO:0042626) | 120 | 1.60E-05 | 5.20E-04 |
|  | GO:0043492 | F | ATPase activity, coupled to movement of substances | [7](http://bioinfo.cau.edu.cn/agriGO/termDetail.php?session=398503700&GO=GO:0043492) | 120 | 1.60E-05 | 5.20E-04 |
|  | GO:0015405 | F | P-P-bond-hydrolysis-driven transmembrane transporter activity | [7](http://bioinfo.cau.edu.cn/agriGO/termDetail.php?session=398503700&GO=GO:0015405) | 131 | 2.70E-05 | 7.30E-04 |
|  | GO:0015399 | F | primary active transmembrane transporter activity | [7](http://bioinfo.cau.edu.cn/agriGO/termDetail.php?session=398503700&GO=GO:0015399) | 131 | 2.70E-05 | 7.30E-04 |
|  | GO:0016820 | F | hydrolase activity, acting on acid anhydrides, catalyzing transmembrane movement of substances | [7](http://bioinfo.cau.edu.cn/agriGO/termDetail.php?session=398503700&GO=GO:0016820) | 133 | 3.00E-05 | 7.40E-04 |
|  | GO:0022804 | F | active transmembrane transporter activity | [11](http://bioinfo.cau.edu.cn/agriGO/termDetail.php?session=398503700&GO=GO:0022804) | 384 | 4.30E-05 | 9.20E-04 |
|  | GO:0003824 | F | catalytic activity | [115](http://bioinfo.cau.edu.cn/agriGO/termDetail.php?session=398503700&GO=GO:0003824) | 13905 | 4.20E-05 | 9.20E-04 |
|  | GO:0016887 | F | ATPase activity | [13](http://bioinfo.cau.edu.cn/agriGO/termDetail.php?session=398503700&GO=GO:0016887) | 581 | 1.00E-04 | 2.10E-03 |
|  | GO:0008194 | F | UDP-glycosyltransferase activity | [7](http://bioinfo.cau.edu.cn/agriGO/termDetail.php?session=398503700&GO=GO:0008194) | 180 | 1.90E-04 | 3.50E-03 |
|  | GO:0016020 | C | membrane | [45](http://bioinfo.cau.edu.cn/agriGO/termDetail.php?session=398503700&GO=GO:0016020) | 3518 | 2.90E-06 | 1.20E-04 |
|  | GO:0031224 | C | intrinsic to membrane | [24](http://bioinfo.cau.edu.cn/agriGO/termDetail.php?session=398503700&GO=GO:0031224) | 1453 | 1.90E-05 | 3.90E-04 |
|  | GO:0016021 | C | integral to membrane | [22](http://bioinfo.cau.edu.cn/agriGO/termDetail.php?session=398503700&GO=GO:0016021) | 1419 | 1.10E-04 | 1.50E-03 |
|  | GO:0044425 | C | membrane part | [24](http://bioinfo.cau.edu.cn/agriGO/termDetail.php?session=398503700&GO=GO:0044425) | 1801 | 4.80E-04 | 4.90E-03 |
| Down-regulated | GO:0006629 | P | lipid metabolic process | [41](http://bioinfo.cau.edu.cn/agriGO/termDetail.php?session=669425065&GO=GO:0006629) | 1024 | 2.80E-14 | 8.70E-12 |
|  | GO:0006633 | P | fatty acid biosynthetic process | [12](http://bioinfo.cau.edu.cn/agriGO/termDetail.php?session=669425065&GO=GO:0006633) | 108 | 1.80E-09 | 2.90E-07 |
|  | GO:0006631 | P | fatty acid metabolic process | [12](http://bioinfo.cau.edu.cn/agriGO/termDetail.php?session=669425065&GO=GO:0006631) | 130 | 1.20E-08 | 1.30E-06 |
|  | GO:0032787 | P | monocarboxylic acid metabolic process | [12](http://bioinfo.cau.edu.cn/agriGO/termDetail.php?session=669425065&GO=GO:0032787) | 176 | 2.80E-07 | 2.20E-05 |
|  | GO:0008152 | P | metabolic process | [176](http://bioinfo.cau.edu.cn/agriGO/termDetail.php?session=669425065&GO=GO:0008152) | 14225 | 1.80E-06 | 1.10E-04 |
|  | GO:0006457 | P | protein folding | [10](http://bioinfo.cau.edu.cn/agriGO/termDetail.php?session=669425065&GO=GO:0006457) | 181 | 1.60E-05 | 8.30E-04 |
|  | GO:0016053 | P | organic acid biosynthetic process | [12](http://bioinfo.cau.edu.cn/agriGO/termDetail.php?session=669425065&GO=GO:0016053) | 274 | 2.10E-05 | 8.30E-04 |
|  | GO:0046394 | P | carboxylic acid biosynthetic process | [12](http://bioinfo.cau.edu.cn/agriGO/termDetail.php?session=669425065&GO=GO:0046394) | 274 | 2.10E-05 | 8.30E-04 |
|  | GO:0044255 | P | cellular lipid metabolic process | [14](http://bioinfo.cau.edu.cn/agriGO/termDetail.php?session=669425065&GO=GO:0044255) | 418 | 7.60E-05 | 2.70E-03 |
|  | GO:0008610 | P | lipid biosynthetic process | [14](http://bioinfo.cau.edu.cn/agriGO/termDetail.php?session=669425065&GO=GO:0008610) | 470 | 2.50E-04 | 7.70E-03 |
|  | GO:0003824 | F | catalytic activity | [196](http://bioinfo.cau.edu.cn/agriGO/termDetail.php?session=669425065&GO=GO:0003824) | 13905 | 5.80E-14 | 1.70E-11 |
|  | GO:0016746 | F | transferase activity, transferring acyl groups | [21](http://bioinfo.cau.edu.cn/agriGO/termDetail.php?session=669425065&GO=GO:0016746) | 468 | 1.20E-08 | 1.20E-06 |
|  | GO:0016787 | F | hydrolase activity | [82](http://bioinfo.cau.edu.cn/agriGO/termDetail.php?session=669425065&GO=GO:0016787) | 4588 | 1.00E-08 | 1.20E-06 |
|  | GO:0016788 | F | hydrolase activity, acting on ester bonds | [30](http://bioinfo.cau.edu.cn/agriGO/termDetail.php?session=669425065&GO=GO:0016788) | 1054 | 1.90E-07 | 1.40E-05 |
|  | GO:0016747 | F | transferase activity, transferring acyl groups other than amino-acyl groups | [15](http://bioinfo.cau.edu.cn/agriGO/termDetail.php?session=669425065&GO=GO:0016747) | 360 | 3.60E-06 | 2.10E-04 |
|  | GO:0016758 | F | transferase activity, transferring hexosyl groups | [17](http://bioinfo.cau.edu.cn/agriGO/termDetail.php?session=669425065&GO=GO:0016758) | 615 | 1.30E-04 | 6.50E-03 |

Fig. S1. Gene expression analysis between dwarf mutant and control

1. Clustering of DEGs by hirachary clustering method. (B) Correlation analysis between dwarf mutant and control based on overall gene expression level. (C) DEGs identification with threshold of |log_2_ (fold change)|>1 and corrected P<0.005.


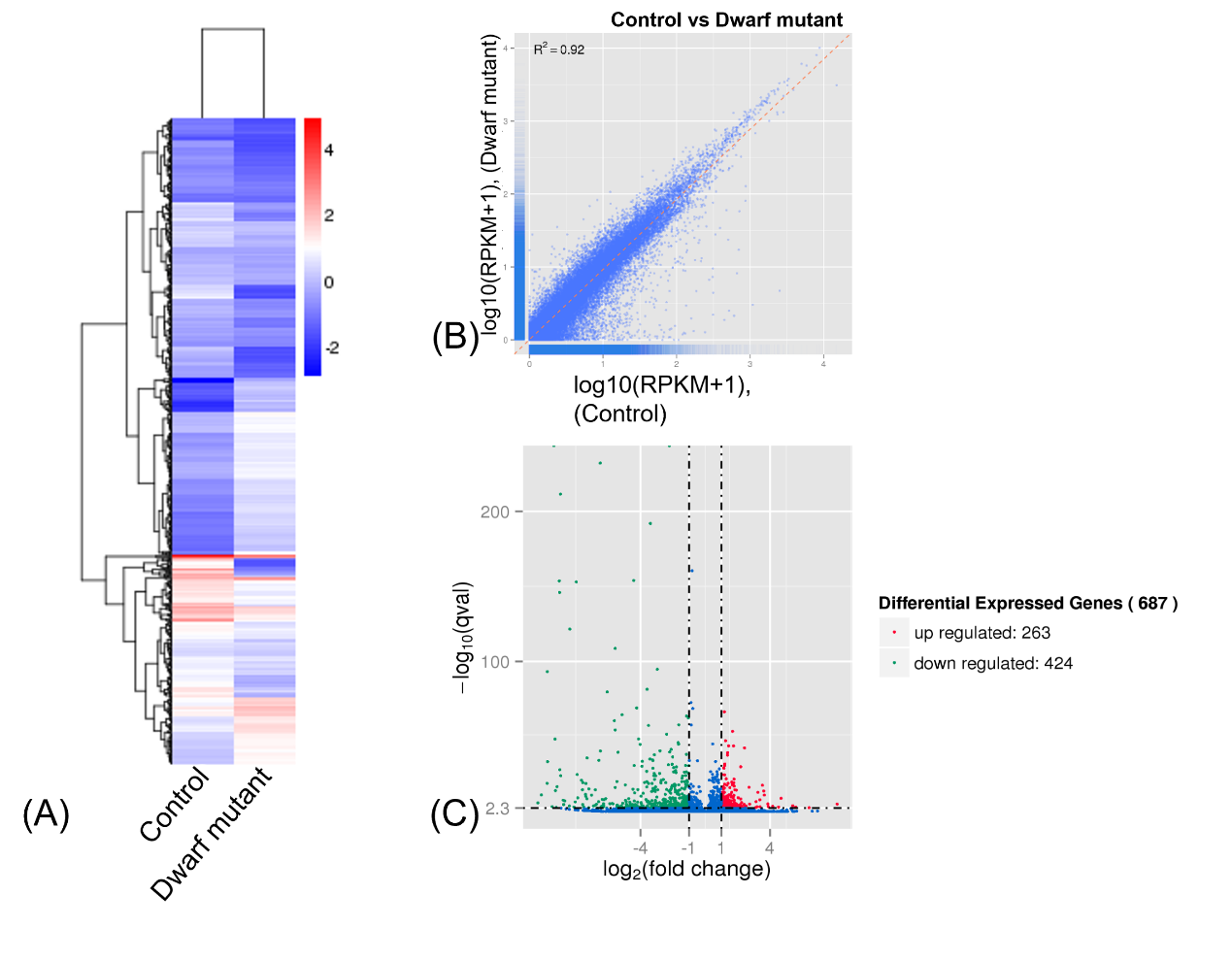


Fig. S2. MethylC-seq depth and coverage

Sequencing depth is displayed by histogram, scatterplot represents reads coverage. x axis: different chromosomes; left y axis: mean depth of reads aligned to corresponding chromosome; right y axis: reads coverage.


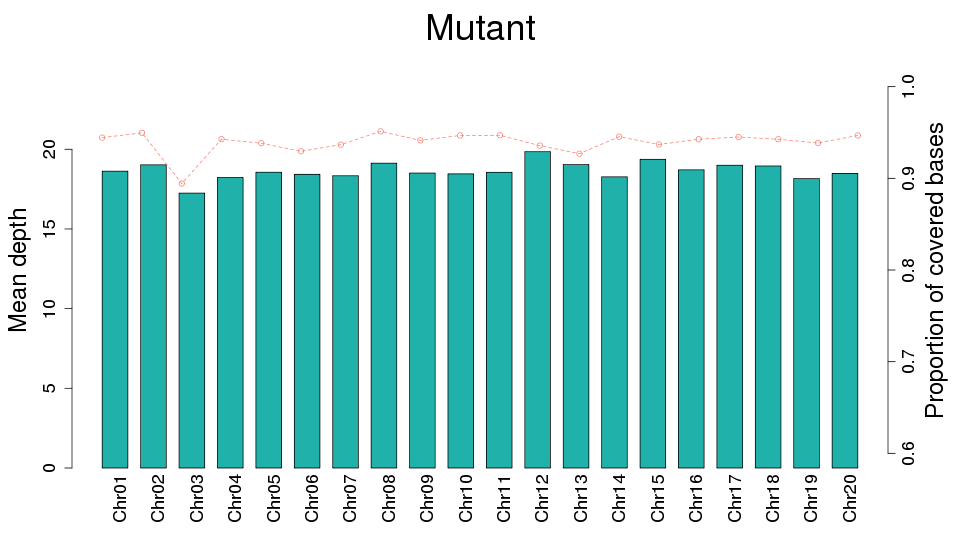

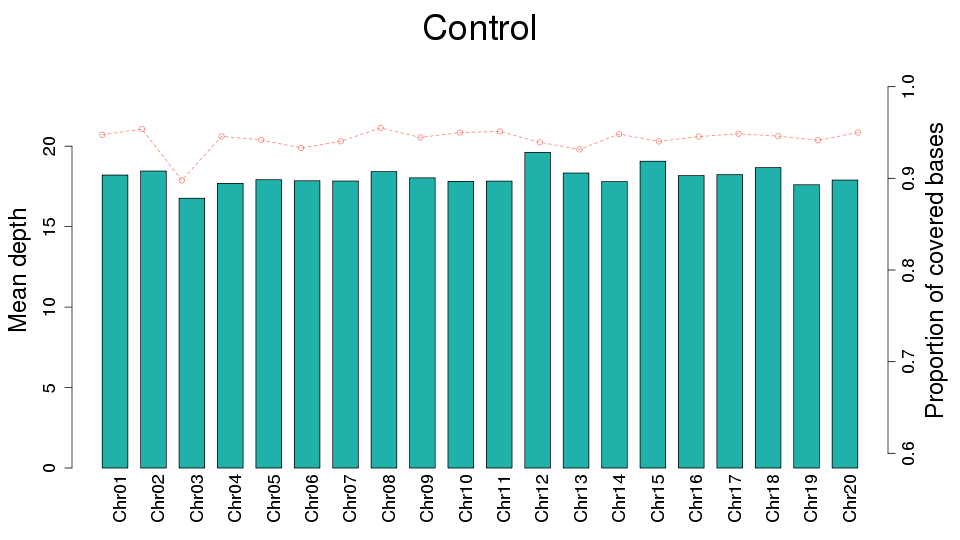


**Control**

**Dwarf mutant**

Supplemental Figure 1. Gene expression analysis between dwarf mutant and control

(A) Clustering of DEGs by hirachary clustering method. (B) Correlation analysis between dwarf mutant and control based on overall gene expression level. (C) DEGs identification with threshold of |log_2_ (fold change)|>1 and corrected P<0.005.
